# Rock properties and sediment caliber govern bedrock river morphology across the Taiwan Central Range

**DOI:** 10.1126/sciadv.adg6794

**Published:** 2023-11-15

**Authors:** Julia C. Carr, Roman A. DiBiase, En-Chao Yeh, Donald M. Fisher, Eric Kirby

**Affiliations:** ^1^ Department of Geosciences, Penn State University, State College, PA 16802, USA.; ^2^ Earth and Environmental Systems Institute, Pennsylvania State University, University Park, PA 16802, USA.; ^3^ Department of Earth Sciences, National Taiwan Normal University, Taipei City 106, Taiwan.; ^4^ Department of Earth, Marine, and Environmental Sciences, University of North Carolina at Chapel Hill, Chapel Hill, NC 27599, USA.

## Abstract

Feedbacks between surface and deep Earth processes in collisional mountain belts depend on how erosion and topographic relief vary in space and time. One outstanding unknown lies in how rock strength influences bedrock river morphology and thus mountain relief. Here, we quantify boulder cover and channel morphology using uncrewed aerial vehicle surveys along 30 kilometers of bedrock-bound river corridors throughout the Taiwan Central Range where regional gradients in rock properties relate to tectonic history. We find that boulder size systematically increases with increasing metamorphic grade and depth of exhumation. Boulder size correlates with reach-scale channel steepness but does not explain observations of highly variable channel width. Transport thresholds indicate that rivers are adjusted to mobilize boulders and are well in excess of the threshold to transport gravel and cobbles, as previously assumed. The linkage between metamorphic history, boulder size, and channel steepness reveals how rock properties can influence feedbacks between tectonics and topography throughout the life span of a mountain range.

## Teaser

Bedrock rivers in the Taiwan Central Range adjust their slopes to transport boulders whose size varies with tectonic history.

## INTRODUCTION

Quantifying potential feedbacks between surface and deep Earth processes requires understanding how climate and rock strength influence bedrock river incision and, ultimately, the relief of mountain ranges (*1*, *2*). Whereas climate parameters are readily measured and incorporated into surface process models (*3*), the challenge of assessing the relative importance of factors contributing to rock strength (e.g., rock tensile strength, mineralogy, and fracturing) impedes quantitative interpretations of tectonics from topography in all but the simplest tectonic settings (*4*). Furthermore, potential for a coordinated influence of rock strength on tectonics and erosion emerges from faulting and strain localization (*5*, *6*), interaction with near-surface topographic stresses (*7*, *8*), and/or changes in crystallinity and fabric development during metamorphism that depend on the subsurface exhumational trajectory of rocks (*9*). To assess the implications of the potential coevolution of exhumation pathways, rock strength, and topography in orogenic systems, proxies for rock strength measurable at the landscape scale are needed.

A principal challenge in characterizing how rock strength influences bedrock rivers arises from the complexity of local and nonlocal interactions between rock material properties, channel cross-sectional geometry (width and depth), channel slope, and sediment cover. River morphology can adjust through changes in both slope and width (*10*), and the partitioning between the two depends on sediment cover (*11*) and the erodibility of in-channel bedrock (*12*). Similarly, rock material strength sets the initial size distribution of sediment from hillslopes (*13*) through variations in lithology (*14*), fracture spacing (*15*, *16*), and the style of sediment delivery from hillslopes to channels (*17*). The role of boulders has been highlighted as a key linkage between bedrock fracture spacing, which sets boulder size (*15*), and channel width and slope, which are thought to be sensitive to boulder cover and size (*18*). These feedbacks between rock strength, channel cross-sectional geometry, and sediment cover (including boulders) make it challenging to develop mechanistic models of river incision that incorporate the effects of rock material properties (*19*, *20*).

Disentangling the interactions among rock material properties, sediment cover, and channel geometry requires field data that are both high resolution (e.g., to resolve bed sediment) and span large enough spatial scales to integrate across the reach-scale heterogeneity that is common to natural landscapes. Although quantifying channel width patterns in large rivers is possible using satellite remote sensing [e.g., (*20*–*23*)] or airborne lidar (*24*), narrow channels, overhanging walls, and spatially variable bank geometry limit the applicability of these approaches in steep upland catchments. Thus, measurements of channel geometry and sediment cover have typically been limited by field accessibility and time constraints. Advances in high-resolution remote sensing using uncrewed aerial vehicles (UAVs) and structure-from-motion photogrammetry now make it possible to bridge the gap between local field surveys of bedrock channels and regional models (*25*, *26*).

Here, we investigate how channel geometry and boulder size and cover reflect systematic gradients in rock properties across the Taiwan Central Range, using UAV surveys of bedrock river corridors. We first show how boulder size emerges as a key proxy for rock strength and how reach-scale (10^2^ to 10^3^ m) channel slopes appear adjusted to boulder transport thresholds. We then discuss challenges in reconciling reach-scale observations with orogen-scale patterns in relief and erosion rate and the implications for rock strength controls on channel incision.

### Geologic setting of the Taiwan Central Range

**Fig. 1.**
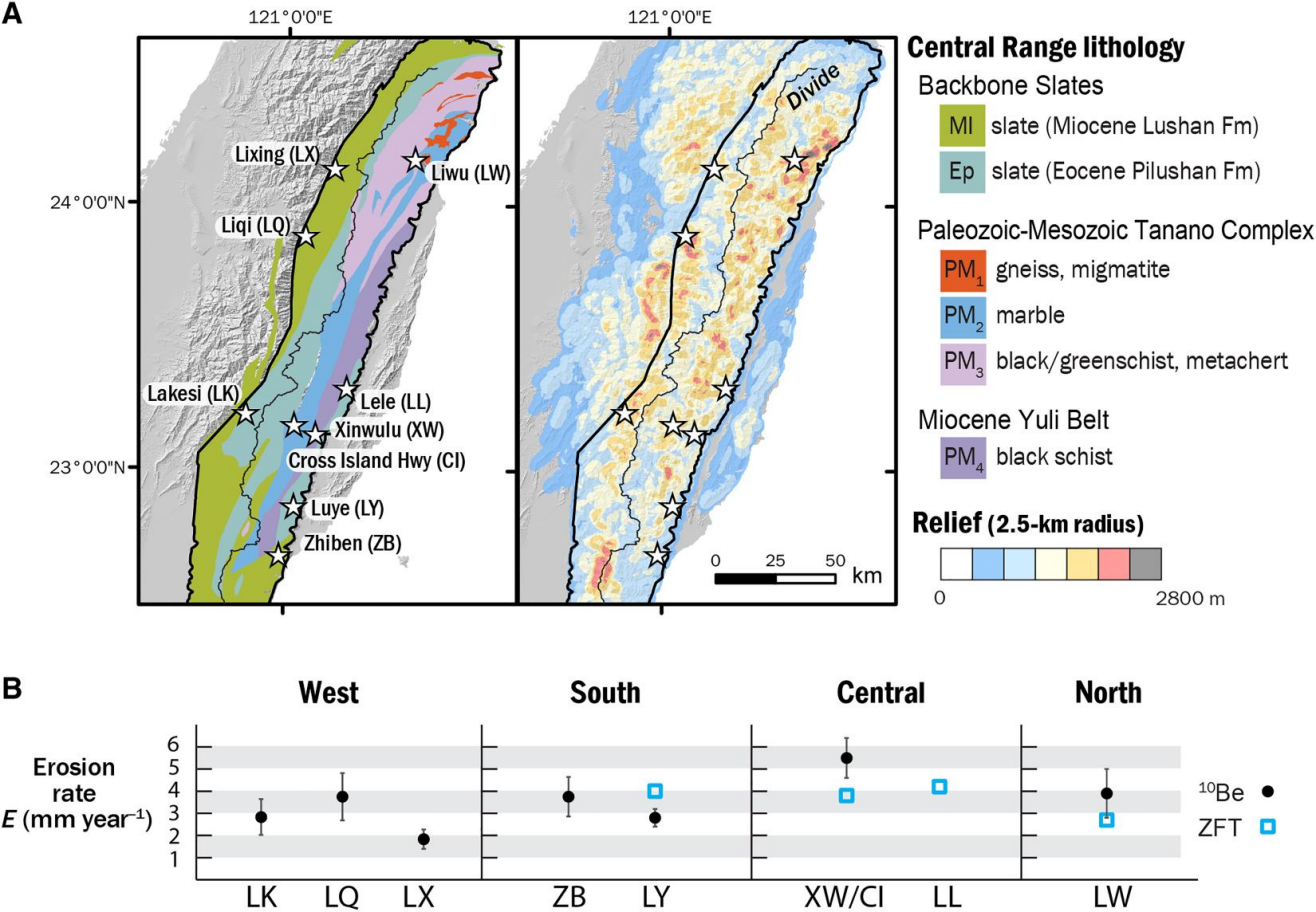
Overview map showing geology, topographic relief, and erosion rates of the Taiwan Central Range. (**A**) Map of study sites in the Taiwan Central Range showing the predominant lithology and relief. Central Range is outlined. (**B**) Regional summary of published erosion rates based on cosmogenic ^10^Be (*34*, *35*) and Zircon fission track (ZFT) (*35*) data for each study site.

The Taiwan Central Range provides an opportunity to evaluate how systematic gradients in metamorphic grade influence surface processes in a collisional setting. The Taiwan orogen developed during oblique collision of the Luzon volcanic arc (on the Philippine Sea Plate) with the passive Eurasian margin (*27*) and has been argued to be the archetype of a doubly vergent orogen (*28*). Differential exhumation across the mountain belt generated a metamorphic gradient from west to east, where contrasts in both burial depth and metamorphic fabric development across the main north-south topographic divide separate slates of the Western Central Range from schists and basement rocks of the Eastern Central Range (*9*). In addition, oblique collision drove exhumation of progressively deeper rocks northward along the Central Range (*29*). Consequently, lithostratigraphic formations exposed at the surface in the Central Range vary from the slates of the Miocene Lushan (Ml) and Eocene Pilushan (Ep) formations in the west and south to the Paleozoic-Mesozoic Tananao Complex (PM_1_ to PM_3_) and Yuli Belt (PM_4_) farther north in the Eastern Central Range (Fig. 1A). The Tananao complex is composed mainly of schists (PM_3_), with marbles (PM_2_) and gneisses (PM_1_) more prevalent to the north (*30*). The Yuli Belt is primarily composed of schists with limited marbles, and metamorphic phase assemblages are consistent with blueschist facies metamorphism followed by rapid exhumation of oceanic crust (*31*). Peak temperatures recorded by Raman spectroscopy of carbonaceous materials range from <330°C for the Slate belt of the Western Central Range and southern Eastern Central Range to 350° to 550°C in the Tananao Complex and reach 550°C in the Yuli Belt (*30*, *32*).

Exhumation and erosion rates across the Taiwan Central Range are constrained primarily from bedrock apatite and zircon fission track ages (*29*, *33*), from detrital zircon fission track populations, and from cosmogenic ^10^Be inventories in stream sediment (*34*, *35*). In general, erosion rates increase with increasing topographic relief in the southernmost Central Range, reaching a maximum at ~23°N latitude (Fig. 1); northward from this latitude, both relief and erosion rate remain relatively constant along the range. The combination of uniform topographic relief and relatively constant erosion and exhumation rates through time have led most authors to consider that the Central Range remained at or near erosional and topographic steady state over the past few million years (*35*).

In this study, we exploit the natural gradients in metamorphic grade (west to east) and exhumation depth (south to north) to test the influence of rock properties on the geomorphology of the Taiwanese orogen. We collected a series of high-resolution (1 to 5 cm) three-dimensional (3D) point clouds and orthomosaic imagery using UAV structure-from-motion photogrammetry surveys of bedrock-bound channels (*20*) with drainage areas ranging from 16 to 512 km^2^ and where fluvially scoured bedrock is present along both channel margins. Using these bedrock channel corridor surveys, we measured high-flow channel width and depth based on observed high water marks, we directly measured every boulder in the channel greater than 1 m in diameter, and we estimated boulder mobility using a simple Shields stress approach. The results were then summarized at the segment (every 20 m), reach (between tributary junctions), and regional scale.

We present channel geometry and boulder cover data from nine sites in four distinct regions: the Western Central Range (“west”) and three regions along the Eastern Central Range (“south,” “central,” and “north,” respectively) (Fig. 1). Broadly, we interpret that the metamorphic grade and depth of exhumation increases from south to north along the Eastern Central Range and that the Western Central Range is most comparable to the southern Eastern Central Range. To minimize the effect of variations in rock uplift rate, we focus on catchments in the mature part of the collision zone, where both topographic relief and erosion/exhumation rate remain constant along the range. We assume that climate forcing is similar across our study region; because of variable storm track direction (*36*), the Taiwan Central Range lacks a distinct rain shadow, and mean annual precipitation does not systematically vary from south to north. Erosion rates derived from detrital cosmogenic ^10^Be in the Western Central Range are 1 to 3 mm year^−1^, but these are likely lower bounds because of the downstream location of these samples (*34*). In the Eastern Central Range sites, erosion rates from ^10^Be and detrital zircon fission track data are 3 to 6 mm year^−1^ (*34*, *35*).

## RESULTS

### Channel morphology variation at the reach and orogen scale

**Fig. 2.**
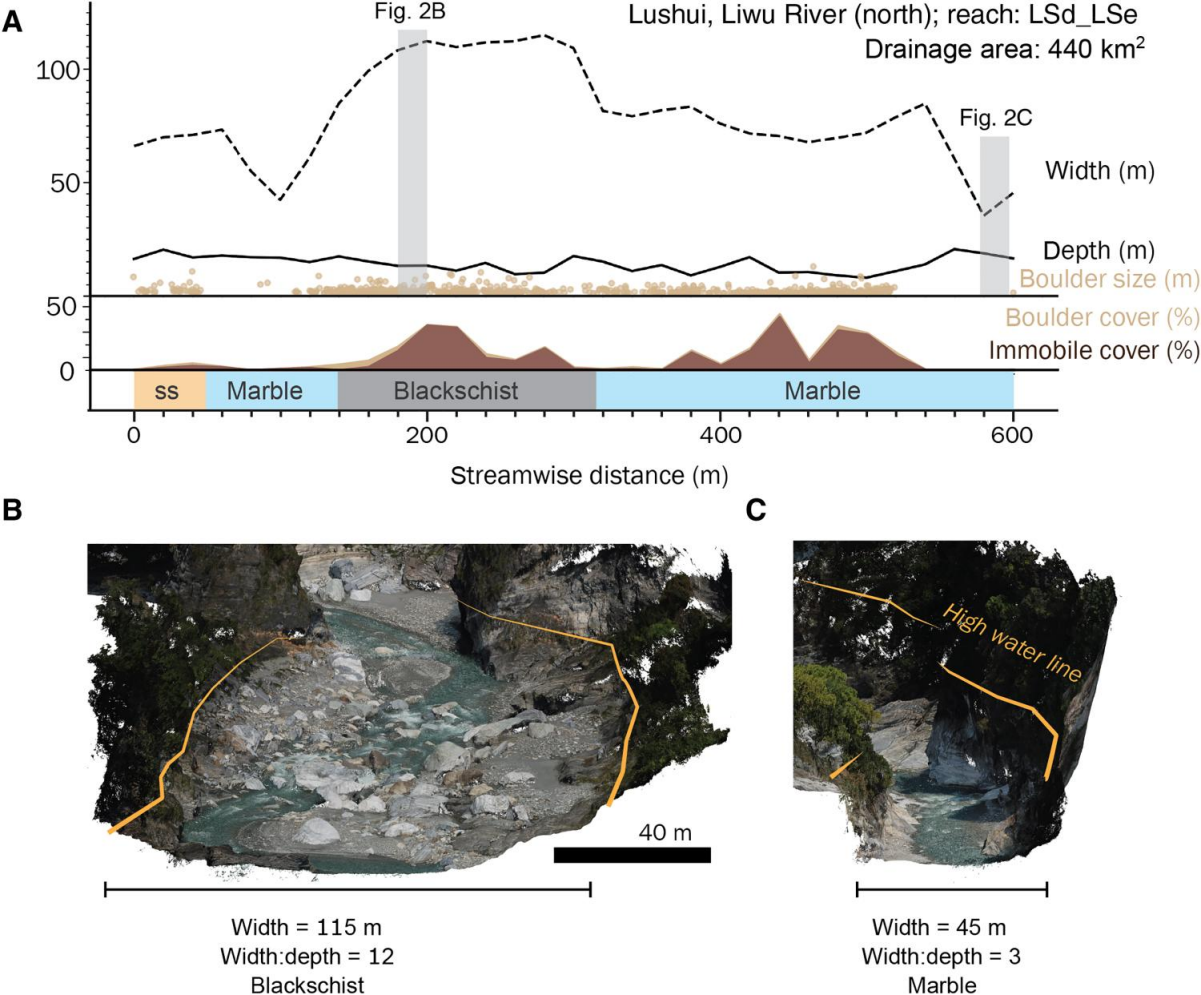
Example of channel geometry and boulder cover data from the Liwu River. (**A**) High-flow channel width and depth, boulder size, boulder cover for all boulders greater than 1 m in diameter, immobile boulder cover, and lithology plotted against downstream distance for the LSd_LSe reach of the Liwu River. Width, depth, and boulder cover data are sampled at 20-m intervals, and each boulder size point corresponds to the intermediate axis measurement of an individual boulder. Gray vertical shading highlights two locations with highly contrasting channel geometry. Local variation in width is tied to local lithology, with widening in blackschist bedrock over the comparatively stronger siliceous schist (ss) and marble. (**B** and **C**) Perspective views of colorized 3D point clouds of the highlighted locations with mapped high-water lines indicated.

**Fig. 3.**
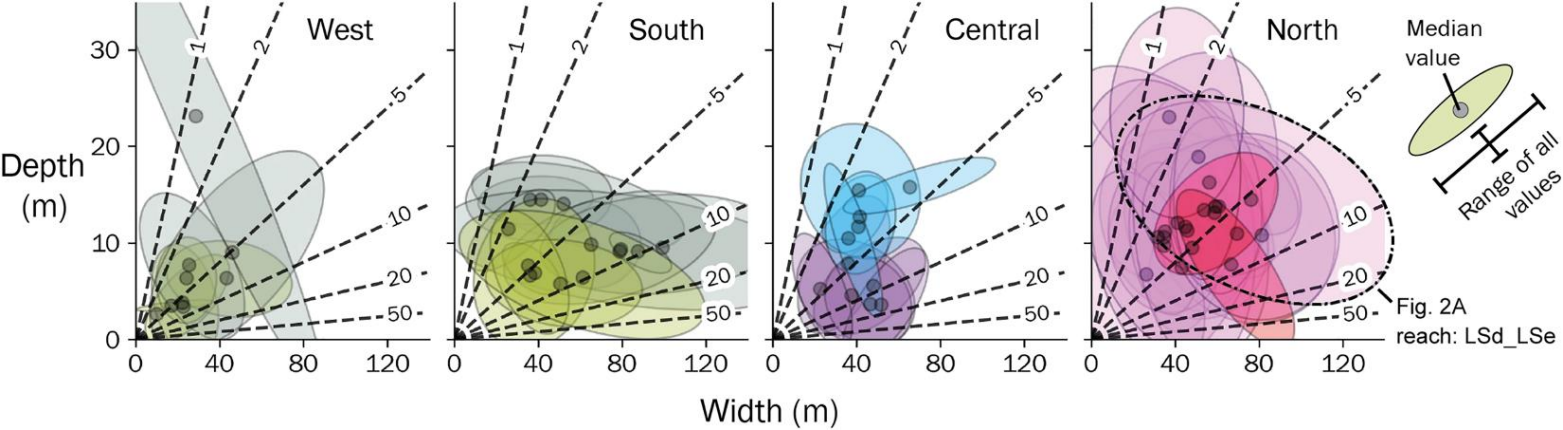
Summary of high-flow channel width and depth data mapped from UAV surveys. Points and ellipses indicate the median and range of values from individual channel reaches, colorized by the dominant regional geologic units for each reach (same colors as Fig. 1). Each ellipse represents a confidence ellipse with 3 SDs to the observed data in each reach. Dashed lines and labels indicate constant width-to-depth ratios. Bold ellipse indicates data from Liwu River highlighted in Fig. 2 (reach LSd_LSe).

Channel cross-sectional geometry measured from centimeter-scale UAV surveys is highly variable at local scales, and the range of variability within any reach is similar to that among reaches across the orogen. High-flow channel width broadly increases with increasing drainage area (fig. S7) but varies substantially (0.2× to 2× reach mean) over length scales of tens of meters (Fig. 2). High-flow channel depth varies from ~1 to 30 m but shows little sensitivity to drainage area across the studied reaches (fig. S8). The width-to-depth ratio of bedrock-bound channels across the orogen ranges from 1 to 100, with each reach showing a similar range of variability as the entire region (Fig. 3). No systematic patterns of width, depth, or width-to-depth ratio were observed across the orogen or when separated by lithostratigraphic units. At the reach scale, the channel width index, a measure of channel width that accounts for expected variations with drainage area (*10*), shows no correlation to the channel steepness index, a slope metric that is similarly normalized for drainage area (fig. S9) (*37*).

### Regional patterns in boulder size and immobile boulder coverage

**Fig. 4.**
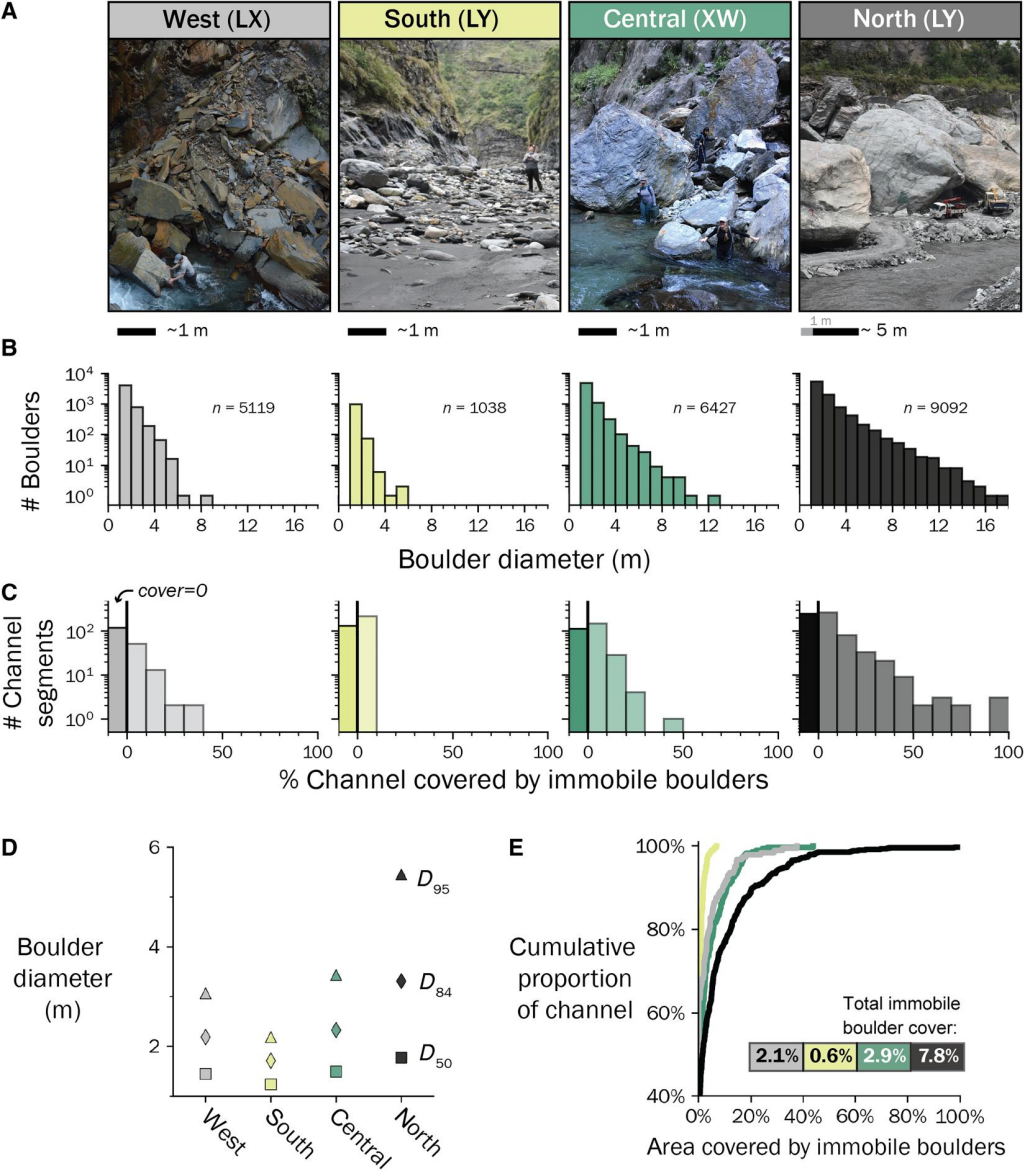
Summary of individual boulder size measurements by region. (**A**) Photographs of characteristic boulder size in each region with the authors and construction vehicles for scale. (**B**) Histograms showing the size distribution of boulders ≥1 m, in 1-m bins. *n* indicates number of boulders measured in each region. All distributions are significantly different (two-sample Kolmogorov-Smirnov test for each pair, *P* < 0.001). (**C**) Histograms of the number of channel segments with different percentages of immobile boulder coverage. The bar before zero indicates the frequency of segments with no immobile boulder coverage. All distributions are significantly different (two-sample Kolmogorov-Smirnov test, *P* < 0.001). (**D**) Summary of boulder size statistics by region, showing increasing boulder size from west to east and south to north. (**E**) Cumulative distribution of immobile cover for each region, showing increasing immobile boulder cover from south to north.

Boulder size increases systematically across the orogen from west to east and from south to north (Fig. 4). This pattern corresponds to gradients in metamorphic grade and depth of exhumation, with the Miocene and Eocene slates of the Western Central Range and southern Eastern Central Range having smaller and fewer boulders than the Tananao Complex and Yuli Belt of the Eastern Central Range (Fig. 4). Boulder size distributions differ between all regions based on pairwise two-sample Kolmogorov-Smirnov tests (*P* < 0.0001). While the west has larger boulders than the south, the contrast is primarily expressed through the coarsest boulder size fractions, with an otherwise broadly similar size distribution (fig. S11). Within individual regions, boulder size appears to correlate to specific lithologies; the largest grains in the east are composed of gneisses and quartzites, whereas the largest grains in the west derive from massive sandstones. However, these differences do not entirely explain regional variations, as the regional differences in boulder size hold even within similar lithologies (fig. S12).

The aerial coverage of immobile boulders in channels also systematically increases along these metamorphic gradients (Fig. 4, C and E). In the south, all channel segments have less than 10% immobile boulder cover, and 80% of channel segments have less than 1% immobile boulder cover. In the north, 23% of channel segments have more than 10% immobile boulder cover, and some reaches contain more than 50% immobile boulder cover. The west region has the highest fraction of channel segments with no immobile boulder cover (63%) but contains isolated segments with higher immobile boulder cover typically associated with localized landslide deposits (Fig. 4A). Overall, immobile boulder coverage is low, and 86% of all measured segments have less than 10% immobile boulder cover.

### Reach-scale connections between boulder cover and channel morphology

**Fig. 5.**
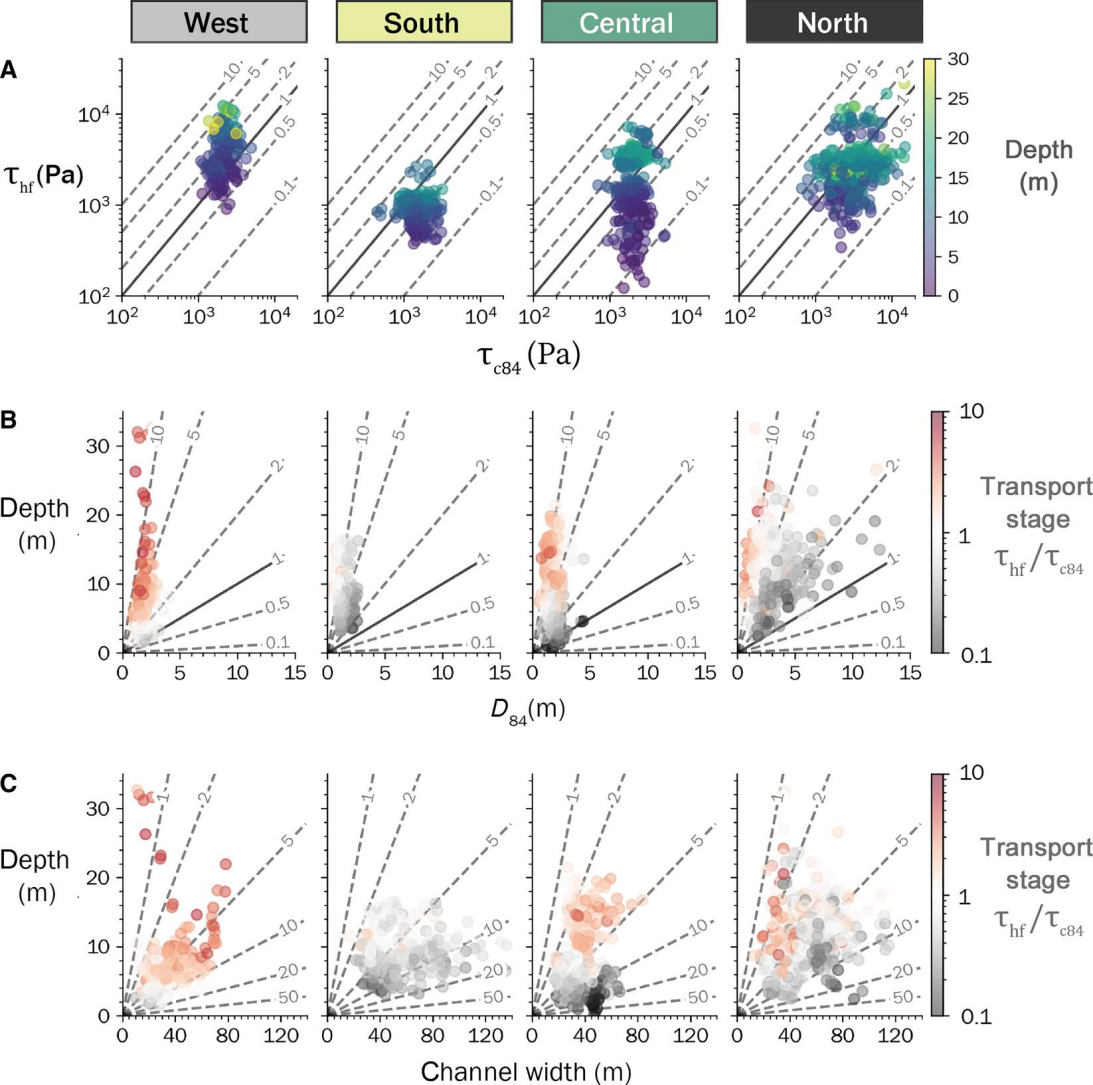
Summary of local transport stage by region. (**A**) High-flow bed shear stress, τ_hf_, plotted against the critical shear stress for *D*_84_ boulder motion, τ_c84_, at the scale of 20-m segments across the orogen. Colors indicate the median flow depth for each segment. Contour lines indicate constant transport stage ($\tau_{\mathrm{hf}}/\tau_{c84}$), where $\tau_{\mathrm{hf}}/\tau_{c84}=1$ is the initial motion threshold. (**B**) High-flow channel depth plotted against *D*_84_ boulder diameter, colored by transport stage ($\tau_{\mathrm{hf}}/\tau_{c84}$). Contour lines indicate relative submergence depth (*h*/*D*_84_), where values under one represent segments where *D*_84_ boulders protrude at high flow. (**C**) High-flow channel depth plotted against width, colored by transport stage. Contour lines indicate constant width-to-depth ratios as in Fig. 3.

**Fig. 6.**
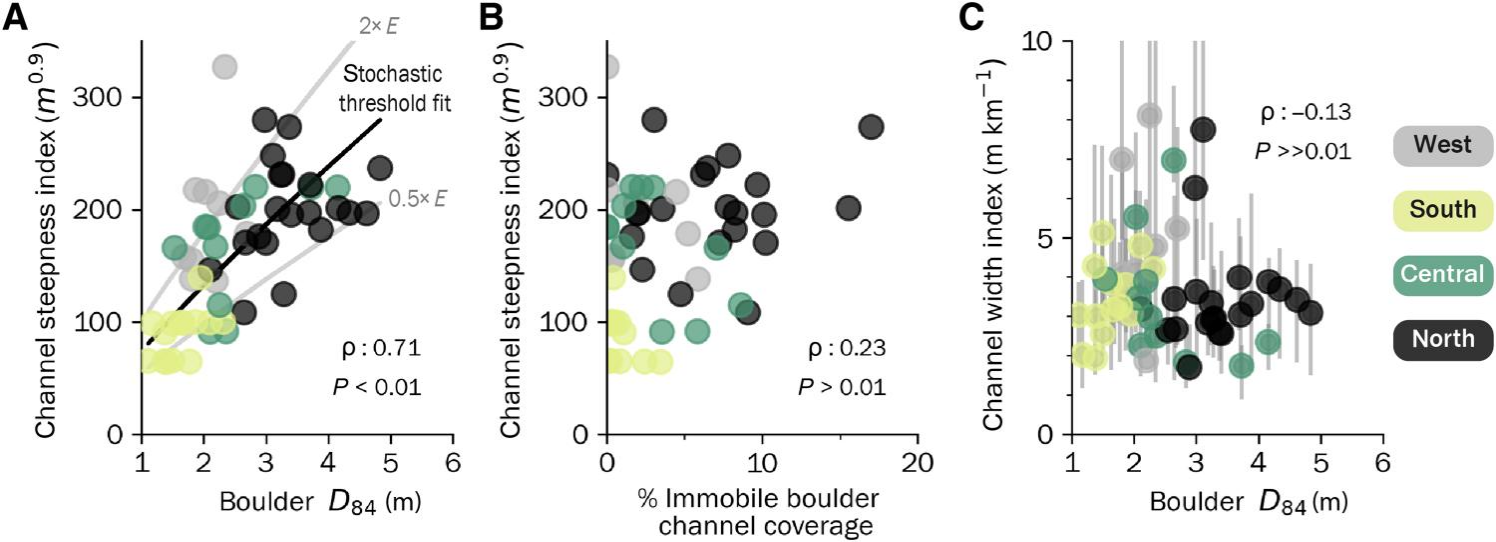
Reach-scale channel geometry versus boulder size and cover. (**A**) Channel steepness index versus *D*_84_ boulder diameter for all channel reaches, colorized by region. Black curve indicates expected relationship from river incision model where incision threshold scales with boulder size, which was fit to the data by varying the erodibility to minimize the root mean squared error between the model and predicted values. Gray curves indicate model results where erosion rates *E* are higher or lower by a factor of 2. (**B**) Channel steepness index versus areal immobile boulder coverage in channels. (**C**) Median channel width index versus *D*_84_ boulder diameter, with vertical error bars indicating sub-reach range of width measurements. In each panel, ρ indicates the Spearman rank correlation coefficient for all reaches.

At the reach scale, the median transport stage for the *D*_84_ boulder fraction is close to the threshold of motion across all regions but with substantial scatter among individual channel segments showing high-flow transport stages of 0.1 to 10 (Fig. 5A). This scatter in *D*_84_ transport stage appears to be driven by local variability in channel morphology with low transport stage associated with sections where boulders have low relative submergence (Fig. 5B) or where width-to-depth ratios are high (Fig. 5C and fig. S13). Additional scatter is also expected from uncertainties in defining boulder initial motion thresholds from reach-scale estimates of shear stress (*38*). At the regional scale, reach-scale channel steepness increases with *D*_84_ boulder size, showing similar sensitivity as expected by an incision model with a threshold that scales with boulder size (Fig. 6A). Reach-scale channel steepness does not significantly vary with immobile boulder cover (Fig. 6B), and boulder size shows no correlation with channel width index (Fig. 6C).

## DISCUSSION

### Rock strength influence of channel slope through boulder size and cover

We interpret the systematic pattern of increasing boulder size with increasing exhumational depth and metamorphic grade as a primary signature of rock strength in the Taiwan Central Range. This observed boulder size distribution will be set by the initial grain size distribution from hillslope mass wasting and modified through the rate of abrasion and residence time in the channel. Although the rock properties setting initial grain size may not be the same as those influencing the rate of breakdown by abrasion, we do not observe systematic variation in boulder size with drainage area across the study sites (fig. S10) and so assume that the observed grain size distribution is representative of landslide-derived sediment supply to the channel.

Previous work has demonstrated that the grain size distribution of coarse sediment delivered from steep hillslopes to channels can be dependent on bedrock fracture spacing (*15*, *16*), bedrock tensile strength (*14*), and grain size reduction during mass wasting (*39*). In the Taiwan Central Range, there is limited variation in bedrock compressive strength among major units (*40*), and we interpret the boulder size signal as corresponding to a decrease in the effective fracture density with increasing metamorphic grade. Low-grade rocks (Miocene slates) show multiple planes of weakness with prominent intersecting bedding, slaty cleavage, and fracture planes, whereas higher-grade rocks (i.e., Tananao complex) are overprinted by a secondary crenulation cleavage; these rocks tend to be more massive due to the increasing crystallinity associated with higher metamorphic grade (*9*). Additional lithologic variations within each major unit (slates versus sandstones in the Western Central Range; marbles versus schist in the Eastern Central Range) also appear to influence boulder size, but we lack the detailed boulder lithology data needed to quantify this effect.

Throughout the studied bedrock-bound channel reaches, the primary consequence of the variation in boulder size is that reach-scale channel steepness increases systematically with increasing boulder size (Fig. 6). Although all surveyed channel reaches are nearly continuously mantled in sediment, our estimates of immobile boulder cover fraction are typically less than 10%, with the remaining fraction often comprising a highly mobile gravel-cobble cover and boulders that are mobile at high flow (fig. S14). Our transport stage calculations indicate that most boulders are expected to be mobile or close to the threshold of mobility across the orogen, agreeing with observations of large boulder transport during high-flow events (*41*). The sensitivity of reach-scale channel steepness to boulder size additionally agrees with predictions from a bedrock incision model (*42*) where the incision threshold scales with boulder size (Fig. 4C) (*43*). We thus interpret reach-scale channel slopes in the Taiwan Central Range to be adjusted to mobilize the coarse boulder fraction much in the same way that gravel-bedded rivers are thought to adjust their geometry toward the threshold for bed mobilization (*44*).

A positive relationship between boulder cover fraction and slope might be expected where boulders are assumed to be immobile (*18*, *26*, *45*), due to bed armoring and the extraction of momentum from flows due to increased drag (*38*). Our boulder transport stage estimates (Fig. 5A) and observations of annual movement of 3- to 5-m boulders in the north (*41*) indicate that much of the boulder fraction may be mobilized during high flow events where bedrock incision occurs. We observe that immobile boulder cover increases regionally with exhumation depth and metamorphic grade but find no clear relationship at the reach scale between immobile cover and slope. We interpret this pattern to indicate that immobile boulder cover is low enough in all regions (Fig. 4E) to not promote substantial channel steepening (*46*).

### Local versus regional controls on channel width and depth

In contrast to the correlation between reach-scale channel steepness and boulder size, we find no systematic pattern in channel width or width-to-depth ratio, despite theoretical expectations. Channel width is highly variable within individual reaches (Fig. 2A), and often this variability is nearly that observed at the orogen scale (Fig. 3), spanning the full range of values typically found in bedrock and alluvial rivers (*47*, *48*). Similarly, at the reach scale, the channel width index shows no systematic variation with channel steepness index or boulder size (Fig. 6). This suggests that channel width is more sensitive to local- or reach-scale characteristics than to regional lithotectonic gradients.

We attribute the lack of systematic patterns in channel width to a suite of sub–reach-scale factors whose relative importance varies depending on setting. First, the orogen-scale lithostratigraphic units (Fig. 1) include finer-scale lithologic variations that are not captured by our analysis (i.e., bands of schist and marble alternating at the 10- to 100-m scale; Fig. 2A), and locally stronger rocks can result in channel narrowing. Second, channel width is sensitive to the presence and orientation of the fracture sets associated with dominant metamorphic fabrics (*49*, *50*), and channels can abruptly narrow when cutting across the strike of regional fabrics. Third, local inputs of coarse sediment from landslides can drive channel widening due to bed armoring in cases where boulders are infrequently mobile (*26*, *51*). Because we do not observe any relationship between channel width and grain size or cover in Taiwan, this factor is likely limited to segments with high immobile boulder cover. The relative strength of each of these factors varies from site to site, and they interact with each other. For example, landslides in weaker rocks can locally armor the bed and promote lateral incision, amplifying patterns in widening due to alternating strong and weak bedrock in channel banks. Together, these local controls on channel width appear to obscure systematic trends at the orogen scale.

### Implications for orogen-scale relationships between relief and erosion rate

Although our data indicate a connection between boulder size and reach-scale channel steepness that agrees with bedrock incision models (*42*, *52*, *53*) and field studies from other landscapes (*15*, *43*, *54*), it is challenging to reconcile this result with orogen-scale patterns of relief and erosion rate. Except for our southernmost site (Zhiben), our surveyed reaches come from the section of the Taiwan Central Range where the orogen-scale relief does not vary along the range, an observation that underlies the notion that the range is at or near topographic steady state (*27*, *35*, *55*). At the scale of watersheds that reach the topographic divide of the Central Range, bedrock channel steepness and erosion rates do not strongly vary despite the gradients in exhumation pathway and associated boulder size (Fig. 1).

To reconcile the lack of orogen-scale relief variation despite regional patterns in boulder size and reach-scale channel steepness, we consider a few possible explanations. First, our site selection could be biased toward steeper channels in the north. However, comparison of UAV-surveyed reaches with channel profiles from their larger encompassing watersheds indicates similar channel steepness values (fig. S15), such that we think a steepness bias is unlikely. In addition, large (>5 m) boulders are readily visible in satellite imagery, and we find no evidence that we have inadvertently undersampled boulders in nearby reaches of the southern and western regions. Second, because our UAV surveys focused on bedrock-bound channels, we do not account for mechanisms such as waterfall generation (*56*), alluviation from landslides (*57*), and debris flow processes in their contribution to landscape relief at the orogen scale. Although we presently lack data to evaluate in detail the partitioning of landscape relief in the Taiwan Central Range, there does not appear to be a systematic increase in waterfall or large landslide contribution toward the south or west. Rather, the magnitude of waterfall impact on landscape relief is argued to be highest in the north (*56*). Third, there is a scale mismatch between local channel incision rates and the watershed-averaged data that we use to interpret regional patterns in erosion rate. Agreement between nested samples and across different chronometers (Fig. 1) indicates that regional erosion rate patterns are robust, and we do not expect systematic biases to emerge at the reach scale. We suspect that, by isolating bedrock-bound channels, our analysis better reflects the local connection between boulder cover, channel geometry, and the resulting incision rate. Upscaling to the orogen scale requires incorporating at minimum partitioning the relief contribution of other channel processes.

Our interpretation that the steepness of bedrock rivers in the Taiwan Central Range is primarily determined by boulder size contrasts with prior work in Taiwan. In southern Taiwan, landslide flux increases with topographic relief (*57*, *58*), and bedrock rivers adjust to increases in sediment flux by both widening and steepening. However, southern Taiwan is underlain primarily by weakly metamorphosed slates that yield few large boulders [study site ZB is near the north of the study extent of (*57*)]. The lack of boulders points to channel slope and width being primarily adjusted to transport the high gravel and cobble flux (*57*). We hypothesize that the contrast from flux-dominated channels in the south to threshold-dominated channels in the north arises due to lithologic controls on sediment delivery and landslide style (*59*, *60*), with the stronger rocks of the Taiwan Central Range supporting steeper bedrock hillslopes along channel margins and coarser sediment in landslide debris. In the Taiwan Coastal Range, Lai *et al.* (*54*) found a link between the grain size of the mobile fraction (mainly gravel and cobble-sized sediment) and channel steepness but attributed the steepening to an increasing bedload flux that balanced a gradient in uplift rate. However, because of difficulties in directly measuring bedload flux, it is challenging to partition how much of the steepening of Coastal Range channels is a consequence of gradients in sediment flux versus gradients in incision thresholds. Although we were not able to quantify the size distribution of the gravel-cobble fraction from our sites in the Taiwan Central Range, this fraction did not appear to vary systematically across regions (fig. S13). We interpret the high-flow transport stages for boulders as indicating channel slopes in the Central Range are dictated by the thresholds for mobilizing the boulder fraction (Fig. 5), but we note that the sensitivity of bedload sediment flux to grain size makes it challenging to fully partition the relative roles of these two factors.

Our analysis provides a window into how boulder cover and channel geometry vary from the reach to the orogen scale. The increasing availability of high-resolution and large-scale channel survey data such as that presented here provides one possible link between high resolution, local observations, and large-scale regional models. The recent proliferation of models assessing the complexity of bedrock river incision through variable rock strength (*61*, *62*), variable grain size (*53*), stochastic sediment supply (*63*), dynamic and variable channel width variation (*64*, *65*), and mechanism of incision (*66*) provides insight into the outsized role of thresholds and variability in shaping landscapes. Coupling these models with high-resolution, and large-scale field data is crucial to characterize potential feedbacks between different processes at the landscape scale, and how those feedbacks may vary within individual landscapes, between landscapes, and over the course of a landscape evolving.

Overall, the systematic variation in boulder size with tectonic history across the Taiwan Central Range implies that a primary signature of rock strength in Taiwan landscapes is expressed through the size of sediment in channel systems. Although prior work has interpreted the steepness of bedrock rivers in Taiwan as limited by the amount of sediment delivered from landslides (*57*, *58*), our work implicates boulder size as a key control on steepness in the Taiwan Central Range. Consequently, tectonic history may be imprinted on the modern landscape through boulder size and cover, leading to feedbacks between tectonics and topography that emerge due to rock strength effects in addition to climatic factors.

## MATERIALS AND METHODS

### UAV surveys and 3D model generation

We quantified channel geometry and boulder cover using centimeter-scale UAV photogrammetric surveys conducted along 30 km of bedrock river corridors from nine sites across the Taiwan Central Range. UAV surveys were flown manually with a DJI Mavic Pro 40 to 80 m above the streambed at low-flow conditions in March 2018, March to April 2019, and January 2020. Each survey consisted of multiple UAV flights, where each flight overlapped by at least 50 m of streamwise distance. To capture the complex river corridor topography and minimize distortion, each flight consisted of a series of overlapping (>80% forward overlap and >60% side overlap) along-stream flight lines, combining nadir imagery with low-angle oblique (less than 10° off nadir) imagery facing upstream, downstream, and at the left and right banks (fig. S1) (*67*). Where the streambed was accessible, we laid identifiable ground control markers using survey flags and marking tape. The location of each ground control point was surveyed using an Eos Arrow Gold Bluetooth Global Navigation Satellite System (GNSS) receiver with horizontal and vertical uncertainties of 0.1 to 2 m and 1 to 5 m, respectively, due to limited satellite coverage in steep-walled canyons. We then used Agisoft Metashape 1.5.2 to build 3D dense point clouds and high-resolution (1 to 5 cm) orthoimages of each survey.

### Channel morphology and boulder cover measurement

We characterized channel geometry from the 3D dense point cloud models using CloudCompare (www.cloudcompare.org/) to map vegetation breaks and bedrock weathering contrasts that define high-water marks along channel banks (figs. S1 and S2). Because these contrasts are broadly consistent across our surveys, we assume that the high-water marks represent a similar flow recurrence interval across the Taiwan Central Range, representing typhoon flows (*11*), in the range of 5 to 10 years. Although direct measurements of high-water flow depths are rare, our interpretation of high flow depth from the Lushui gaging station (10.7 m measured at distance 1220 m on reach LSb_LSc) agrees with observations of scour and inundation up to 10 m above low flow from Typhoon Bilis in August 2000 (*68*). We mapped high-water marks on the left and right banks independently to (i) ensure that the high-water marks are representative of a similar flow, (ii) limit any bias in measurements, and (iii) ensure that there is no systematic distortion in the model. Channel width was measured as the distance between high-water marks, perpendicular to the flow direction (fig. S1). Flow depth was defined as the elevation difference between the high-water marks and the surveyed low-flow water line, which at the time of the surveys was typically less than 1 m and assumed to be negligible. To limit bias from variable sampling density, we resampled width and depth measurements to a streamwise interval of 20 m (fig. S3), and, at each reach, a single value for high-flow width and depth is reported. Because channel slopes were typically low and sensitive to the accuracy and distribution of ground control points, we used a regional 20-m digital elevation model (DEM) (*69*) to extract channel long profiles by hand and calculate local channel slope.

To characterize boulder cover, we used the high-resolution UAV orthomosaic imagery to manually measure the apparent intermediate axis (short axis in plan-view) of every boulder larger than 1 m in diameter. Errors on individual length measurements were calculated using known scale objects in surveys and were consistently less than 5 cm (fig. S4). Individual boulder area was calculated by squaring the intermediate axis measurement, and the fractional boulder coverage for each 20-m segment of channel was defined by the sum of individual boulder areas divided by the plan-view channel area (fig. S1) (*46*).

To calculate immobile boulder coverage (immobile boulder area divided by plan-view channel area), we estimated individual boulder mobility using the ratio of segment-scale high-flow shear stress, τ_hf_, and a critical shear stress for each boulder based on a uniform Shields criterion of $\tau_{c}^{*}=0.07$ as described in Eqs. 1 to 4 (figs. S5 and S6). Because of difficulties in measuring the full (non-boulder) grain size distribution, we do not account for hiding or protrusion effects. Because the boulders in our surveys occupy the coarse fraction of grain sizes, we expect that our approach is underestimating mobility (overestimating immobile boulder coverage). However, for the largest boulders, protrusion from the flow in shallow flows or bridging effects in narrow channels will lead to boulders being less mobile.

### Reach-scale calculations

To compile channel geometry and boulder cover measurements, we delineated each survey into distinct reaches defined by tributary junctions with at least 0.2-km^2^ contributing area, determined from the 20-m DEM (fig. S1). The resulting 55 reaches range in length from 150 to 1500 m, and, for each reach, we calculated the median channel width and depth; mean boulder cover; mean immobile boulder cover; and boulder *D*_50_, *D*_84_, and *D*_95_, which correspond to the 50th, 84th, and 95th percentile fractions of all boulders larger than 1 m in each reach, respectively. The normalized channel width index, *k_wn_* = *wA*^−*b*_ref_^, for each reach was calculated using the median drainage area for each reach, *A*, the median channel width, *w*, and a scaling exponent *b*_ref_ = 0.5 (*10*). The normalized channel steepness index, *k_sn_* = *SA*^θ_ref_^, for each reach was calculated using a reference concavity θ_ref_ = 0.45, and channel gradient *S* measured from the 20-m DEM (*37*). When calculating *S*, we removed the influence of bedrock waterfalls that affect 3 of the 55 reaches.

For each reach, we calculated the high-flow transport stage for the *D*_84_ boulder fraction defined by

$$\tau_{\mathrm{hf}}/\tau_{c84}=\frac{R_{h}S}{\tau_{c}^{*}RD_{84}}$$ (1)

where *R* is the submerged specific density of boulders, assumed to be 1.65, *S* is channel gradient, τ_hf_ is the high-flow shear stress, and $\tau_{c}^{*}$ is a Shields criterion for initial motion (*70*). The high-flow hydraulic radius, *R*_h_, was calculated assuming a rectangular channel

$$R_{h}=hw/(2h+w)$$ (2)

where *w* and *h* correspond to the median high-flow width and depth, respectively. We assume rectangular channels because the bedrock-bound channels in the Taiwan central range typically have steep banks and because inconsistent coverage of channel walls in the 3D point cloud precludes continuous direct measurement of cross-sectional channel geometry.

To calculate the critical Shields stress, $\tau_{c}^{*}$, we use the approach of Prancevic and Lamb (*71*), where $\tau_{c}^{*}$ depends on the friction factor, *C*_f_

$$\tau_{c}^{*}=0.19C_{f}^{0.34}$$ (3)

where *C*_f_ was determined using the variable power equation of Ferguson (*72*)

$$C_{f}^{-1/2}=\frac{U}{\sqrt{gR_{h}S}}=\frac{a_{1}a_{2}\frac{R_{h}}{D_{84}}}{{\left[a_{1}^{2}+a_{2}^{2}{\left(\frac{R_{h}}{D_{84}}\right)}^{5/3}\right]}^{1/2}}$$ (4)

where *U* is the mean flow velocity, *g* is gravitational acceleration, and *a*_1_ and *a*_2_ are constants assumed to be 6.5 and 2.5, respectively. The resulting reach-averaged values of $\tau_{c}^{*}$ vary from 0.05 to 0.12, with a mean of 0.07 (fig. S5). Because $\tau_{c}^{*}$ does not vary systematically across regions (figs. S5 and S6), we use a single value of $\tau_{c}^{*}$ for all calculations.

For simplicity, we assume that boulder mobility can be characterized using Eq. 1 with $\tau_{c}^{*}=0.07$ and focus on the *D*_84_ boulder fraction for comparison across sites. A more complete treatment of boulder transport is challenging with our data due to uncertainties about local-scale hydrodynamics, and we note two opposing effects that are not accounted for. First, as described above for immobile boulder coverage calculations, our approach may underestimate boulder mobility in cases where boulders comprise a small portion of the bed sediment cover due to protrusion and may overestimate boulder mobility in areas with high boulder cover due to hiding effects (*73*). Second, the extraction of momentum in the flow by immobile boulders may reduce the available shear stress for transporting boulders (*38*), and, in narrow reaches, there may be bridging effects across the channel (*74*) leading Eq. 1 to overestimate boulder transport. We also assume uniform flow with this approach, which differs from treatments of boulder transport during flash floods and outburst floods, for example (*75*, *76*).

### Erosion rate compilation

The regional summary of published erosion rates was based on detrital cosmogenic ^10^Be (*34*, *35*) and Zircon fission track (*35*) data. Cosmogenic measurements in the Western Central Range represent the closest sub-watershed from Derrieux *et al.* (*34*) to avoid integrating outside of the Central Range, whereas measurements on the Eastern Central Range are the bootstrap value for the entire basin.

### Stochastic threshold incision model

The stochastic threshold bedrock incision model of Lague *et al.* (*42*) combines a shear stress incision law with a threshold (*77*) and a stochastic distribution of flood events that follow an inverse gamma distribution

$$E=\int_{Qc}^{Q_{m}}I(Q)pdf(Q)dQ$$ (5)

$$I=KQ^{m}S^{n}-{\text{Ψ}}_{c}$$ (6)

$$\mathrm{pdf}(Q)=\frac{{\left(\bar{Q}k\right)}^{k+1}}{\text{Γ}(k+1)}\exp\left(-k\frac{\bar{Q}}{Q}\right)Q^{-(2+k)}$$ (7)

where *E* is the long-term bedrock incision rate and *I* is the instantaneous incision rate that scales with discharge, *Q*, slope, *S*, and a threshold term, Ψ*_c_*. *K* is a parameter that varies mainly with rock erodibility, and *m* and *n* are exponents. pdf(*Q*) is the probability distribution of discharge; Γ is the gamma function; *Q*_c_ and *Q*_m_ are the critical and maximum discharges, respectively, that define the limits of integration; *k* is a measure of discharge variability; and $\bar{Q}$ is the mean daily discharge, which can also be generalized as a mean runoff, $R_{b}=\bar{Q}/A$, where *A* is upstream drainage area.

For the convergent case where *E* is insensitive to *Q*_m_, Lague *et al.* (*42*) present an analytical solution that minimizes steady-state channel slope with respect to the normalized critical discharge, $Q_{c}^{*}=Q_{c}/\bar{Q}$ in the following expression

$$S^{n}=\frac{1}{K}\frac{\text{Γ}(k+1)k^{-\gamma }}{\text{Γ}(k+1-\gamma )}\text{Γ}{\left(k/Q_{c}^{*},k+1-\gamma \right)}^{-1}[E+\text{Γ}\left(k/Q_{c}^{*},k+1\right){\text{Ψ}}_{c}]{\bar{Q}}^{-m}$$ (8)

where γ is an exponent that controls the relationship between shear stress and at-a-station variations in discharge, and Γ(*a*, *x*) is the regularized gamma function

$$\text{Γ}(a,x)=\frac{1}{\text{Γ}(a)}\int_{0}^{x}y^{a-1}e^{-y}dy$$ (9)

For the case *n* = 1 and if the erosion threshold is assumed to correspond to the initial motion threshold for sediment covering the bed (*43*), Ψ*_c_* scales with sediment (boulder) diameter, *D*, as Ψ*_c_* ∝ *D*^1.5^. Recasting Eq. 8 in terms of a channel steepness index, *k_sn_* = *SA*^*m*/*n*^, and assuming *E*/Ψ*_c_* < 0.1 [i.e., threshold dominated regime of (*78*)] yields the following approximate scaling between steady-state channel steepness and boulder diameter

$$k_{sn}\propto D^{0.84}$$ (10)

We fit Eq. 8 through our data in Fig. 6A to show the expected scaling between boulder size and channel steepness assuming uniform climate and erosion rate across the study area, with the bedrock erodibility fit to minimize the root mean squared error between the model and predicted values. Although we do not see evidence for systematic variation in climate or erosion rate across the Central Range, and rock compressive strength does not appear to vary much among the exposed bedrock units (*40*), it is possible that the differences in rock material properties that determine boulder size (e.g., fracture density) may also affect bedrock erodibility. We assume that such variations will also primarily affect the magnitude of erosion thresholds and scale similarly to boulder size. Variations in boulder size are also likely to affect reach-scale flow resistance, which we do not account for in Eqs. 5 to 9. Together with variations in cross-sectional channel geometry and stochastic delivery of the sediment that determines *D*, these factors likely contribute to scatter in Fig. 6A and obscure trends within any individual region.

## Supplementary Materials

### This PDF file includes:

Figs. S1 to S15 Tables S1 and S2 Legends for data S1 to S4 References
