# Supplementary material for: Rock properties and sediment caliber govern bedrock river morphology across the Taiwan Central Range
Source: Sci Adv. 2023 Nov 15;9(46):eadg6794. doi: 10.1126/sciadv.adg6794 (PMC10651117; doi:10.1126/sciadv.adg6794)
Supplement: Supplementary file 1 — Figs. S1 to S15 Tables S1 and S2 Legends for data S1 to S4 References [file sciadv.adg6794_sm.pdf]

Science Advances
AAAS

Supplementary Materials for

Rock properties and sediment caliber govern bedrock river morphology across the Taiwan Central Range

Julia C. Carr et al.

Corresponding author: Julia C. Carr, jccarr@sfu.ca

Sci. Adv. **9**, eadg6794 (2023)
DOI: 10.1126/sciadv.adg6794

The PDF file includes:

Figs. S1 to S15
Tables S1 and S2
Legends for data S1 to S4
References

Other Supplementary Material for this manuscript includes the following:

Data S1 to S4

Supplementary Text

Table S1. Summary of study sites.

Site Name	Code	Basin	Name	Region	Lithology	E (^{10}Be) (mm yr^{-1})	E (ZFT) (mm yr^{-1})	Survey year
Lixing	LX	Lixing	力行	west	MI	0.9 ± 0.1 [34]	-	2019
Liqi	LQ	Liqi	栗栖	west	MI	1.8 ± 0.2 [34]	-	2019
Lakesi	LK	Lakesi	拉克斯	west	Ep	2.6 ± 0.4 [34]	-	2019
Tacijili	TC	Liwu	塔次基里	north	PM3	3.9 ± 1.1 [34]	2.7 [35]	2020
Tianxiang	TX	Liwu	天祥	north	PM3	3.9 ± 1.1 [34]	2.7 [35]	2020
Lushui	LS	Liwu	綠水	north	PM3 & PM2	3.9 ± 1.1 [34]	2.7 [35]	2020
Xipan	XP	Liwu	溪畔隧道	north	PM1	3.9 ± 1.1 [34]	2.7 [35]	2020
Lele	LL	Lele	樂樂	central	PM4 & Ep	-	4.2 [35]	2018
Xinwulu	XW	Xinwulu	新武呂	central	PM4	5.5 ± 0.9 [34]	3.8 [35]	2019
Cross Island Highway	CI	Xinwulu	南部橫貫公路	central	PM2	5.5 ± 0.9 [34]	3.8 [35]	2019
Zhiben	ZB	Zhiben	知本	south	MI	3.75 [35]	-	2020
Luye	LY	Luye	鹿野	south	Ep	2.8 ± 0.4 [34]	4 [35]	2020

Table S2. Parameters used for stochastic threshold incision model.

Variable	Value	Source
E	4 mm yr^{-1}	Figure 1
K	6.0×10^{-6} m$^{0.1}$s^{-1}	Fit to data in Figure 6A
R_b	2.19 m yr^{-1}	Lague et al., 2005 (*42*)
k	0.71	Lague et al., 2005 (*42*)
γ	0.75	DiBiase et al., 2011 (*78*)
m	0.45	Figure S15
n	1	Figure S15

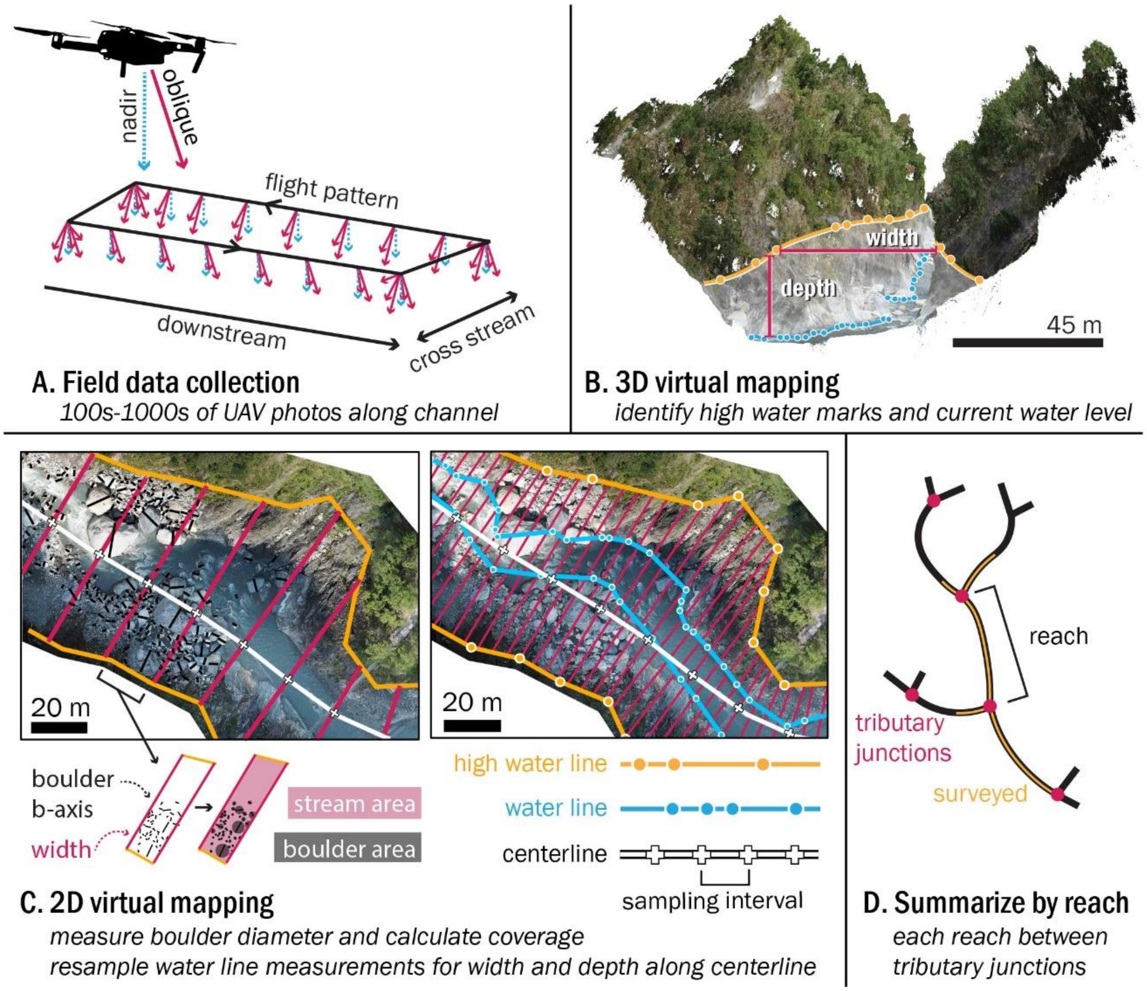

Figure S1. Overview of data collection workflow. (A) Photos are collected in the field from UAV surveys. Each reach is surveyed with nadir and oblique shots (looking upstream, downstream, and at both banks). (B) Identification of high-water marks (yellow) and water line marks (blue) from the colorized 3D point cloud model. (C) 2D virtual mapping using orthophotos. Boulder size and coverage is determined from cm-scale orthomosaic imagery. Width is measured perpendicular to the channel centerline between high water marks projected from 3D mapping (pink lines). Boulder size, cover, channel width, high water line marks, and water line measurements are extracted along the channel centerline. (D) Data are aggregated to the reach scale for comparison across the Taiwan Central Range study area. Each reach is defined between tributary junctions.

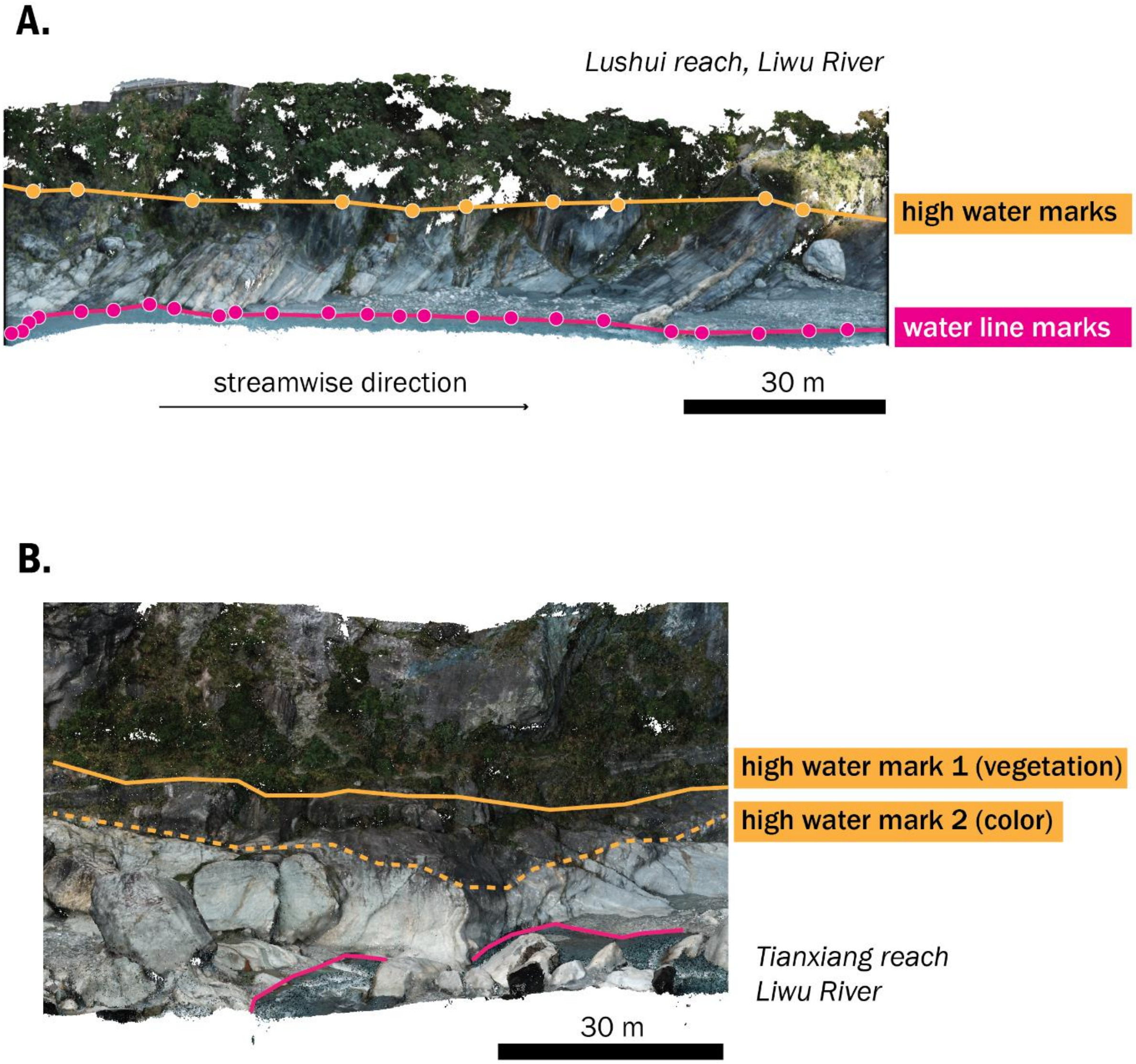

Figure S2. Example of high-water and waterline measurements from colorized 3D point clouds. (A) Raw high-water and water line marks from the Lushui reach. (B) Example of section with multiple high-water indicators, associated with a break in vegetation (solid line) and a break in bedrock weathering color (dashed line). In this case, the upper high-water mark 1 matched the high-water marks on the opposite bank and was used for the analysis.

.

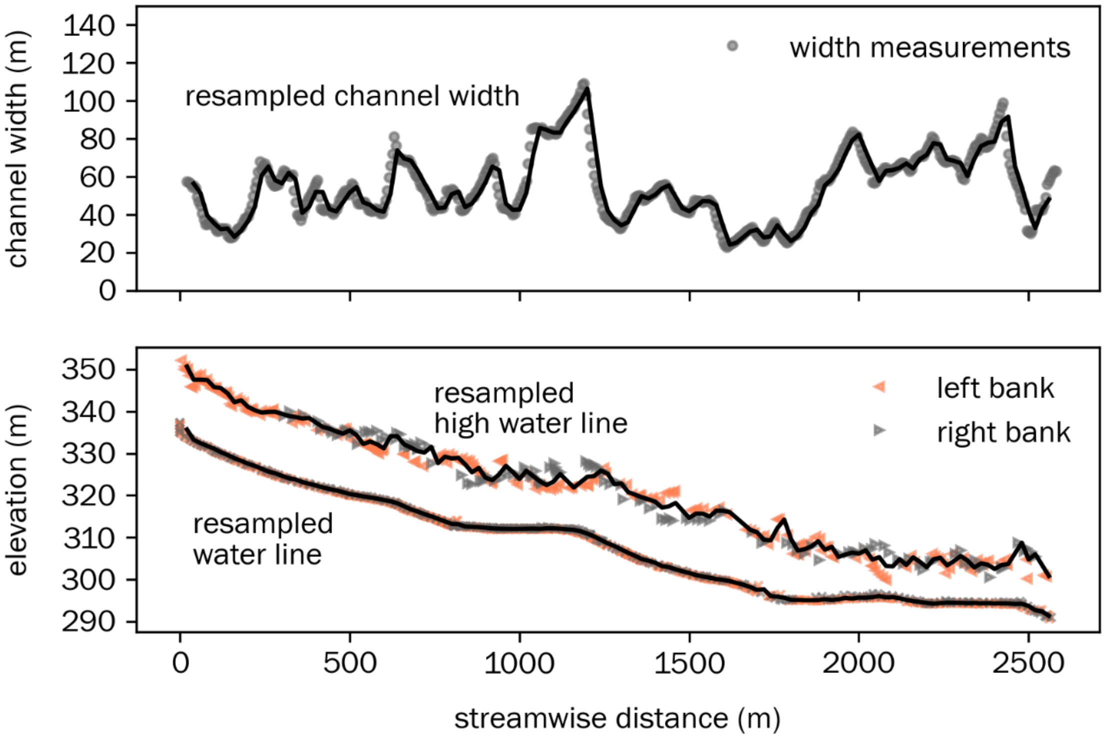

Figure S3. High-flow channel width and water-line measurements, showing effect of resampling (A) High flow channel width measurements from the LY reach showing raw data (points) and 20 m resampled data (line). (B) Left bank and right bank waterline and high-water line measurements, with 20 m resampled data in black.

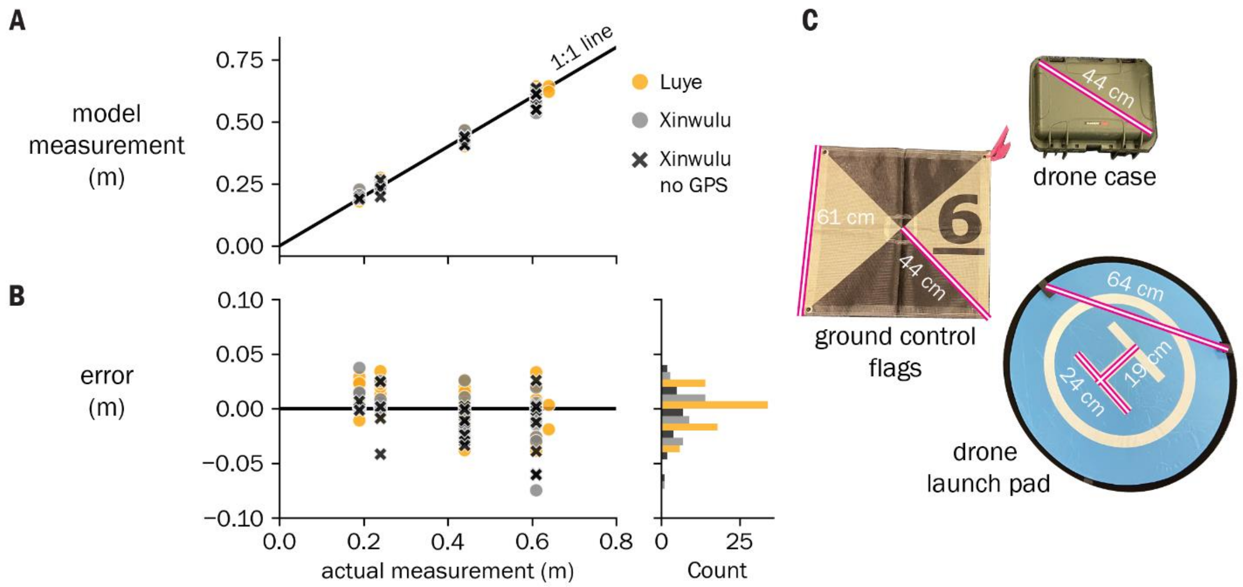

Figure S4. Overview of orthophoto measurement accuracy. (A) Orthophoto model measurements versus actual measurements of known objects from three surveys with and without ground control. (B) Distribution of errors showing nearly all errors less than 5 cm regardless of survey. (C) Reference items used for measurement.

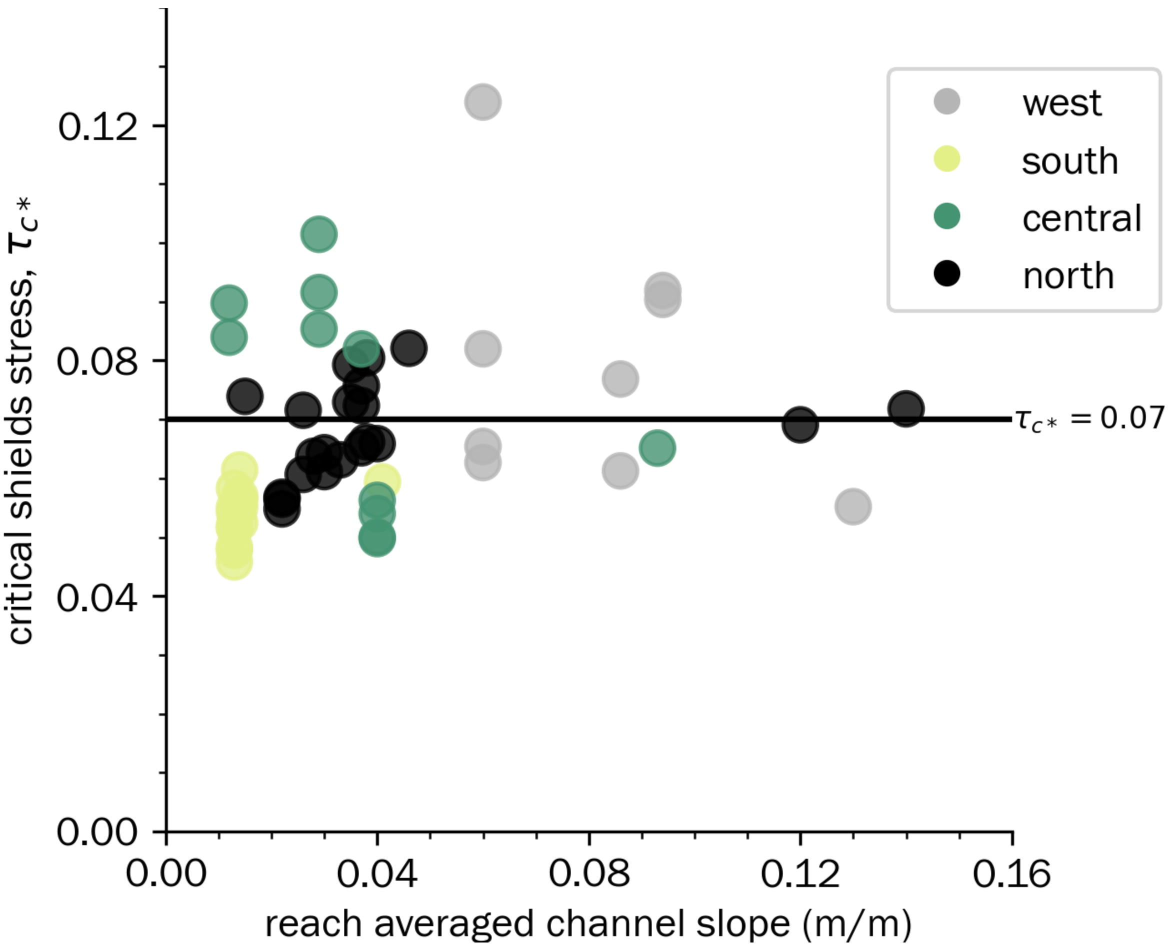

Fig. S5. Estimated critical Shields stress, τ_c^*, from Equation 3 for each reach in the orogen. Points are colored by region as in Figure 6, and the mean value $\boldsymbol{\tau_c^* = 0.07}$ is used for all calculations in the main text.

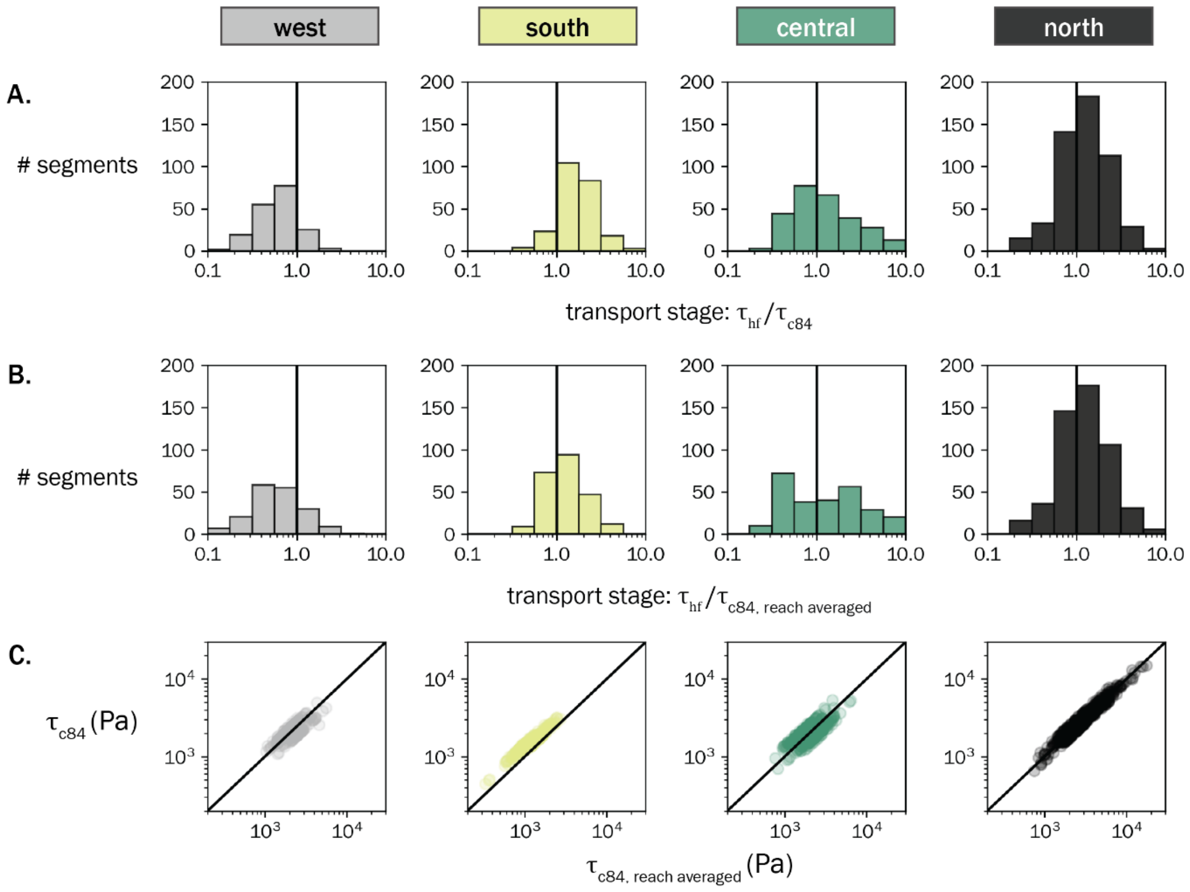

Figure S6. Sensitivity of transport stage calculations to orogen-averaged versus reach-averaged critical Shields stress assumption for each region. (A) Distribution of transport stages (τ_{hf}/τ_{c84}) for every 20-meter segment, assuming a uniform critical Shields stress ($\tau_c^* = 0.07$). (B) Distribution of transport stages (τ_{hf}/τ_{c84}) for every 20-meter segment, with τ_c^* calculated separately for each reach using Equations 3–4 (Figure S5). (C) Plots of the critical shear stress assuming uniform τ_c^* versus critical shear stress with reach-averaged τ_c^* ($\tau_{c84,\ reach\ averaged}$). Black line indicates 1:1 relationship. For simplicity, we assume a uniform value of $\tau_c^* = 0.07$ for all calculations in the main text (Figure S5).

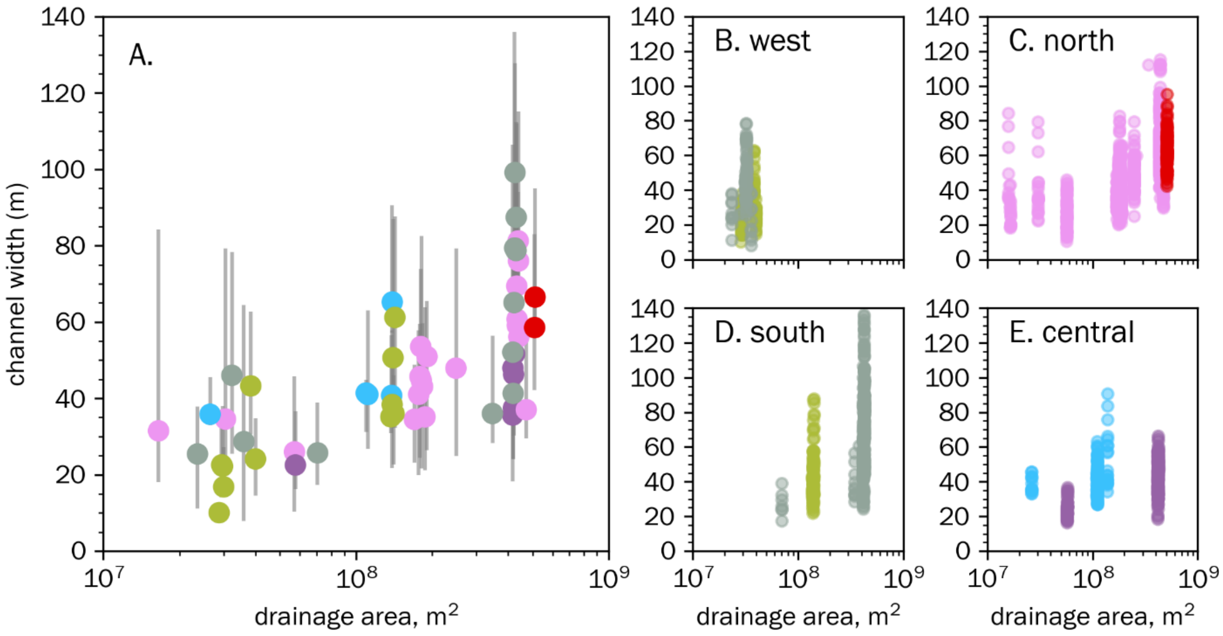

Figure S7. Channel width measurements plotted against upstream drainage area. (A) Median channel width (points) and range (bars) for each reach. Regional measurements plotted at the reach scale are shown for: (B) West, (C) North, (D) South, and (E) Central regions. All points are colored by lithology using the color scaling in Figure 1.

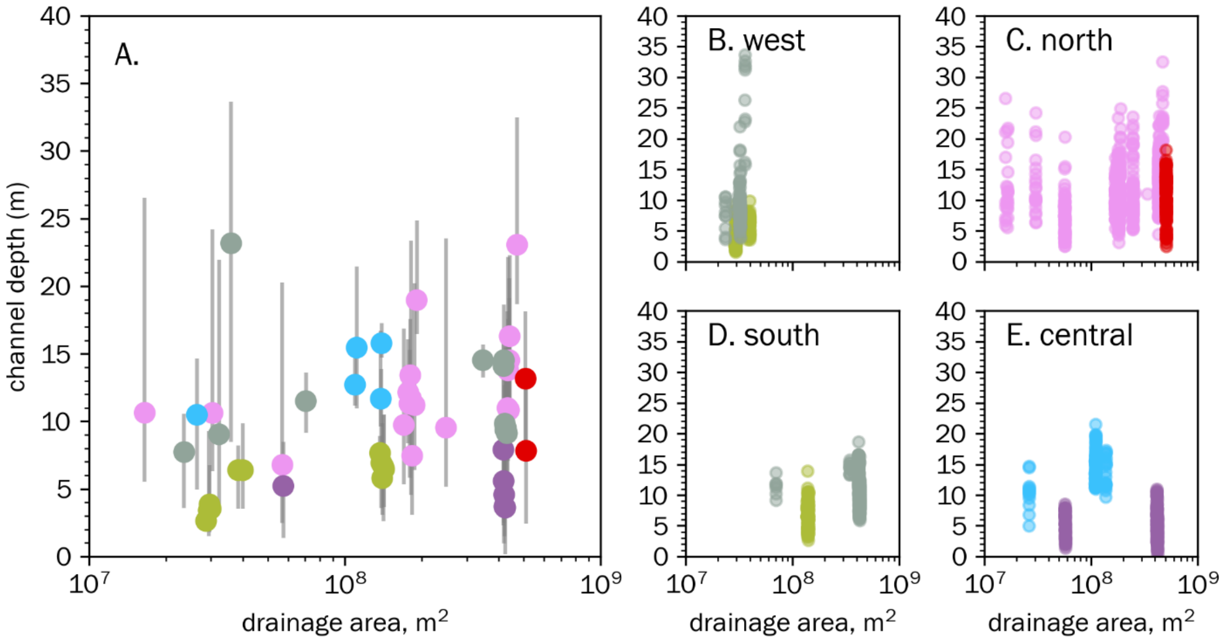

Figure S8. Channel depth measurements plotted against upstream drainage area. (A) Median channel depth (points) and range (bars) for each reach. Regional measurements plotted at the reach scale are shown for: (B) West, (C) North, (D) South, and (E) Central regions. All points are colored by lithology using the color scaling in Figure 1.

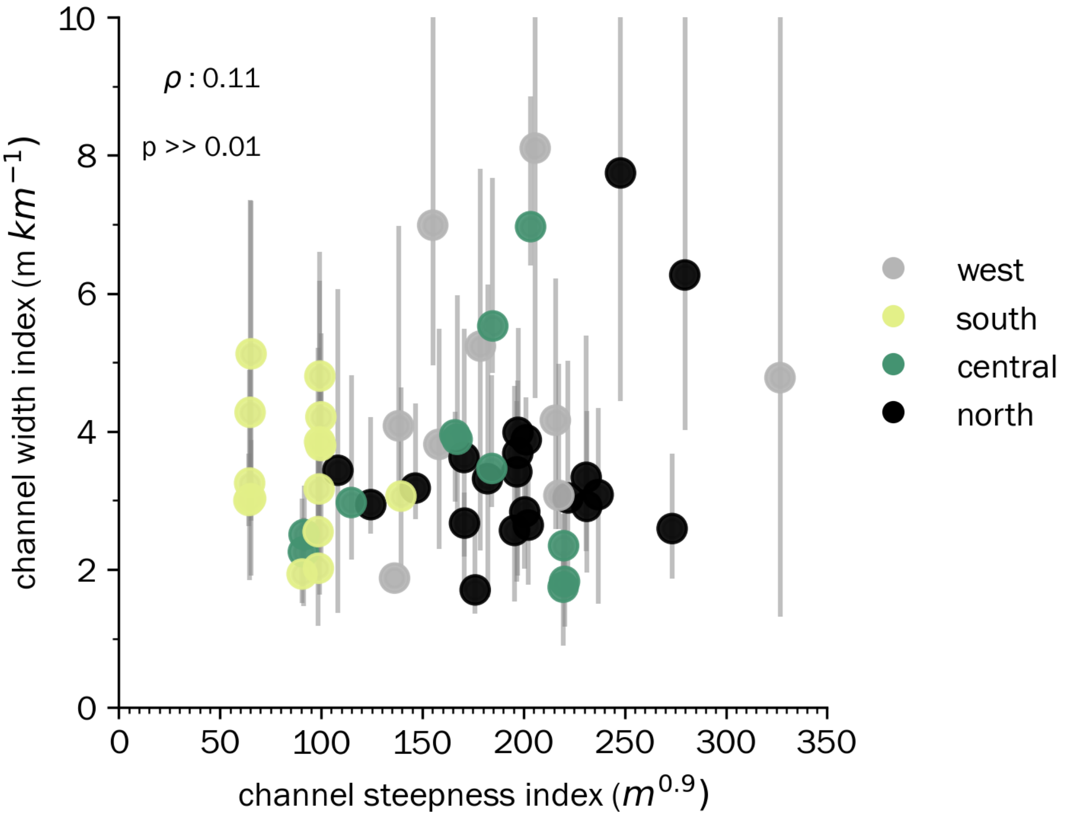

Figure S9. Median channel width index plotted against channel steepness index for each reach. Vertical error bars indicate the sub-reach scale range of channel width, and all points are colored by region.

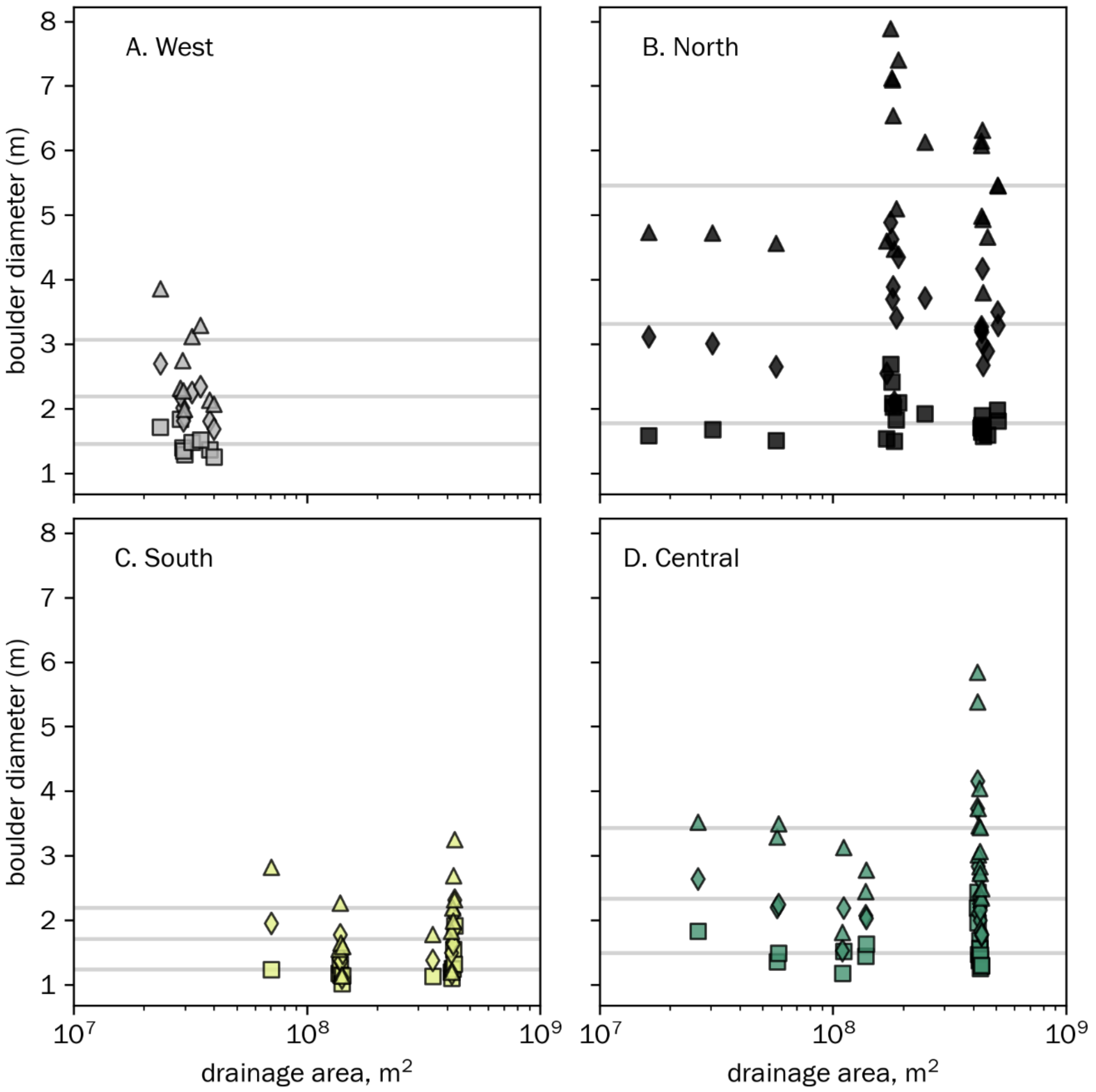

Figure S10. Reach-scale boulder size statistics plotted against drainage area by region. Grain size statistics (square = D_{50}, diamond = D_{84}, triangle = D_{95}) for each reach are plotted for: (A) West, (B) North, (C) South, and (D) Central regions. Composite regional statistics (Figure 4D) are shown as grey lines indicating D_{50}, D_{84}, and D_{95}.

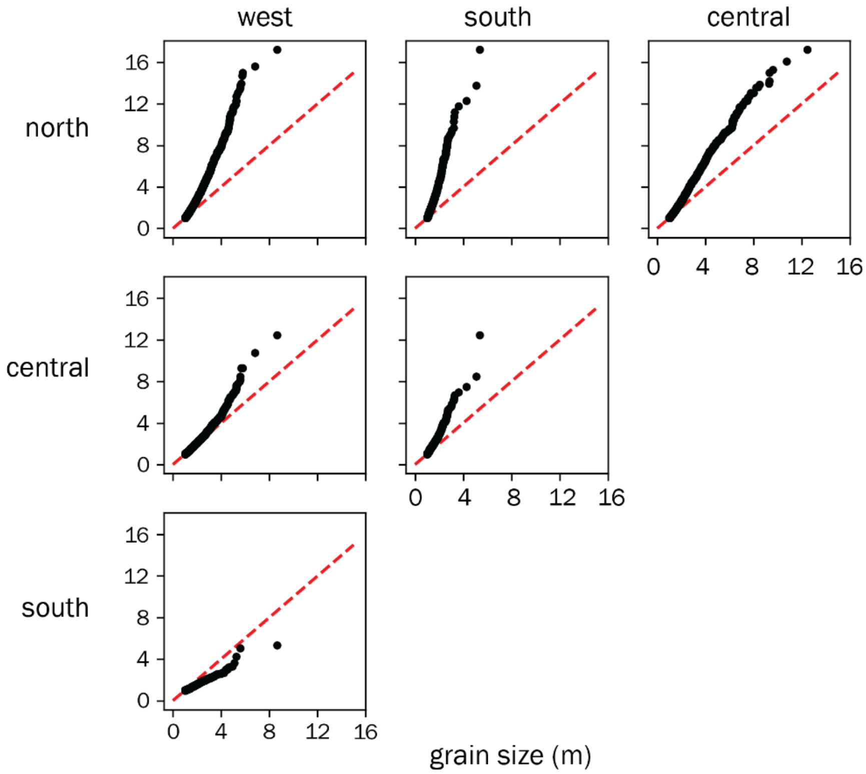

Figure S11. Quantile-Quantile plot for grain size distributions between each pair of regions. Each black dot represents a quantile of each dataset plotted against the same quantile from the other, with the red dashed line representing equivalence. The grain size distributions are different for all regions (two-sample Kolmogorov-Smirnov test for each pair, $p < 0.001$). The distributions from the West and the South regions are most similar, but the West has a right-skewed distribution.

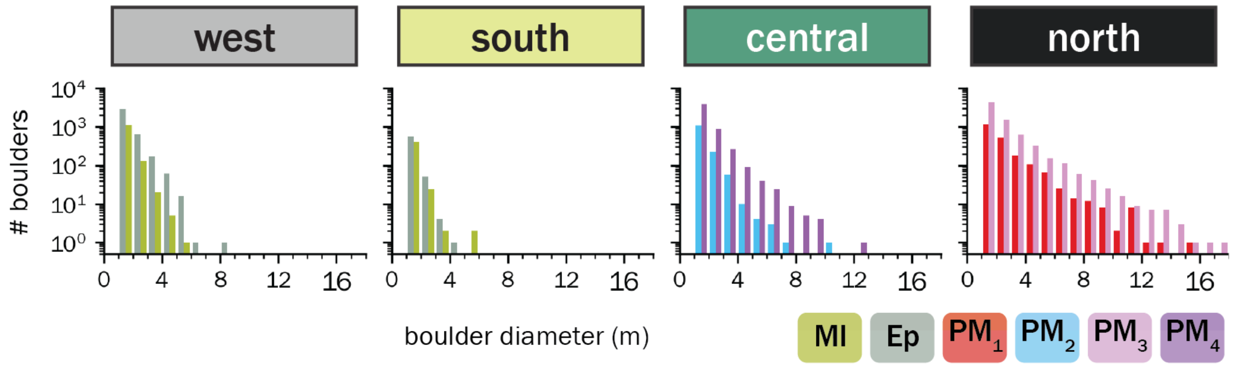

Figure S12. Grain size distributions for each region, colored by the primary lithostratigraphic unit for each reach (Figure 1). Although the absolute number of boulders is different between different lithologies within a region due to different aerial extent of each unit, the shape of the distribution is consistent within regions.

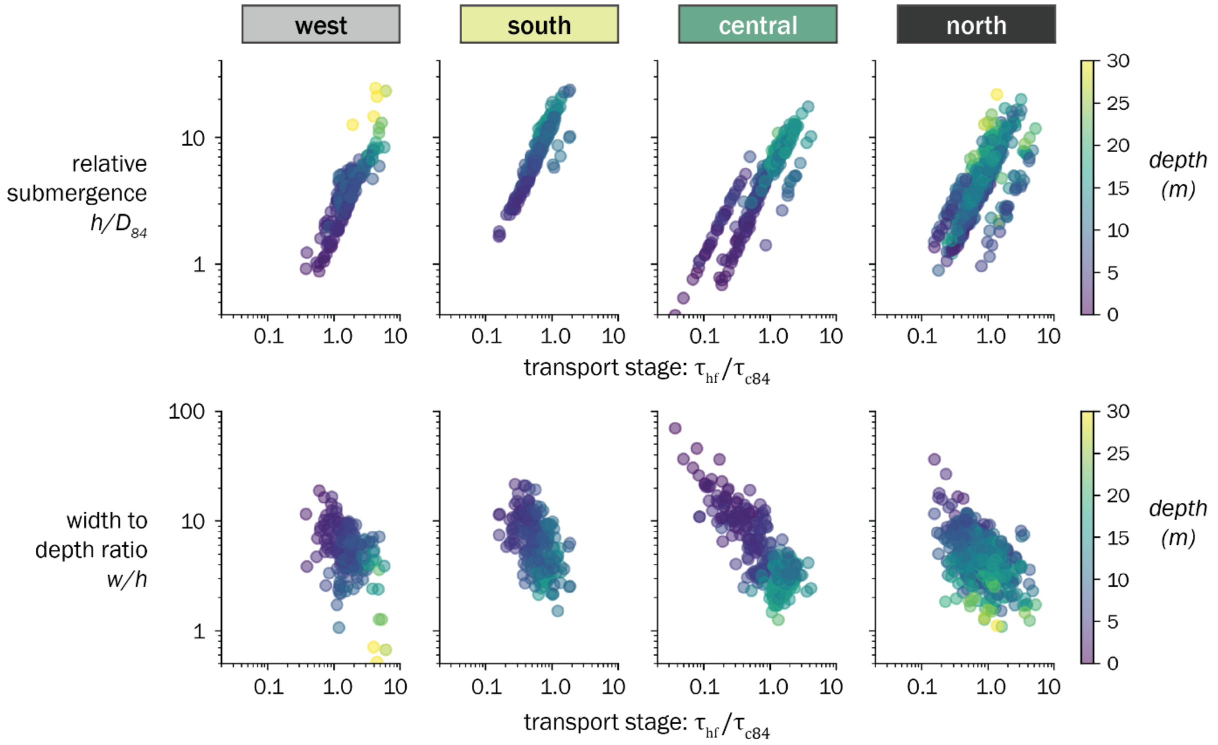

Figure S13. Relative submergence and width to depth ratio plotted against transport stage for each channel segment, showing that the range of transport stages is similar between regions, and that local variation is primarily driven by channel depth.

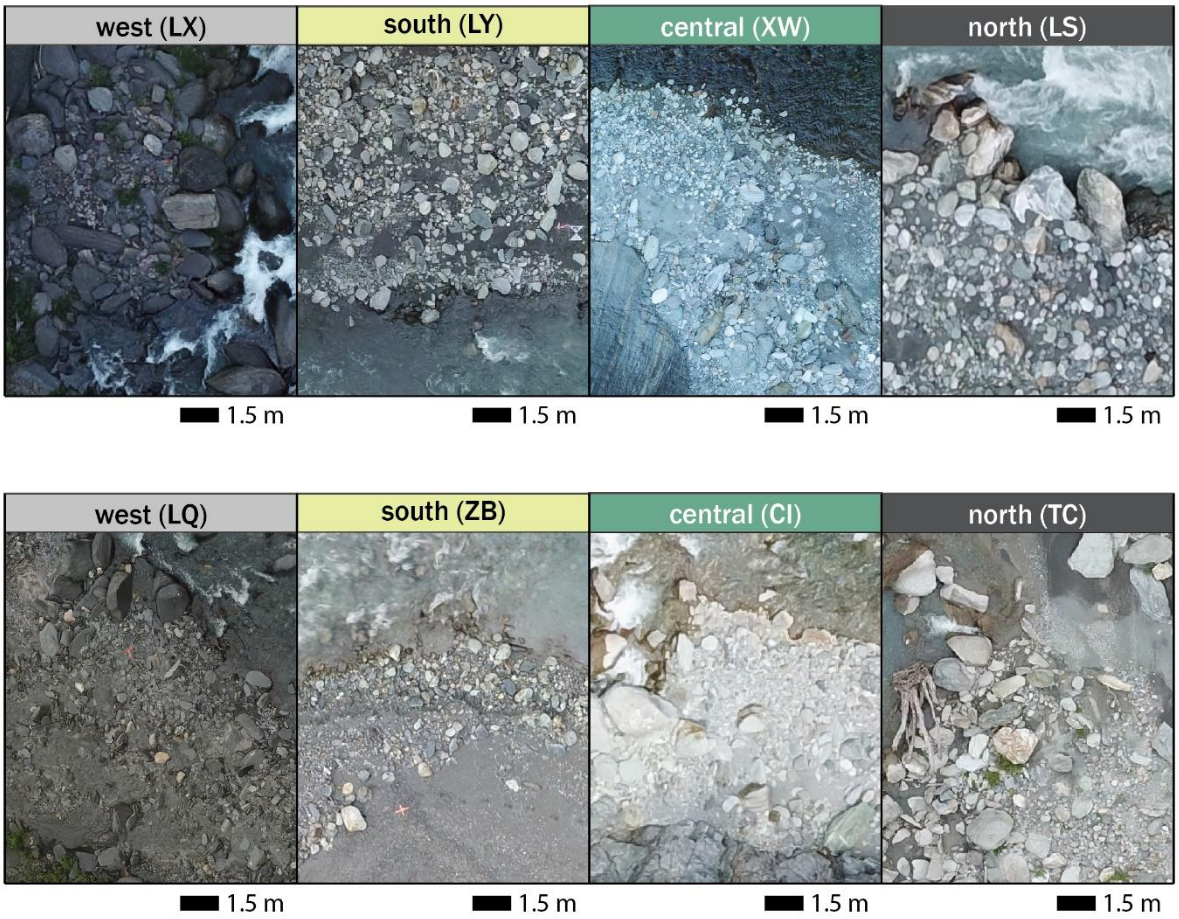

Figure S14. UAV orthophoto images of highly mobile gravel-cobble fraction by region. Each patch was selected along active bars to indicate the typical mobile sediment. Patches along bars typically have gravel-small boulder sized sediment, with D_{50} ~ 10–30 cm, though variability within sites is large.

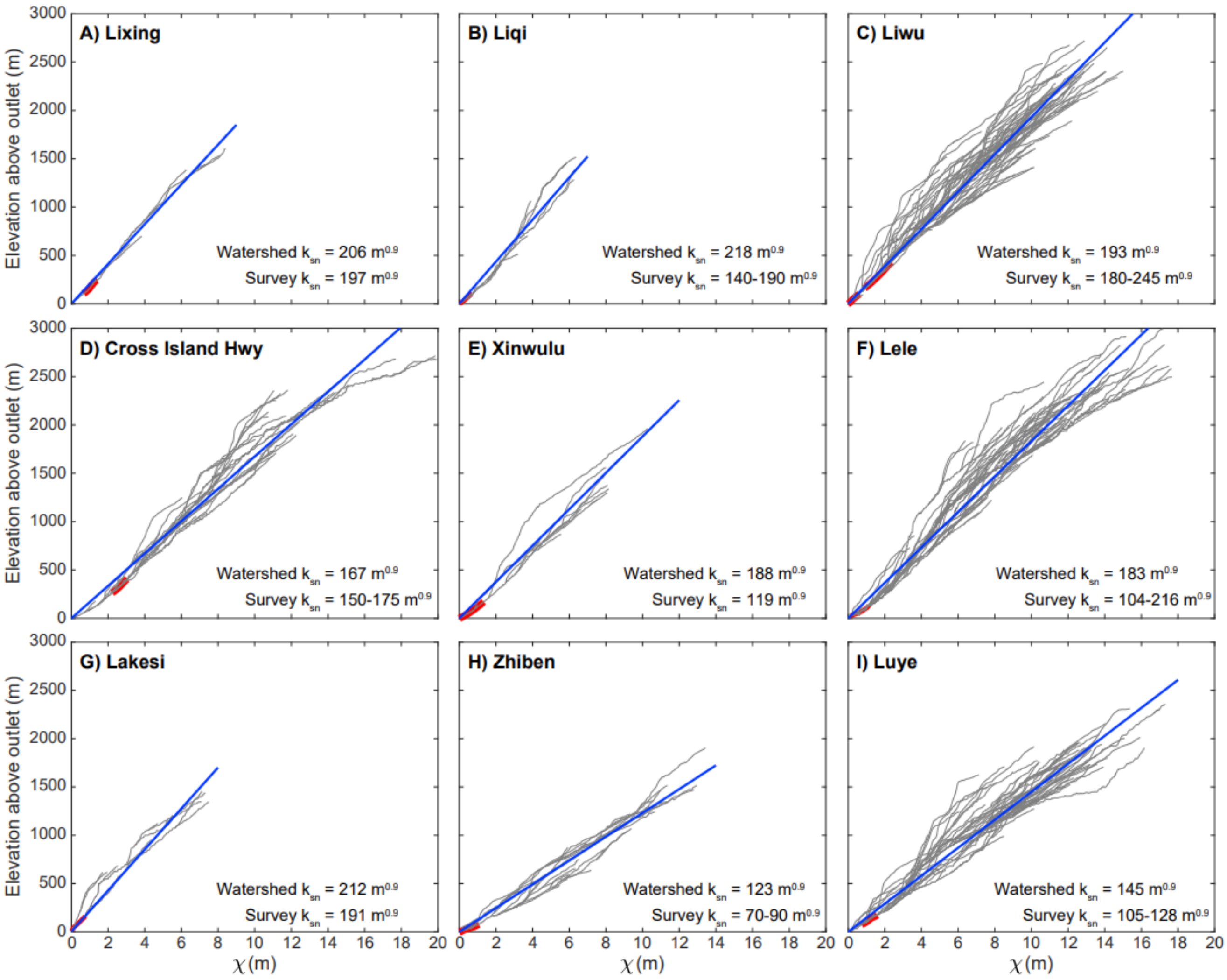

Figure S15. Context of surveyed reaches relative to watershed-scale channel steepness. Chi-elevation plots (*80*) of all channels with drainage area larger than 1 km^2 in each studied watershed, calculated using TopoToolbox (*81*), with reference concavity index of 0.45 and reference drainage area of 1 m^2. Blue lines indicate best-fit watershed-averaged channel steepness index (watershed k_{sn}), and red lines indicate location of individual UAV survey extents from this study, with the range of channel steepness within each region indicated (Survey k_{sn}).

Data S1. Channel Morphology Overview (separate file)

Extracted morphology and sediment metrics. File contains the upstream drainage area (km^2) channel width (m), channel depth (m), channel slope (m m^{-1}), width-to-depth ratio, channel plan-view area (m^2), total boulder area (m^2), immobile boulder area (m^2), and immobile boulder concentration (immobile boulder area divided by channel area), measured with streamwise distance (m) along the channel (1,492 measurements).

Data S2. Surveyed boulders (separate file)

Extracted grain size measurements with local context. File contains the boulder diameter (m) for each boulder (21,676 measurements), with context for the position along streamwise distance (m), upstream drainage area (km^2) channel width (m), channel depth (m), channel slope (m m^{-1}), and local high-flow shear stress (Pa). Shear stress is calculated as $\rho g R_h S$, where ρ is the density of water (1,000 kg m^{-3}), g is gravitational acceleration (9.8 m s^{-2}), R_h is the hydraulic radius (Equation 2), and S is the slope.

Data S3. Reach Summary (separate file)

Channel morphology and sediment metrics summarized for each reach. File contains the channel slope (m m^{-1}), normalized channel steepness (k_{sn}, $m^{0.9}$) median channel width (m), normalized channel width index (k_{wn}, m km^{-1}) median channel depth (m), 50th, 84th, 95th and 100th percentile boulder diameter (m), total plan-view channel area (m^2), total boulder area (m^2), and total immobile boulder area (m^2) for each of 55 reaches.

Data S4. Atlas of Surveyed Reaches (separate file)

Visual overview of surveyed reaches. PDF file shows a map with all tributary junctions used to define each reach (naming convention: *UpstreamNode_DownstreamNode*), and plotted channel width (dashed line), channel depth (solid line), boulder diameter, and boulder coverage (%) for each reach, organized by each river basin and region.

REFERENCES

1. J. T. Hack, Interpretation of erosional topography in humid temperate regions. *Am. J. Sci.* **258**, 80–97 (1960).

2. K. X. Whipple, B. J. Meade, Controls on the strength of coupling among climate, erosion, and deformation in two-sided, frictional orogenic wedges at steady state. *J. Geophys. Res. Earth Surf.* **109**, 10.1029/2003JF000019 (2004).

3. J. T. Perron, Climate and the pace of erosional landscape evolution. *Annu. Rev. Earth Planet. Sci.* **45**, 561–591 (2017).

4. G. E. Hilley, J. R. Arrowsmith, Geomorphic response to uplift along the Dragon’s Back pressure ridge, Carrizo Plain, California, *Geology* **36**, 367–370 (2008).

5. P. Molnar, R. S. Anderson, S. P. Anderson, Tectonics, fracturing of rock, and erosion. *J. Geophys. Res.* **112**, 10.1029/2005JF000433 (2007).

6. P. O. Koons, P. Upton, A. D. Barker, The influence of mechanical properties on the link between tectonic and topographic evolution. *Geomorphology* **137**, 168–180 (2012).

7. D. J. Miller, T. Dunne, Topographic perturbations of regional stresses and consequent bedrock fracturing. *J. Geophys. Res. Solid Earth.* **101**, 25523–25536 (1996).

8. J. S. Clair, S. Moon, W. S. Holbrook, J. T. Perron, C. S. Riebe, S. J. Martel, B. Carr, C. Harman, K. Singha, D. B. deB Richter, Geophysical imaging reveals topographic stress control of bedrock weathering. *Science* **350**, 534–538 (2015).

9. D. M. Fisher, S. D. Willett, E.-C. Yeh, M. B. Clark, Cleavage fronts and fans as reflections of orogen stress and kinematics in Taiwan. *Geology* **35**, 65–68 (2007).

10. D. Lague, The stream power river incision model: Evidence, theory and beyond. *Earth Surf. Process. Landf.* **39**, 38–61 (2014).

11. J. M. Turowski, N. Hovius, H. Meng-Long, D. Lague, C. Men-Chiang, Distribution of erosion across bedrock channels. *Earth Surf. Process. Landf.* **33**, 353–363 (2008).

12. L. S. Sklar, W. E. Dietrich, Sediment and rock strength controls on river incision into bedrock. *Geology* **29**, 1087–1090 (2001).

13. L. S. Sklar, C. S. Riebe, J. A. Marshall, J. Genetti, S. Leclere, C. L. Lukens, V. Merces, The problem of predicting the size distribution of sediment supplied by hillslopes to rivers. *Geomorphology* **277**, 31–49 (2017).

14. D. C. Roda-Boluda, M. D'Arcy, J. McDonald, A. C. Whittaker, Lithological controls on hillslope sediment supply: Insights from landslide activity and grain size distributions. *Earth Surf. Process. Landf.* **43**, 956–977 (2018).

15. A. B. Neely, R. A. DiBiase, Drainage area, bedrock fracture spacing, and weathering controls on landscape-scale patterns in surface sediment grain size. *J. Geophys. Res. Earth. Surf.* **125**, e2020JF005560 (2020).

16. J. P. Verdian, L. S. Sklar, C. S. Riebe, J. R. Moore, Sediment size on talus slopes correlates with fracture spacing on bedrock cliffs: Implications for predicting initial sediment size distributions on hillslopes. *Earth Surf. Dyn.* **9**, 1073–1090 (2021).

17. M. Attal, S. M. Mudd, M. D. Hurst, B. Weinman, K. Yoo, M. Naylor, Impact of change in erosion rate and landscape steepness on hillslope and fluvial sediments grain size in the Feather River basin (Sierra Nevada, California). *Earth Surf. Dyn.* **3**, 201–222 (2015).

18. C. M. Shobe, J. M. Turowski, R. Nativ, R. C. Glade, G. L. Bennett, B. Dini, The role of infrequently mobile boulders in modulating landscape evolution and geomorphic hazards. *Earth-Sci. Rev.* **220**, 103717 (2021).

19. J. G. Venditti, T. Li, E. Deal, E. Dingle, M. Church, Struggles with stream power: Connecting theory across scales. *Geomorphology* **366**, 106817 (2020).

20. K. X. Whipple, R. A. DiBiase, B. Crosby, J. P. L. Johnson, "Bedrock rivers," in *Treatise on Geomorphology* (Elsevier, 2022), pp. 865–903.

21. G. H. Allen, T. M. Pavelsky, Patterns of river width and surface area revealed by the satellite-derived North American River Width data set. *Geophys. Res. Lett.* **42**, 395–402 (2015).

22. T. M. Pavelsky, L. C. Smith, RivWidth: A software tool for the calculation of river widths from remotely sensed imagery. *IEEE Geosci. Remote Sens. Lett.* **5**, 70–73 (2008).

23. G. B. Fisher, B. Bookhagen, C. B. Amos, Channel planform geometry and slopes from freely available high-spatial resolution imagery and DEM fusion: Implications for channel width scalings, erosion proxies, and fluvial signatures in tectonically active landscapes. *Geomorphology* **194**, 46–56 (2013).

24. J. S. Eidmann, S. Gallen, New remote method to systematically extract bedrock channel width of small catchments across large spatial scales using high-resolution digital elevation models. *Earth Surf. Process. Landf.* **48**, 1470–1483 (2023).

25. K. L. Cook, An evaluation of the effectiveness of low-cost UAVs and structure from motion for geomorphic change detection. *Geomorphology* **278**, 195–208 (2017).

26. R. Nativ, J. M. Turowski, L. Goren, J. B. Laronne, J. B. H. Shyu, Influence of rarely mobile boulders on channel width and slope: Theory and field application. *J. Geophys. Res. Earth Surf.* **127**, e2021JF006537 (2022).

27. J. Suppe, Mechanics of mountain building and metamorphism in Taiwan. *Mem. Geol. Soc. China* **4**, 67–89 (1981).

28. S. Willett, C. Beaumont, P. Fullsack, Mechanical model for the tectonics of doubly vergent compressional orogens. *Geology* **21**, 371–374 (1993).

29. C. W. Fuller, S. D. Willett, D. M. Fisher, C. Y. Lu, A thermomechanical wedge model of Taiwan constrained by fission-track thermochronometry. *Tectonophysics* **425**, 1–24 (2006).

30. C. Conand, F. Mouthereau, J. Ganne, A. T. S. Lin, A. Lahfid, M. Daudet, L. Mesalles, S. Giletycz, M. Bonzani, Strain partitioning and exhumation in oblique Taiwan collision: Role of rift architecture and plate kinematics. *Tectonics* **39**, e2019TC005798 (2020).

31. Y. Zhang, C.-H. Tsai, K. Ustaszewski, N. Froitzheim, Y. Zhang, C.-H. Tsai, N. Froitzheim, K. Ustaszewski, The Yuli Belt in Taiwan: Part of the suture zone separating Eurasian and Philippine Sea plates. *Terr. Atmos. Ocean. Sci.* **31**, 415–435 (2020).

32. O. Beyssac, M. Simoes, J. P. Avouac, K. A. Farley, Y.-G. Chen, Y.-C. Chan, B. Goffé, Late Cenozoic metamorphic evolution and exhumation of Taiwan. *Tectonics* **26**, 10.1029/2006TC002064 (2007).

33. S. D. Willett, D. Fisher, C. Fuller, Y. En-Chao, L. Chia-Yu, Erosion rates and orogenic-wedge kinematics in Taiwan inferred from fission-track thermochronometry. *Geology* **31**, 945–948 (2003).

34. F. Derrieux, L. Siame, D. Bourles, R.-F. Chen, R. Braucher, L. Leanni, J.-C. Lee, H.-T. Chi, T. Byrne, How fast is the denudation of the Taiwan mountain belt? Perspectives from in situ cosmogenic ^{10}Be. *J. Asian Earth Sci.* **88**, 230–245 (2014).

35. M. G. Fellin, C.-Y. Chen, S. D. Willett, M. Christl, Y.-G. Chen, Erosion rates across space and timescales from a multi-proxy study of rivers of eastern Taiwan. *Glob. Planet Change* **157**, 174–193 (2017).

36. R. S. Pandey, Y.-A. Liou, Typhoon strength rising in the past four decades. *Weather Clim. Extremes* **36**, 100446 (2022).

37. C. Wobus, K. X. Whipple, E. Kirby, N. Snyder, J. Johnson, K. Spyropolou, B. Crosby, D. Sheehan, Tectonics from topography: Procedures, promise, and pitfalls. *Geol. Soc. Am. Spec. Pap.* **398**, 55–74 (2006).

38. E. M. Yager, J. W. Kirchner, W. E. Dietrich, Calculating bed load transport in steep boulder bed channels. *Water Resour. Res.* **43**, 10.1029/2006WR005432 (2007).

39. O. Marc, J. M. Turowski, P. Meunier, Controls on the grain size distribution of landslides in Taiwan: The influence of drop height, scar depth and bedrock strength. *Earth Surf. Dyn.* **9**, 995–1011 (2021).

40. S. J. Dadson, N. Hovius, H. Chen, W. B. Dade, M.-L. Hsieh, S. D. Willett, J.-C. Hu, M.-J. Horng, M.-C. Chen, C. P. Stark, D. Lague, J.-C. Lin, Links between erosion, runoff variability and seismicity in the Taiwan orogen. *Nature* **426**, 648–651 (2003).

41. J. Carr, Rock strength and sediment controls on bedrock river incision in the Taiwan Central Range, PhD thesis, Pennsylvania State University, University Park, PA (2022).

42. D. Lague, N. Hovius, P. Davy, Discharge, discharge variability, and the bedrock channel profile. *J. Geophys. Res. Earth Surf.* **110**, 10.1029/2004JF000259 (2005).

43. R. A. DiBiase, M. W. Rossi, A. B. Neely, Fracture density and grain size controls on the relief structure of bedrock landscapes. *Geology*, **46**, 399–402 (2018).

44. C. B. Phillips, D. J. Jerolmack, Self-organization of river channels as a critical filter on climate signals. *Science* **352**, 694–697 (2016).

45. E. A. Thaler, M. D. Covington, The influence of sandstone caprock material on bedrock channel steepness within a tectonically passive setting: Buffalo National River Basin, Arkansas, USA. *J. Geophys. Res. Earth Surf.* **121**, 1635–1650 (2016).

46. C. M. Shobe, G. L. Bennett, G. E. Tucker, K. Roback, S. R. Miller, J. J. Roering, Boulders as a lithologic control on river and landscape response to tectonic forcing at the Mendocino triple junction. *GSA Bulletin* **133**, 647–662 (2020).

47. N. J. Finnegan, G. Roe, D. R. Montgomery, B. Hallet, Controls on the channel width of rivers: Implications for modeling fluvial incision of bedrock. *Geology* **33**, 229 (2005).

48. B. J. Yanites, G. E. Tucker, K. J. Mueller, Y.-G. Chen, T. Wilcox, S.-Y. Huang, K.-W. Shi, Incision and channel morphology across active structures along the Peikang River, central Taiwan: Implications for the importance of channel width. *Geol. Soc. Am. Bull.* **122**, 1192–1208 (2010).

49. J. Ehlen, E. Wohl, Joints and landform evolution in bedrock canyons. *Transactions, Japanese Geomorphological Union.* **23**, 237–255 (2002).

50. D. N. Scott, E. E. Wohl, Bedrock fracture influences on geomorphic process and form across process domains and scales. *Earth Surf. Process. Landf.* **44**, 27–45 (2019).

51. T. Li, T. K. Fuller, L. S. Sklar, K. B. Gran, J. G. Venditti, A mechanistic model for lateral erosion of bedrock channel banks by bedload particle impacts. *J. Geophys. Res. Earth Surf.* **125**, e2019JF005509 (2020).

52. C. M. Shobe, G. E. Tucker, R. S. Anderson, Hillslope-derived blocks retard river incision. *Geophys. Res. Lett.* **43**, 5070–5078 (2016).

53. C. M. Shobe, G. E. Tucker, M. W. Rossi, Variable-threshold behavior in rivers arising from hillslope-derived blocks. *J. Geophys. Res. Earth Surf.* **123**, 1931–1957 (2018).

54. L. S.-H. Lai, J. J. Roering, N. J. Finnegan, R. J. Dorsey, J.-Y. Yen, Coarse sediment supply sets the slope of bedrock channels in rapidly uplifting terrain: Field and topographic evidence from eastern Taiwan. *Earth Surf. Process. Landf.* **46**, 2671–2689 (2021).

55. S. D. Willett, M. T. Brandon, On steady states in mountain belts. *Geology* **30**, 175–178 (2002).

56. C. W. Wobus, B. T. Crosby, K. X. Whipple, Hanging valleys in fluvial systems: Controls on occurrence and implications for landscape evolution. *J. Geophys. Res. Earth Surf.* **111**, 10.1029/20050406 (2006).

57. B. J. Yanites, N. A. Mitchell, J. C. Bregy, G. A. Carlson, K. Cataldo, M. Holahan, G. H. Johnston, A. Nelson, J. Valenza, M. Wanker, Landslides control the spatial and temporal variation of channel width in southern Taiwan: Implications for landscape evolution and cascading hazards in steep, tectonically active landscapes. *Earth Surf. Process. Landf.* **43**, 1782–1797 (2018).

58. C. DeLisle, B. Yanites, C.-Y Chen, J. Shyu, T. M. Rittenour, Extreme event-driven sediment aggradation and erosional buffering along a tectonic gradient in southern Taiwan. *Geology* **50**, 16–20 (2022).

59. C. W. Lin, W. S. Chang, S. H. Liu, T. T. Tsai, S. P. Lee, Y. C. Tsang, C. L. Shieh, C. M. Tseng, Landslides triggered by the 7 August 2009 Typhoon Morakot in southern Taiwan. *Eng. Geol.* **123**, 3–12 (2011).

60. C. Huang, T. B. Byrne, W. B. Ouimet, C.-W. Lin, J.-C. Hu, L.-Y. Fei, Y.-B. Wang, Tectonic foliations and the distribution of landslides in the southern Central Range, Taiwan, *Tectonophysics* **692**, 203–212 (2016).

61. A. M. Forte, B. J. Yanites, K. X. Whipple, Complexities of landscape evolution during incision through layered stratigraphy with contrasts in rock strength. *Earth Surf. Process. Landf.* **41**, 1736–1757 (2016).

62. N. A. Mitchell, B. J. Yanites, Bedrock river erosion through dipping layered rocks: Quantifying erodibility through kinematic wave speed. *Earth Surf. Dyn.* **9**, 723–753 (2021).

63. B. Campforts, C. M. Shobe, I. Overeem, G. E. Tucker, The art of landslides: How stochastic mass wasting shapes topography and influences landscape dynamics. *J. Geophys. Res. Earth Surf.* **127** (2022).

64. J. M. Turowski, Upscaling sediment-flux-dependent fluvial bedrock incision to long timescales. *J. Geophys. Res. Earth Surf.* **126** e2020JF005880 (2021).

65. B. J. Yanites, The dynamics of channel slope, width, and sediment in actively eroding bedrock river systems. *J. Geophys. Res. Earth Surf.* **123**, 1504–1527 (2018).

66. P. Chatanantavet, G. Parker, Physically based modeling of bedrock incision by abrasion, plucking, and macroabrasion. *J. Geophys. Res.* **114** 10.1029/2008JF001044 (2009).

67. M. R. James, S. Robson, Mitigating systematic error in topographic models derived from UAV and ground-based image networks. *Earth Surf. Process. Landf.* **39**, 1413–1420 (2014).

68. K. Hartshorn, N. Hovius, W. B. Dade, R. L. Slingerland, Climate-driven bedrock incision in an active mountain belt *Science* **297**, 2036–2038 (2002).

69. Ministry of Interior Taiwan, 內政部20公尺網格數值地形模型資料 (2016); https://data.gov.tw/dataset/35430 (Accessed 25 September 2019).

70. J. M. Turowski, E. M. Yager, A. Badoux, D. Rickenmann, P. Molnar, The impact of exceptional events on erosion, bedload transport and channel stability in a step-pool channel. *Earth Surf. Process. Landf.* **34**, 1661–1673 (2009).

71. J. P. Prancevic, M. P. Lamb, Unraveling bed slope from relative roughness in initial sediment motion. *J. Geophys. Res. Earth Surf.* **120**, 474–489 (2015).

72. R. Ferguson, Flow resistance equations for gravel- and boulder-bed streams. *Water Resour. Res.* **43** 10.1029/2006WR005422 (2007).

73. P. A. Carling, Threshold of coarse sediment transport in broad and narrow natural streams. *Earth Surf. Process. Landf.* **8**, 1–18 (1983).

74. A. Zimmermann, M. Church, M. A. Hassan, Step-pool stability: Testing the jammed state hypothesis. *J. Geophys. Res. Earth Surf.* **115**, 2008 (2010).

75. M. L. Huber, M. Lupker, S. F. Gallen, M. Christl, A. P. Gajurel, Timing of exotic, far-traveled boulder emplacement and paleo-outburst flooding in the central Himalayas. *Earth Surf. Dyn.* **8**, 769–787 (2020).

76. J. Alexander, M. J. Cooker, Moving boulders in flash floods and estimating flow conditions using boulders in ancient deposits. *Sedimentology* **63**, 1582–1595 (2016).

77. A. D. Howard, G. Kerby, Channel changes in badlands. *GSA Bulletin* **94**, 739–752 (1983).

78. R. A. DiBiase, K. X. Whipple, The influence of erosion thresholds and runoff variability on the relationships among topography, climate, and erosion rate. *J. Geophys. Res.* **116** 10.1029/2011JF002095 (2011).

79. J. C. Carr, R. A. DiBiase, E. C. Yeh, D. M. Fisher, E. Kirby, Bedrock River channels of the Taiwan Central Range, 2018-2020. *Sci. Adv.* 10.5069/G9154F8F (2023).

80. J. T. Perron, L. Royden, An integral approach to bedrock river profile analysis. *Earth Surf. Process. Landf.* **38**, 570–576 (2013).

81. W. Schwanghart, D. Scherler, Short Communication: TopoToolbox 2 – MATLAB-based software for topographic analysis and modeling in Earth surface sciences. *Earth Surf. Dyn.* **2**, 1–7 (2014).
