# Supplementary material for: Rock properties and sediment caliber govern bedrock river morphology across the Taiwan Central Range
Source: Sci Adv. 2023 Nov 15;9(46):eadg6794. doi: 10.1126/sciadv.adg6794 (PMC10651117; doi:10.1126/sciadv.adg6794)
Supplement: Supplementary file 2 — Data S1 to S4 [file sciadv.adg6794_data_s1_to_s4.zip › adg6794_Data_S4.pdf]

Atlas of Surveyed Reaches for

Rock strength and sediment caliber govern bedrock river morphology across the Taiwan Central Range

Julia C. Carr* *et al.*

*Corresponding author. Email: jccarr@sfu.ca

This file is intended to be used as a visualization for the dataset, expanding on the visual in Figure 2.

Each site has a map, showing the tributary junctions used to define each reach. The reaches are named with the naming convention *UpstreamNode_DownstreamNode*.

Each reach has a plot showing channel width (dashed line, *m*), channel depth (solid line, *m*), boulder diameter (*m*), boulder coverage for boulders >1m (%), and immobile boulder coverage for each reach, separated for each river basin and region.

Contents

Region: West 2
- Site: Lixing 2
- Site: Liqi 4
- Site: Lakesi 7

Region: South 10
- Site: Zhiben 10
- Site: Luye 14

Region: Central 20
- Site: Cross Island Highway, Xinwulu basin 20
- Site: Xinwulu, Xinwulu basin 24
- Site: Lele 26

Region: North 30
- Site: Tacijili (TC), Tianxiang (TX), Lushui (LS), Liwu basin 30
- Site: Xipan (XP), Liwu basin 41

Region: West

Site: Lixing

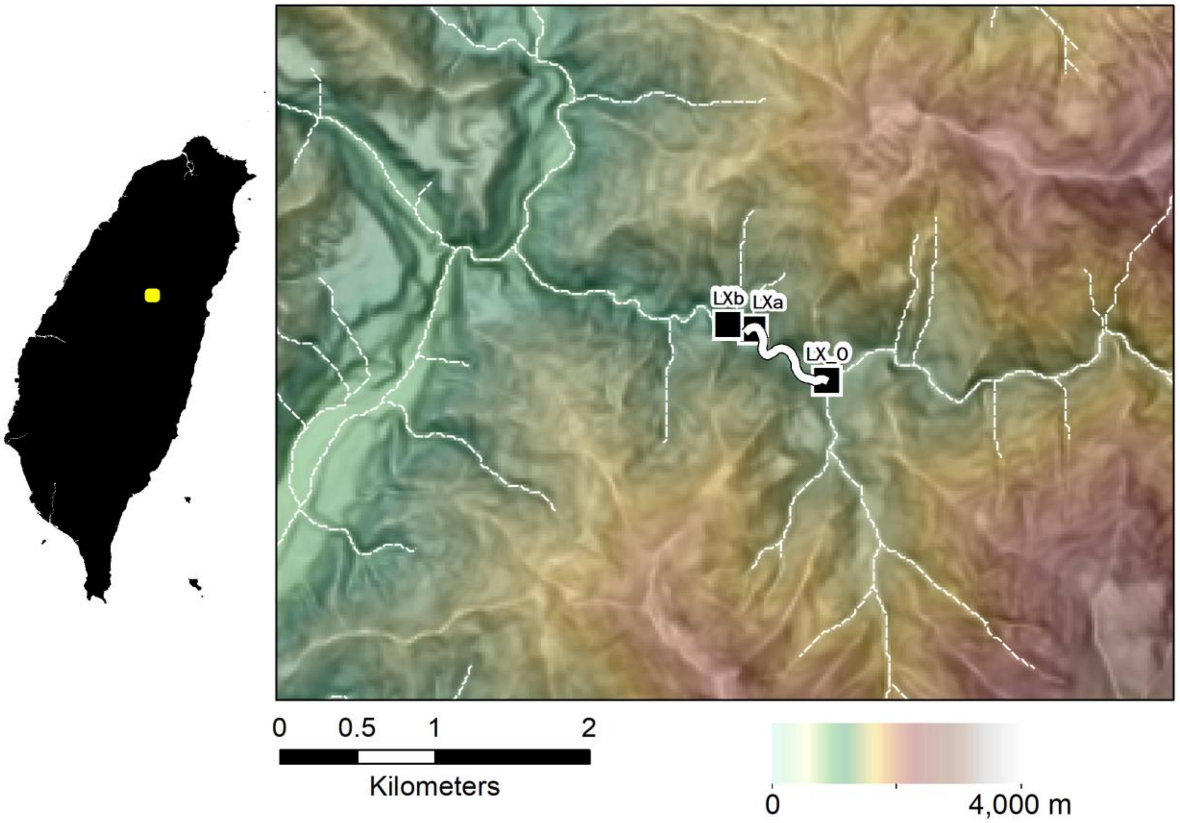

Reach: LX_0_LXa | Survey: LX | Watershed: Lixing | Slope: 0.09

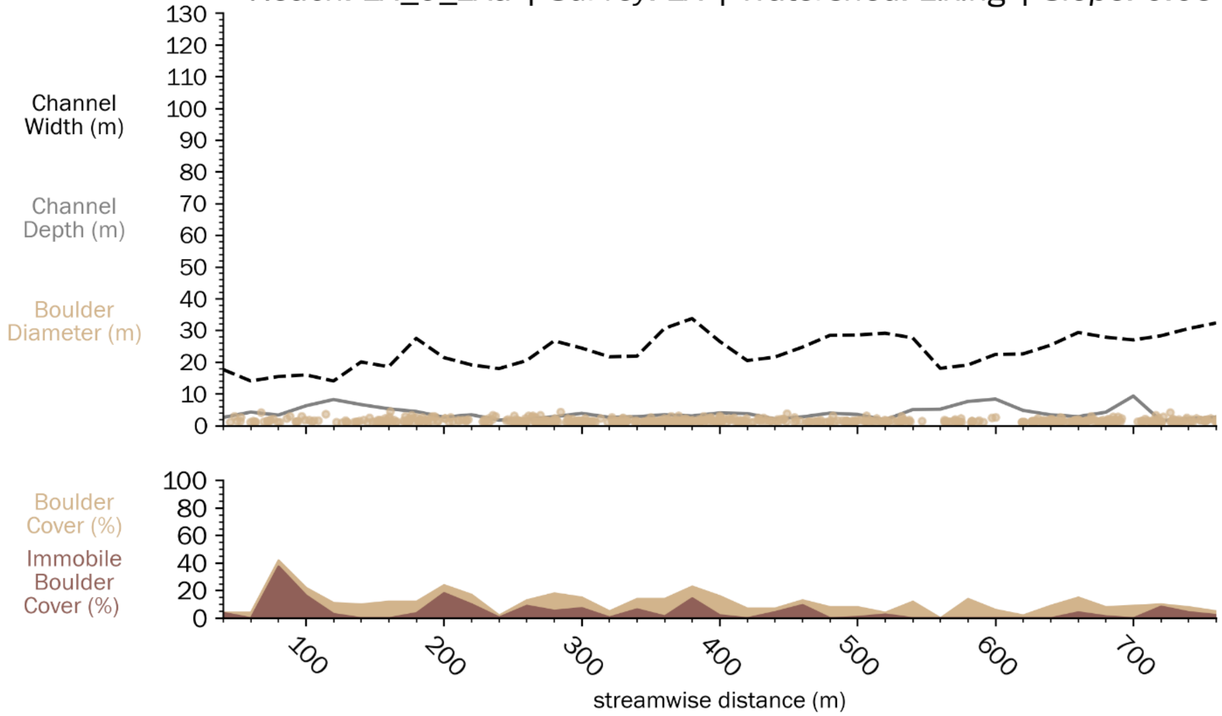

Reach: LXa_LXb | Survey: LX | Watershed: Lixing | Slope: 0.09

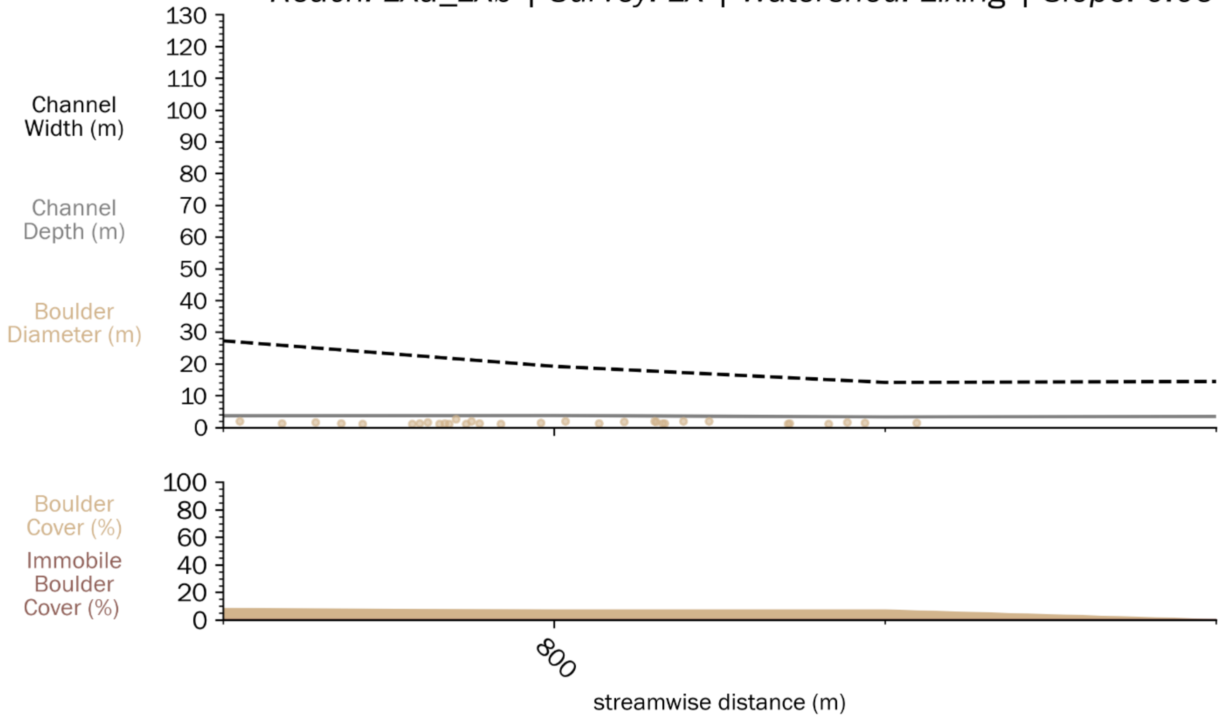

Site: Liqi

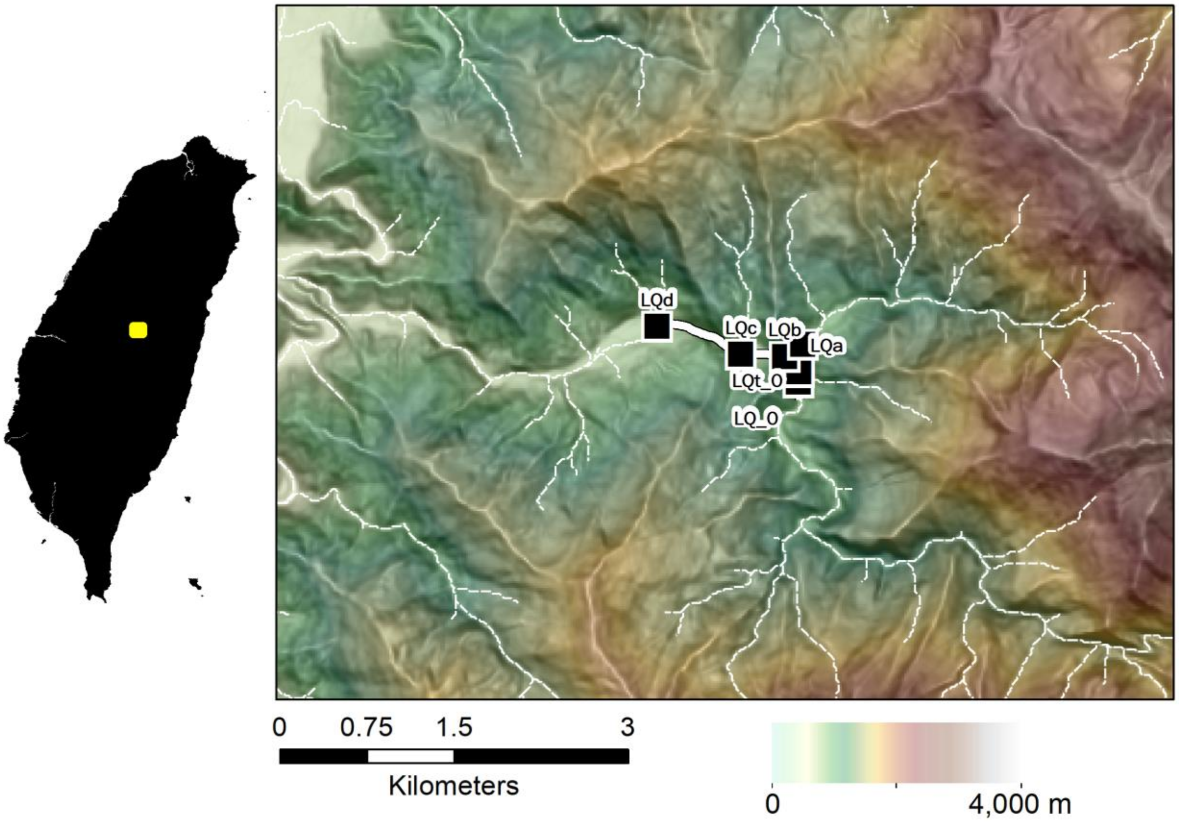

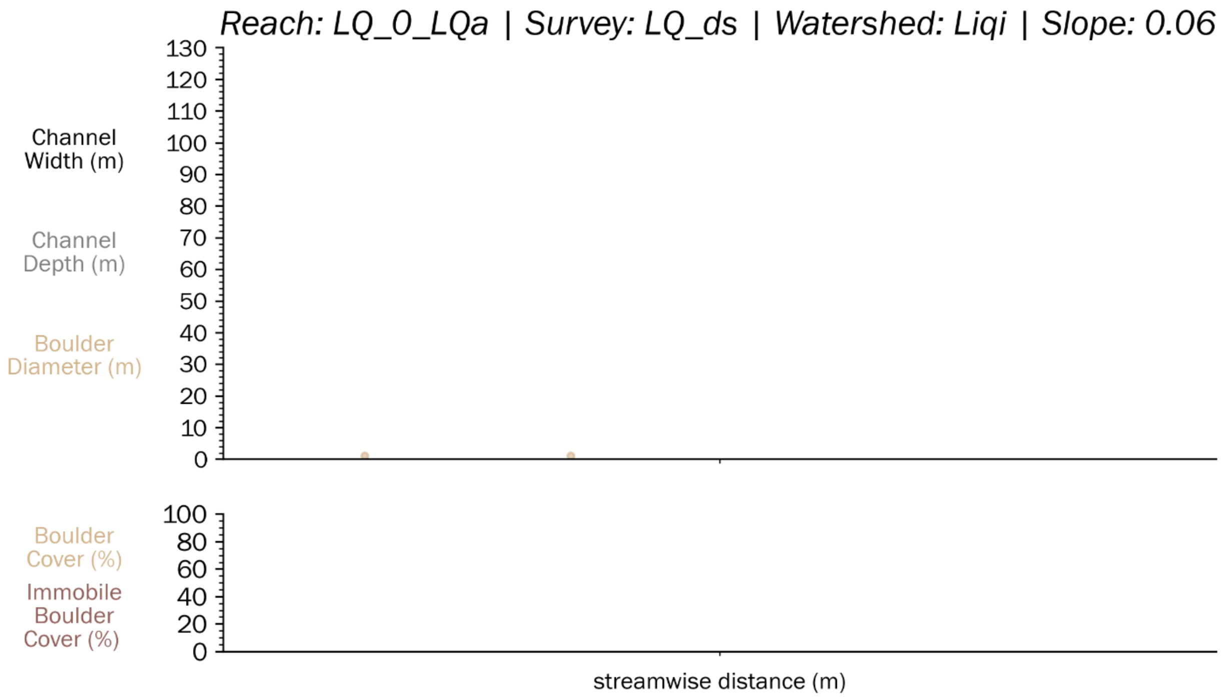

Reach: LQ_0_LQa | Survey: LQ_ds | Watershed: Liqi | Slope: 0.06
Channel Width (m)
Channel Depth (m)
Boulder Diameter (m)
130
120
110
100
90
80
70
60
50
40
30
20
10
0
Boulder Cover (%)
Immobile Boulder Cover (%)
100
80
60
40
20
0
streamwise distance (m)

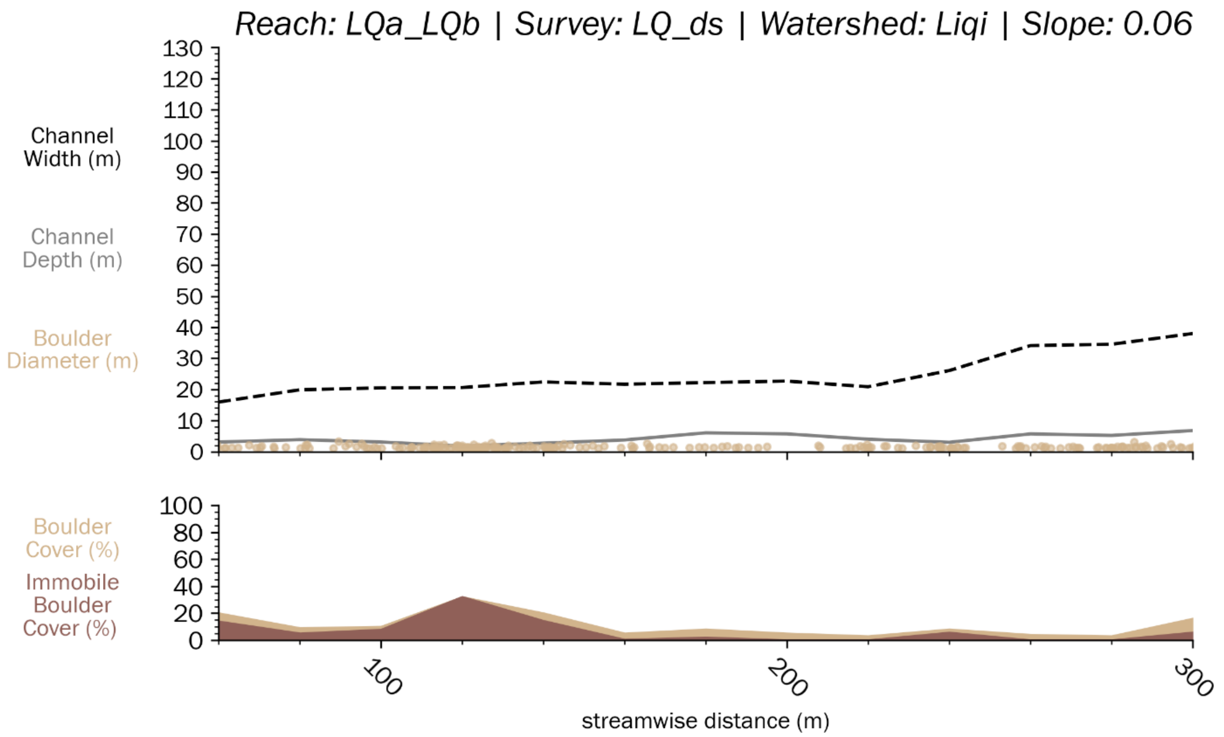

Reach: LQa_LQb | Survey: LQ_ds | Watershed: Liqi | Slope: 0.06
Channel Width (m)
Channel Depth (m)
Boulder Diameter (m)
130
120
110
100
90
80
70
60
50
40
30
20
10
0
Boulder Cover (%)
Immobile Boulder Cover (%)
100
80
60
40
20
0
100
200
300
streamwise distance (m)

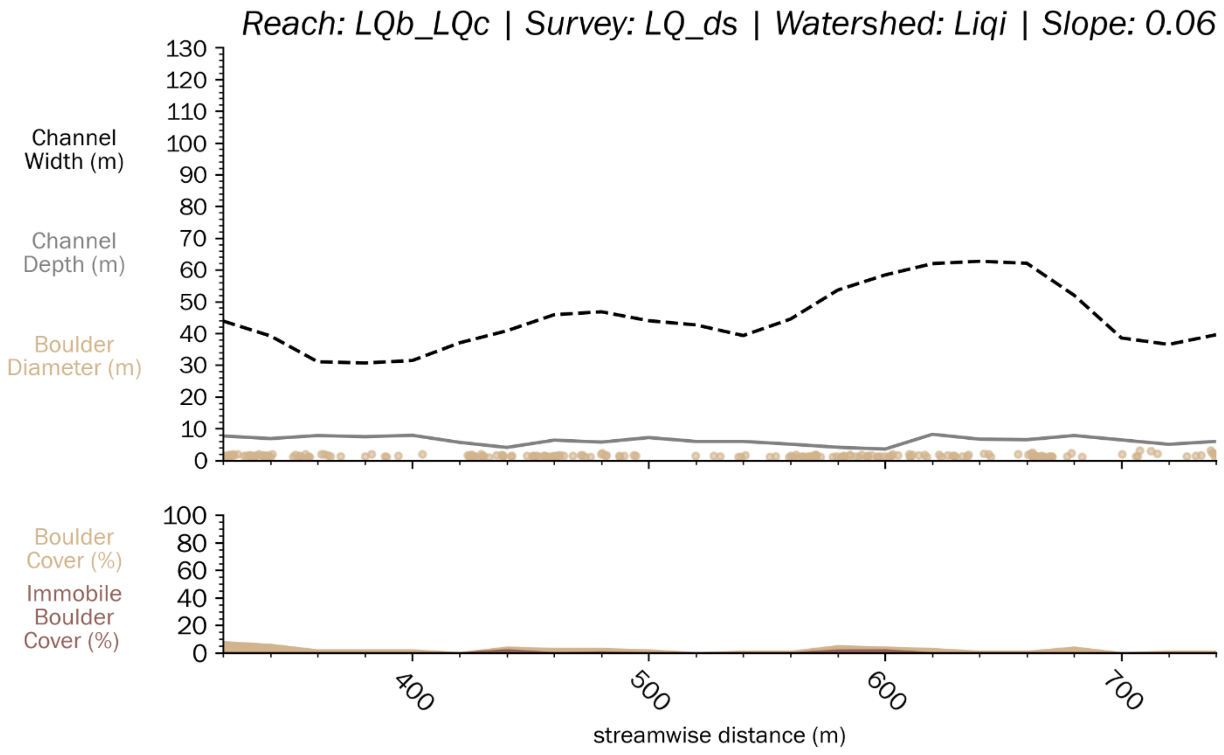
Reach: LQb_LQc | Survey: LQ_ds | Watershed: Liqi | Slope: 0.06
Channel Width (m)
Channel Depth (m)
Boulder Diameter (m)
130
120
110
100
90
80
70
60
50
40
30
20
10
0
Boulder Cover (%)
Immobile Boulder Cover (%)
100
80
60
40
20
0
400
500
600
700
streamwise distance (m)

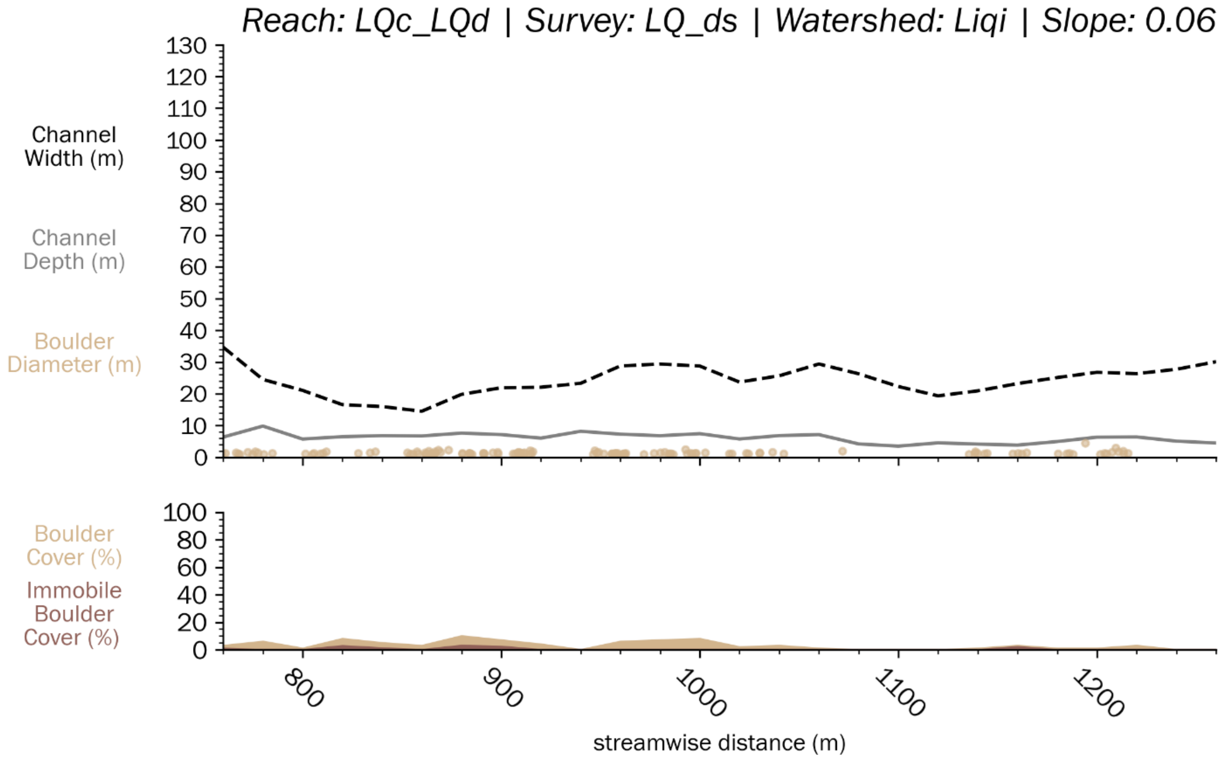
Reach: LQc_LQd | Survey: LQ_ds | Watershed: Liqi | Slope: 0.06
Channel Width (m)
Channel Depth (m)
Boulder Diameter (m)
130
120
110
100
90
80
70
60
50
40
30
20
10
0
Boulder Cover (%)
Immobile Boulder Cover (%)
100
80
60
40
20
0
800
900
1000
1100
1200
streamwise distance (m)

Site: Lakesi

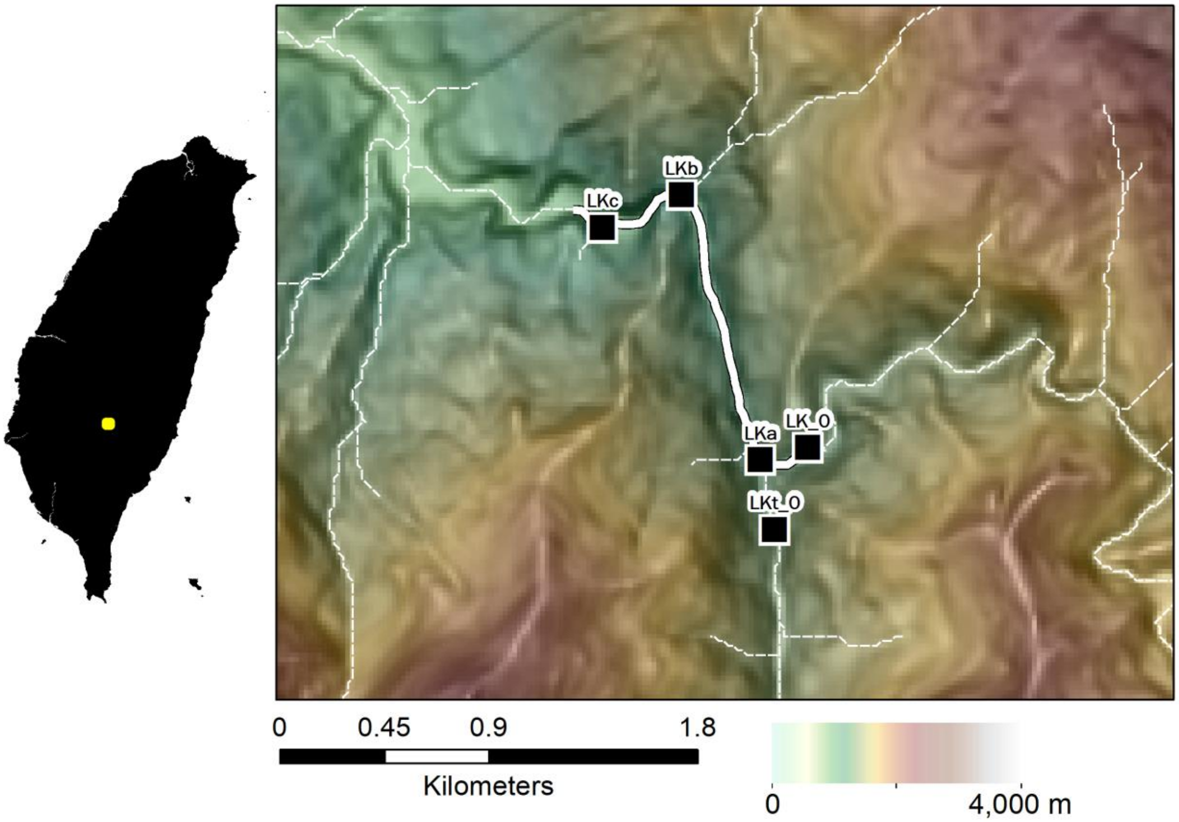

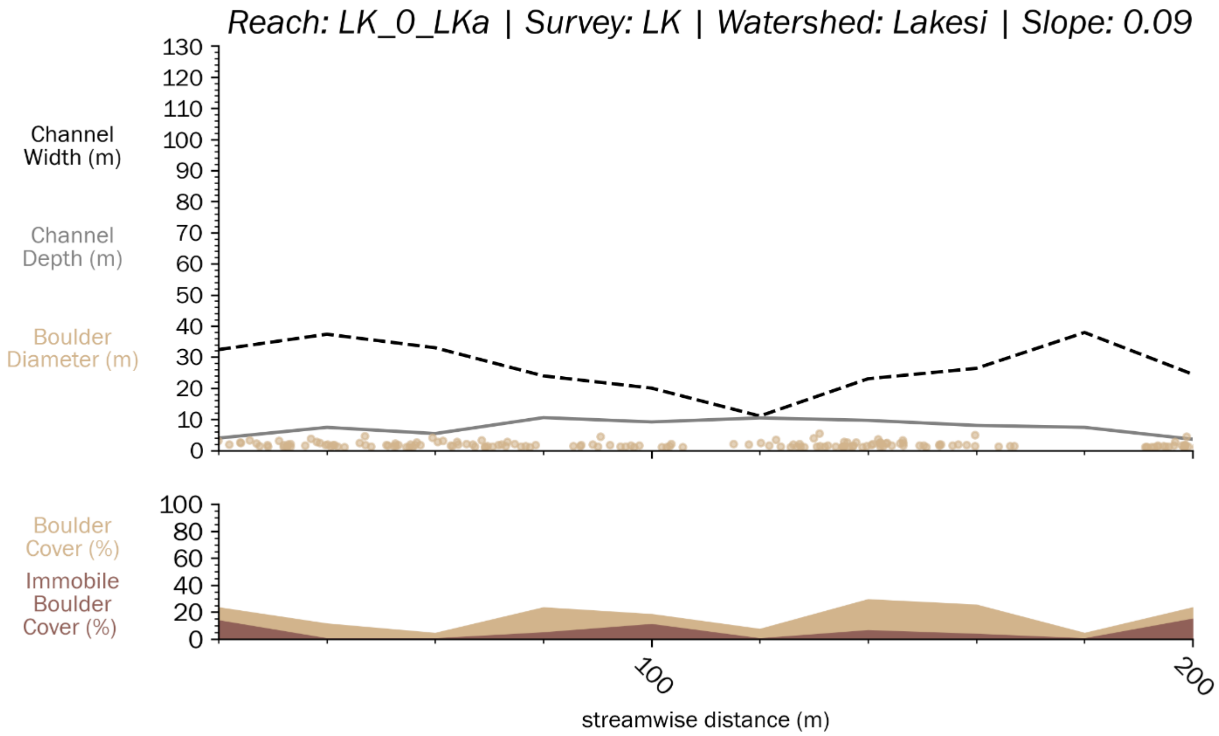

Reach: LK_0_LKa | Survey: LK | Watershed: Lakesi | Slope: 0.09
Channel Width (m)
Channel Depth (m)
Boulder Diameter (m)
Boulder Cover (%)
Immobile Boulder Cover (%)
streamwise distance (m)

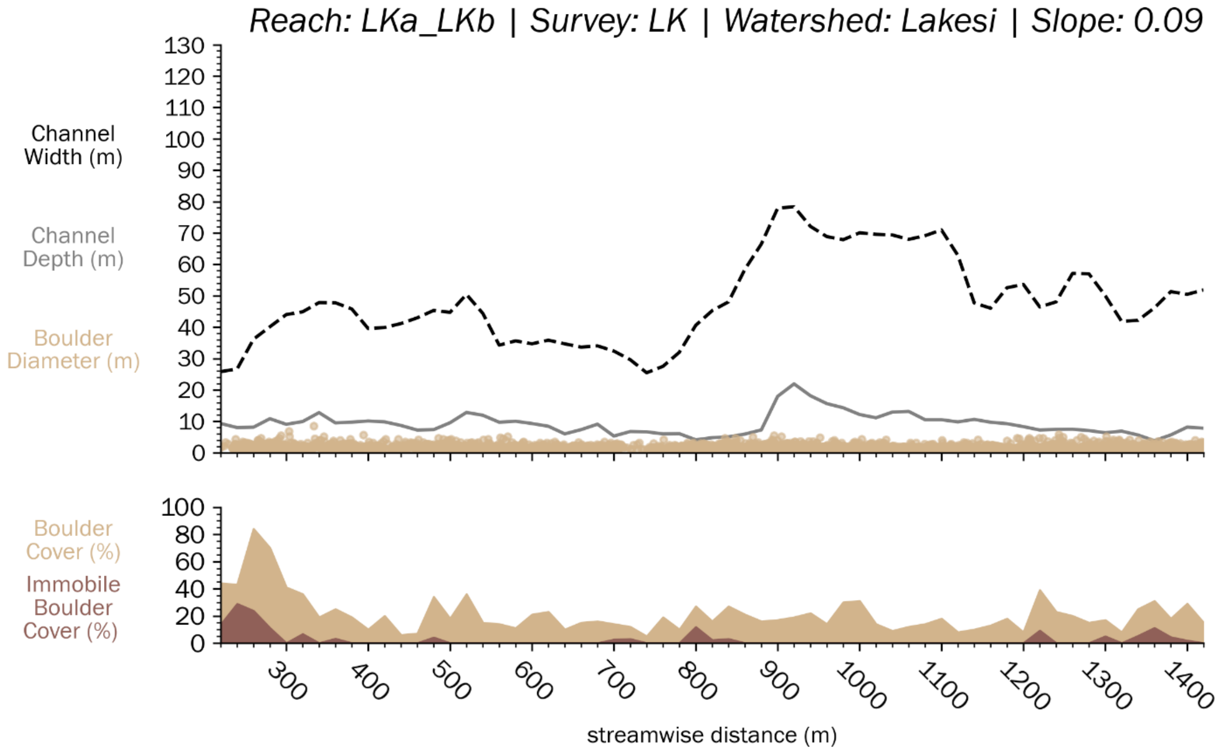

Reach: LKa_LKb | Survey: LK | Watershed: Lakesi | Slope: 0.09
Channel Width (m)
Channel Depth (m)
Boulder Diameter (m)
Boulder Cover (%)
Immobile Boulder Cover (%)
streamwise distance (m)

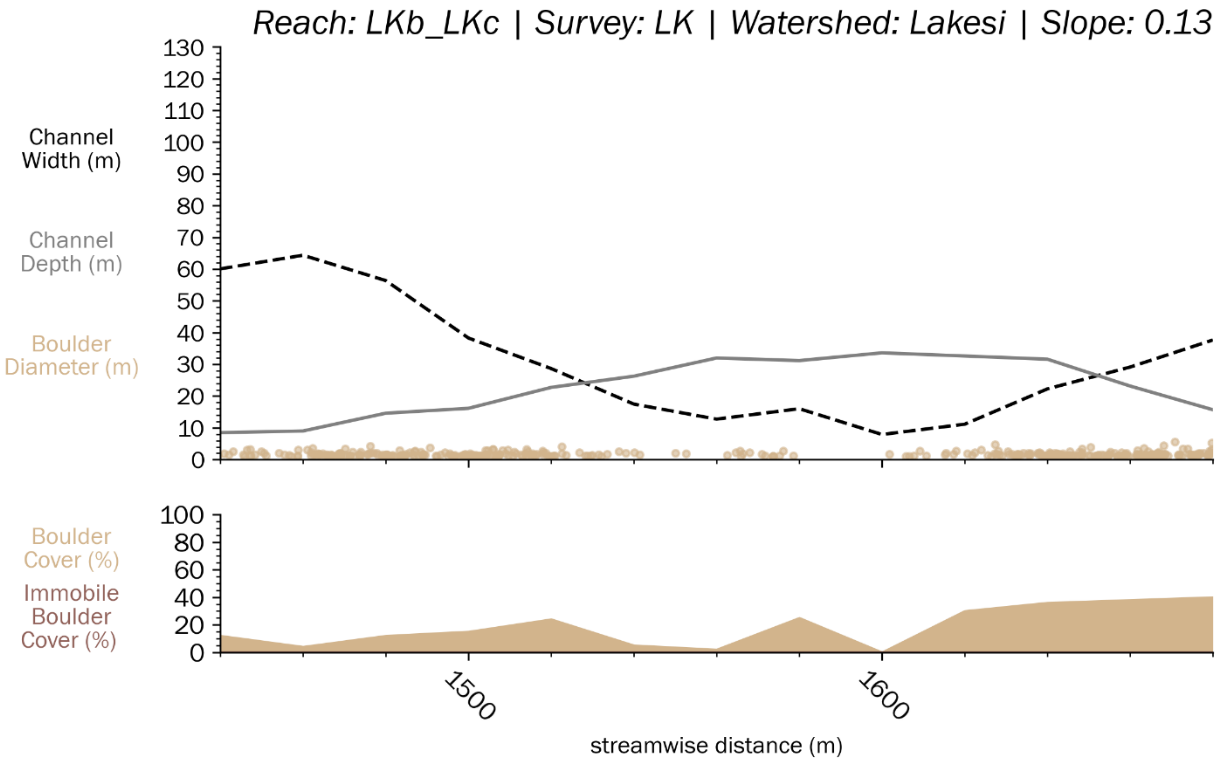

Reach: LKb_LKc | Survey: LK | Watershed: Lakesi | Slope: 0.13
Channel Width (m)
Channel Depth (m)
Boulder Diameter (m)
Boulder Cover (%)
Immobile Boulder Cover (%)
130
120
110
100
90
80
70
60
50
40
30
20
10
0
100
80
60
40
20
0
1500
1600
streamwise distance (m)

Region: South

Site: Zhiben

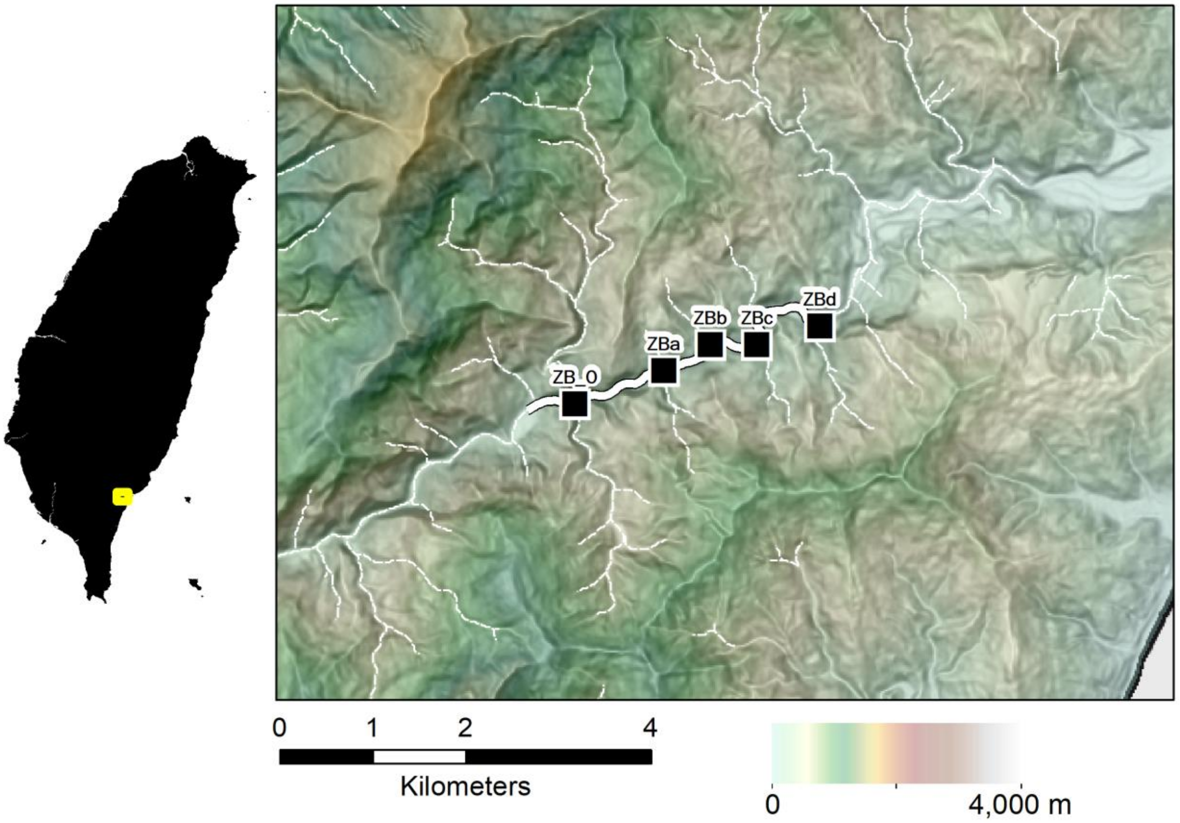

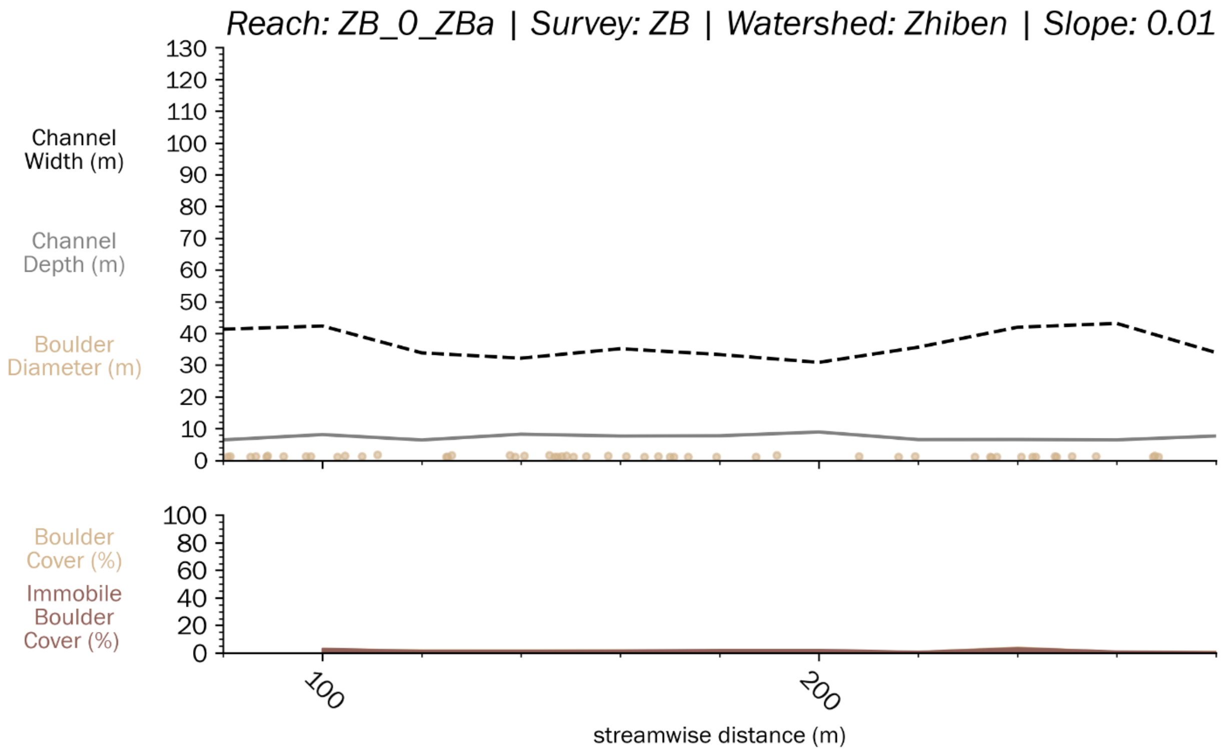
Reach: ZB_0_ZBa | Survey: ZB | Watershed: Zhiben | Slope: 0.01
Channel Width (m)
Channel Depth (m)
Boulder Diameter (m)
Boulder Cover (%)
Immobile Boulder Cover (%)
0
10
20
30
40
50
60
70
80
90
100
110
120
130
100
200
streamwise distance (m)

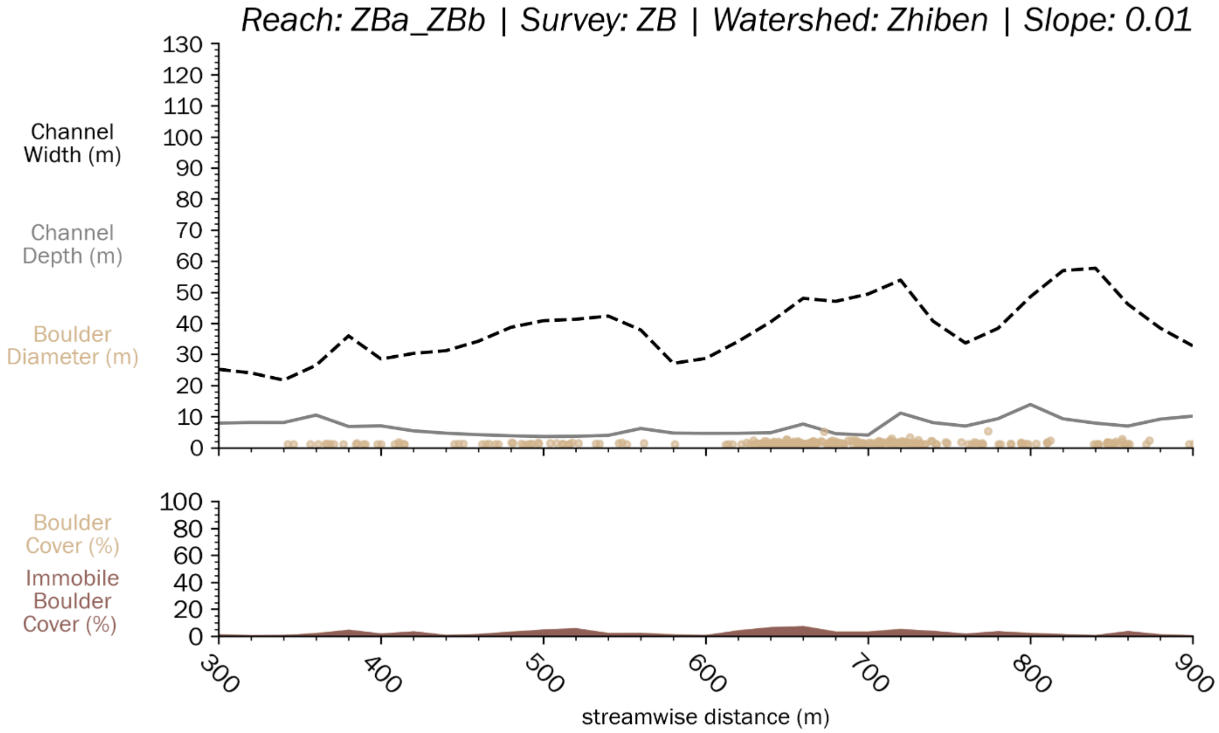
Reach: ZBa_ZBb | Survey: ZB | Watershed: Zhiben | Slope: 0.01
Channel Width (m)
Channel Depth (m)
Boulder Diameter (m)
Boulder Cover (%)
Immobile Boulder Cover (%)
0
10
20
30
40
50
60
70
80
90
100
110
120
130
300
400
500
600
700
800
900
streamwise distance (m)

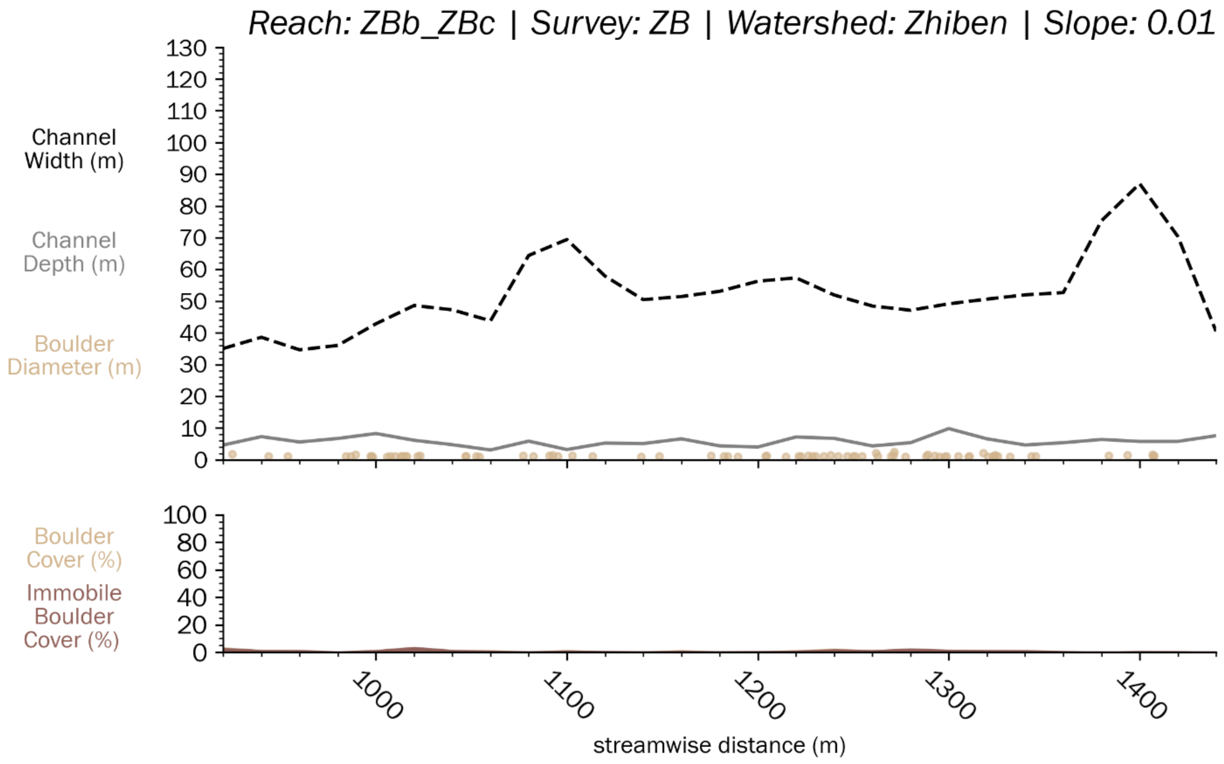

Reach: ZBb_ZBc | Survey: ZB | Watershed: Zhiben | Slope: 0.01
Channel Width (m)
Channel Depth (m)
Boulder Diameter (m)
Boulder Cover (%)
Immobile Boulder Cover (%)
130
120
110
100
90
80
70
60
50
40
30
20
10
0
100
80
60
40
20
0
1000
1100
1200
1300
1400
streamwise distance (m)

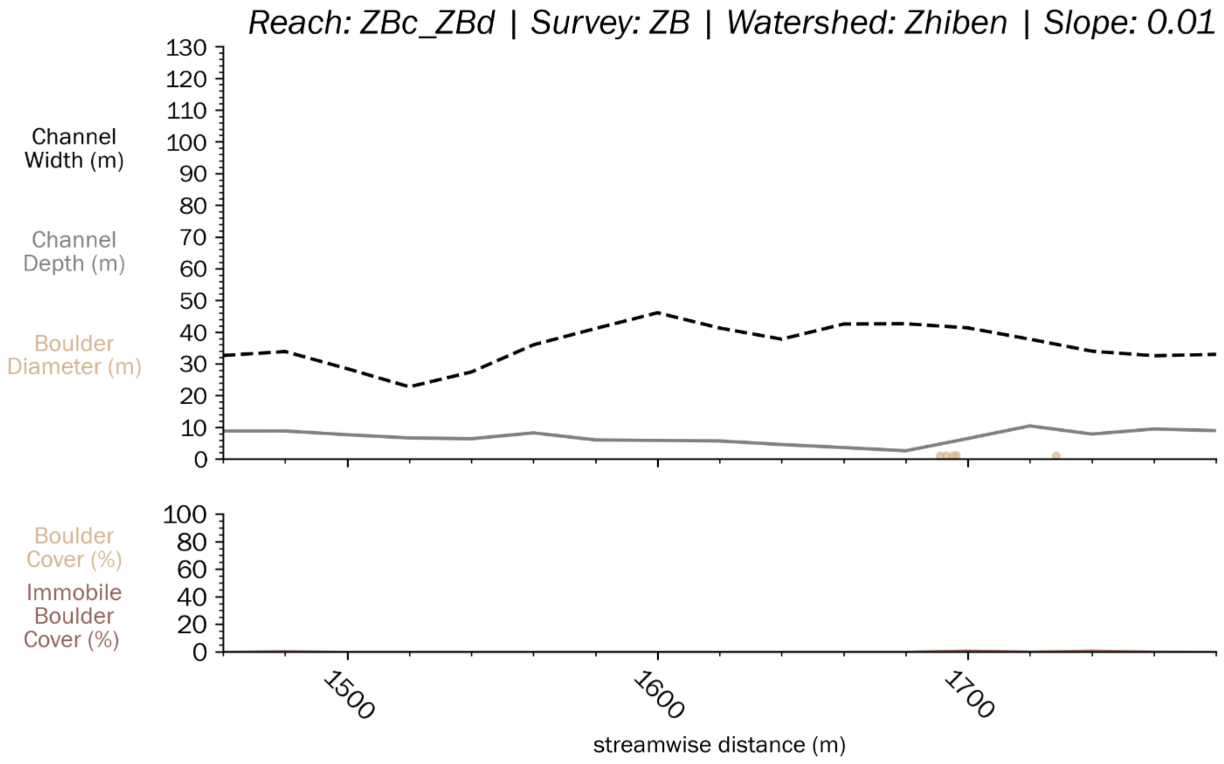

Reach: ZBc_ZBd | Survey: ZB | Watershed: Zhiben | Slope: 0.01
Channel Width (m)
Channel Depth (m)
Boulder Diameter (m)
Boulder Cover (%)
Immobile Boulder Cover (%)
130
120
110
100
90
80
70
60
50
40
30
20
10
0
100
80
60
40
20
0
1500
1600
1700
streamwise distance (m)

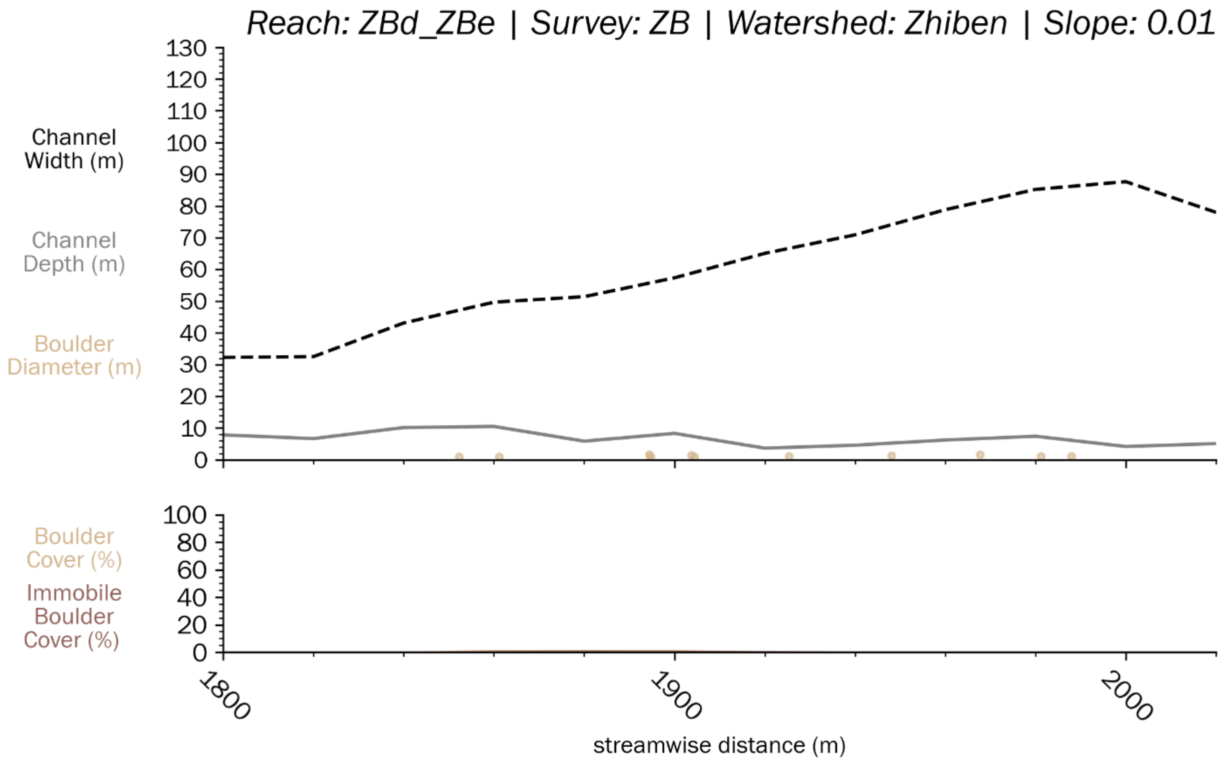
Reach: ZBd_ZBe | Survey: ZB | Watershed: Zhiben | Slope: 0.01
Channel Width (m)
Channel Depth (m)
Boulder Diameter (m)
Boulder Cover (%)
Immobile Boulder Cover (%)
130
120
110
100
90
80
70
60
50
40
30
20
10
0
100
80
60
40
20
0
1800
1900
2000
streamwise distance (m)

Site: Luye

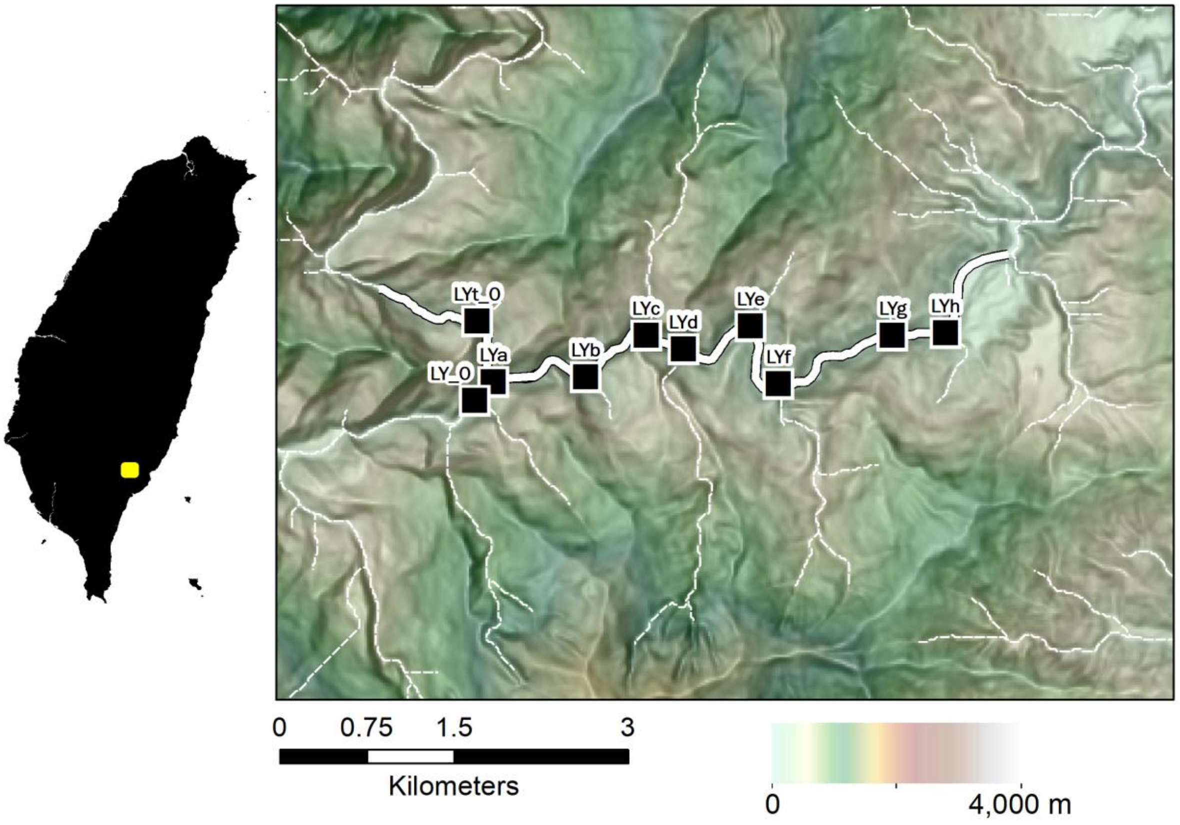
LYt_0
LYa
LY_0
LYb
LYc
LYd
LYe
LYf
LYg
LYh
0 0.75 1.5 3
Kilometers
0
4,000 m

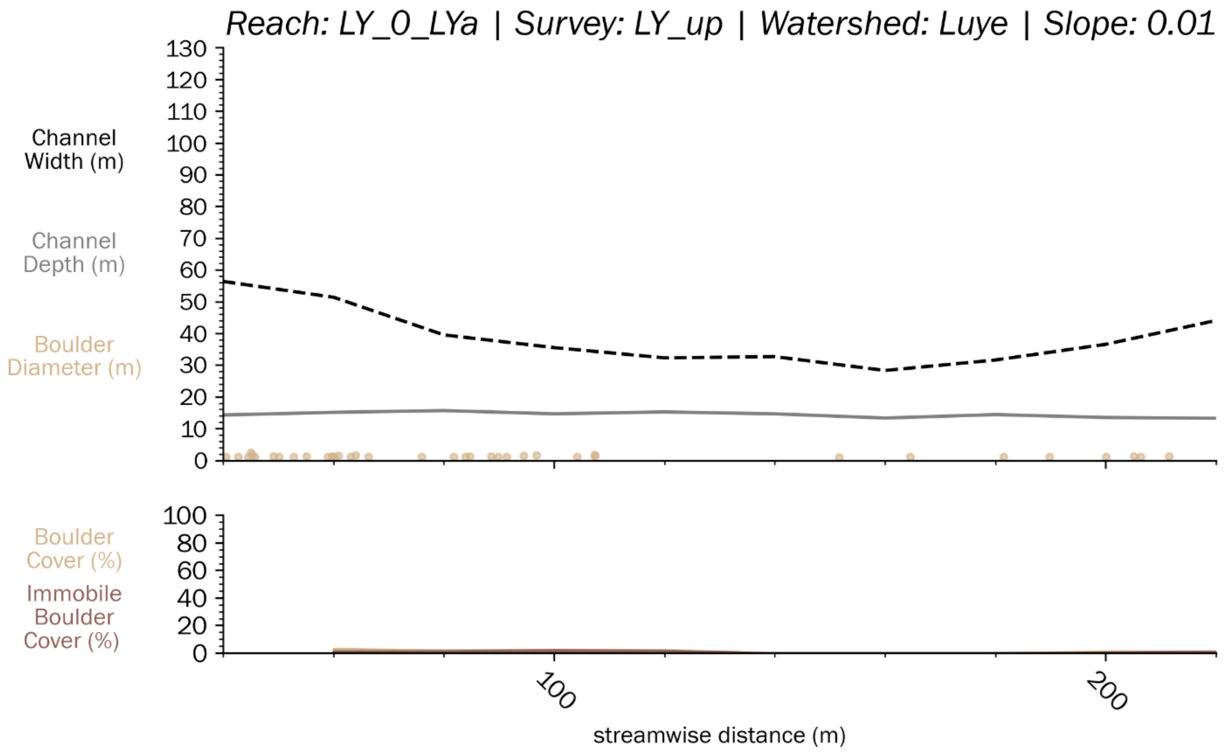
Reach: LY_0_LYa | Survey: LY_up | Watershed: Luye | Slope: 0.01
Channel Width (m)
Channel Depth (m)
Boulder Diameter (m)
Boulder Cover (%)
Immobile Boulder Cover (%)
100
200
streamwise distance (m)

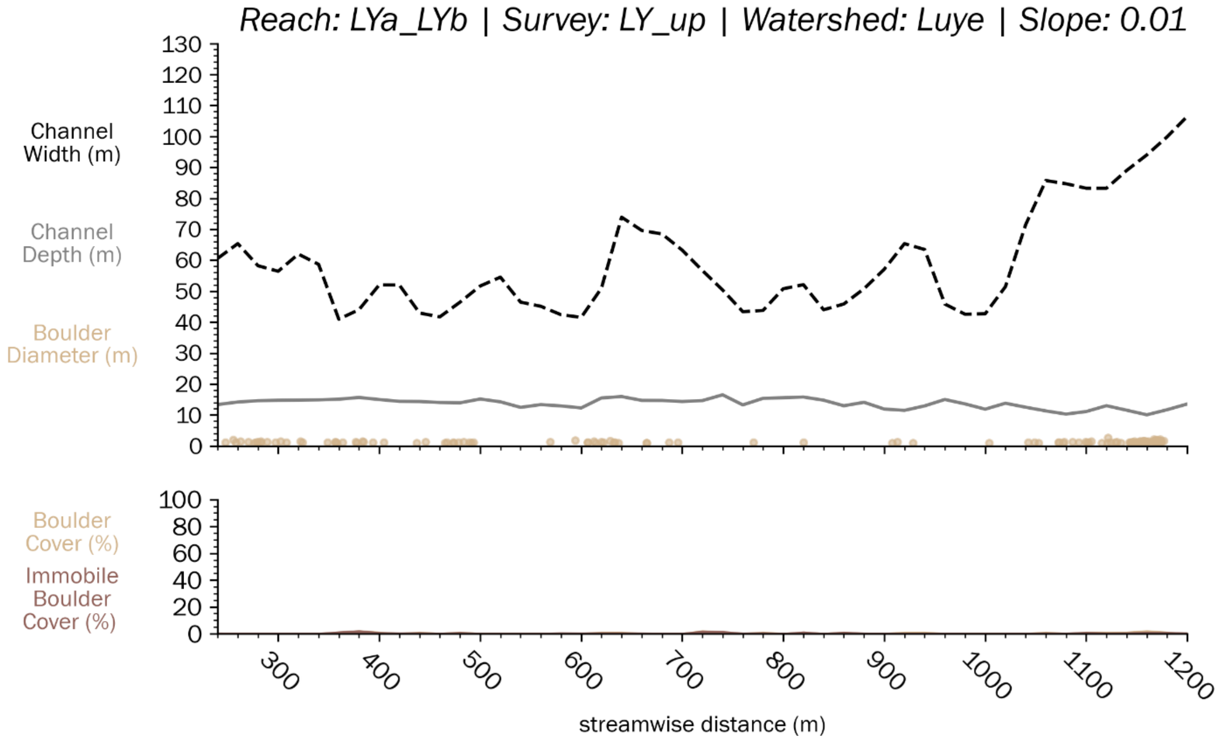
Reach: LYa_LYb | Survey: LY_up | Watershed: Luye | Slope: 0.01
Channel Width (m)
Channel Depth (m)
Boulder Diameter (m)
Boulder Cover (%)
Immobile Boulder Cover (%)
300
400
500
600
700
800
900
1000
1100
1200
streamwise distance (m)

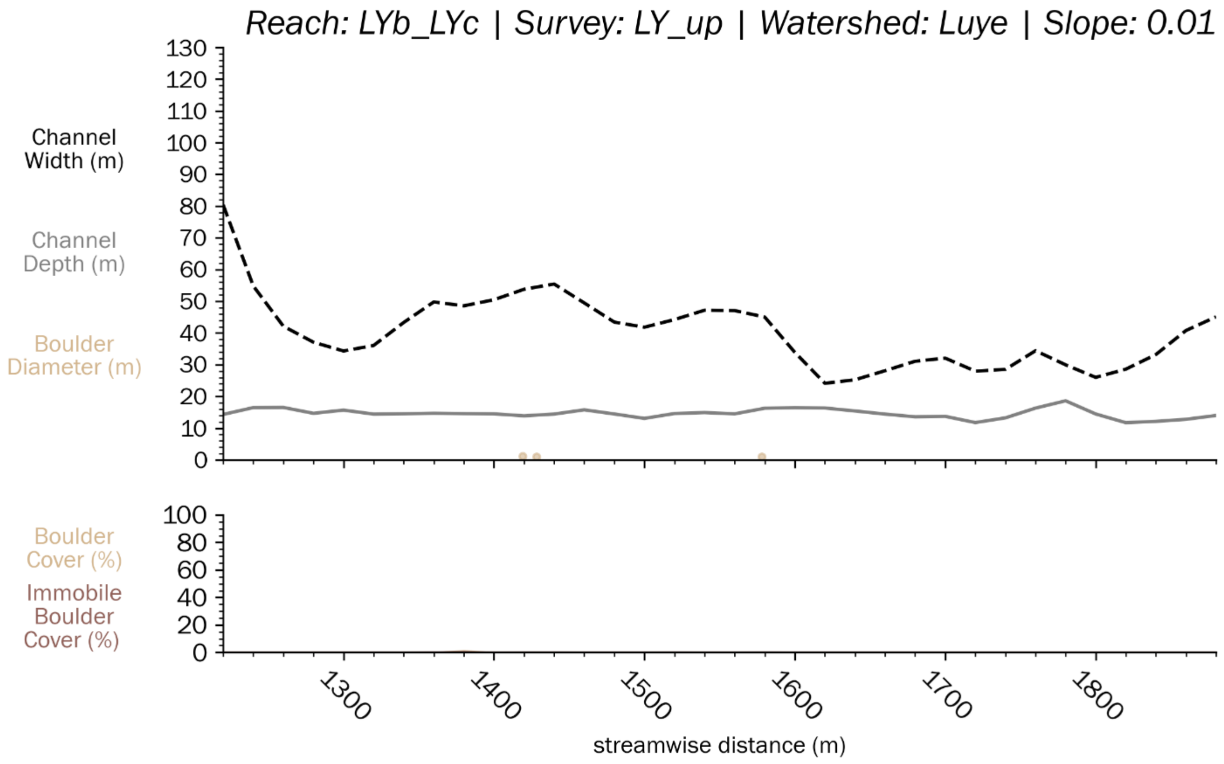

Reach: LYb_LYc | Survey: LY_up | Watershed: Luye | Slope: 0.01
Channel Width (m)
Channel Depth (m)
Boulder Diameter (m)
Boulder Cover (%)
Immobile Boulder Cover (%)
streamwise distance (m)

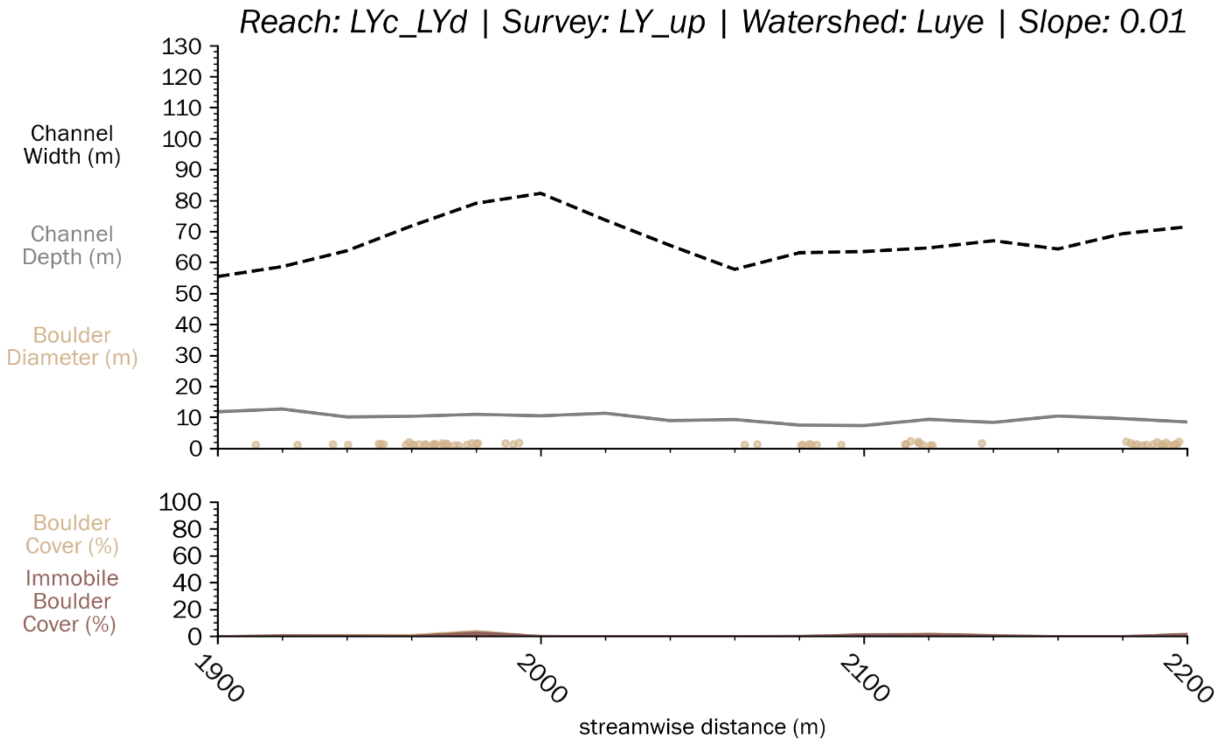

Reach: LYc_LYd | Survey: LY_up | Watershed: Luye | Slope: 0.01
Channel Width (m)
Channel Depth (m)
Boulder Diameter (m)
Boulder Cover (%)
Immobile Boulder Cover (%)
streamwise distance (m)

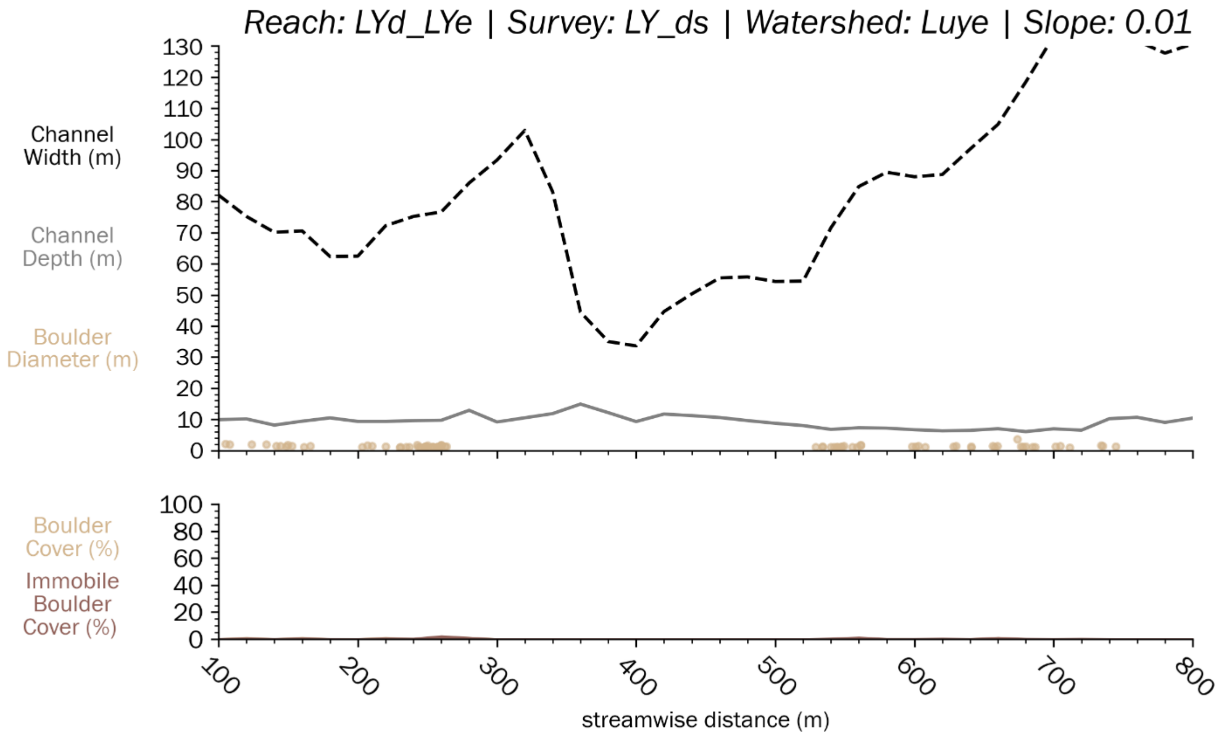

Reach: LYd_LYe | Survey: LY_ds | Watershed: Luye | Slope: 0.01
Channel Width (m)
Channel Depth (m)
Boulder Diameter (m)
Boulder Cover (%)
Immobile Boulder Cover (%)
streamwise distance (m)

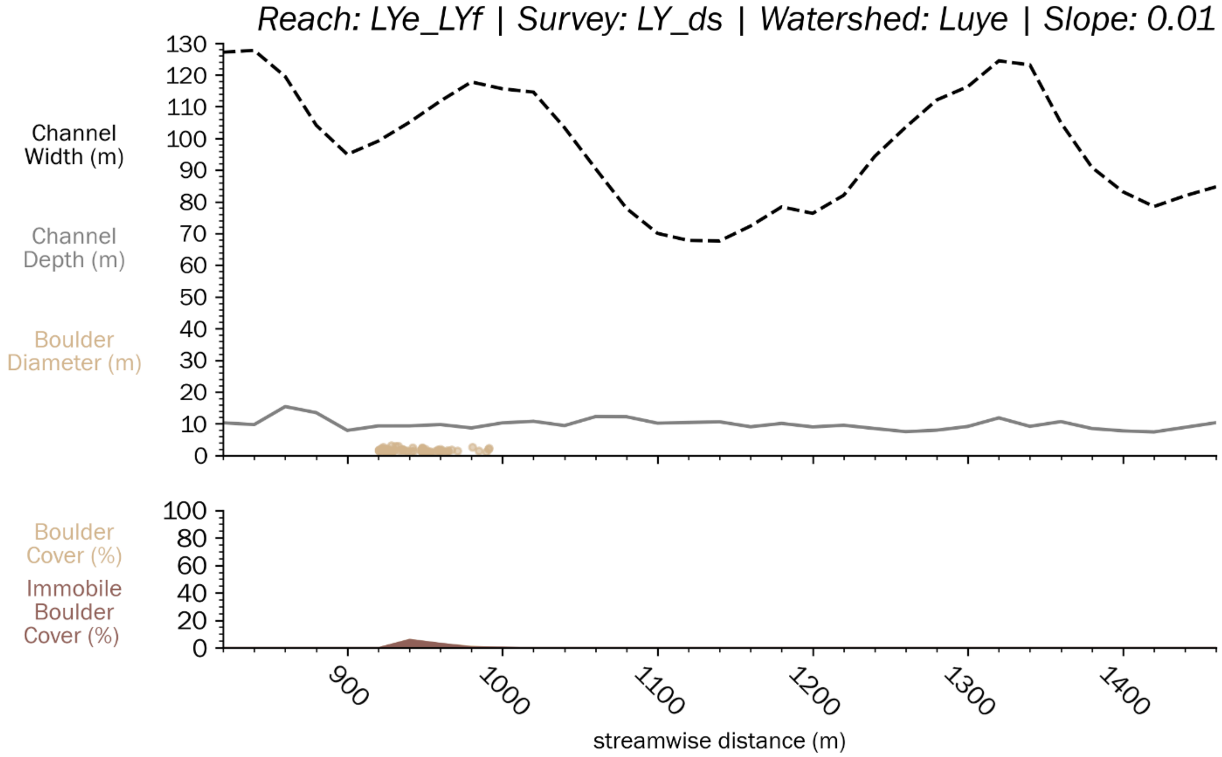

Reach: LYe_LYf | Survey: LY_ds | Watershed: Luye | Slope: 0.01
Channel Width (m)
Channel Depth (m)
Boulder Diameter (m)
Boulder Cover (%)
Immobile Boulder Cover (%)
streamwise distance (m)

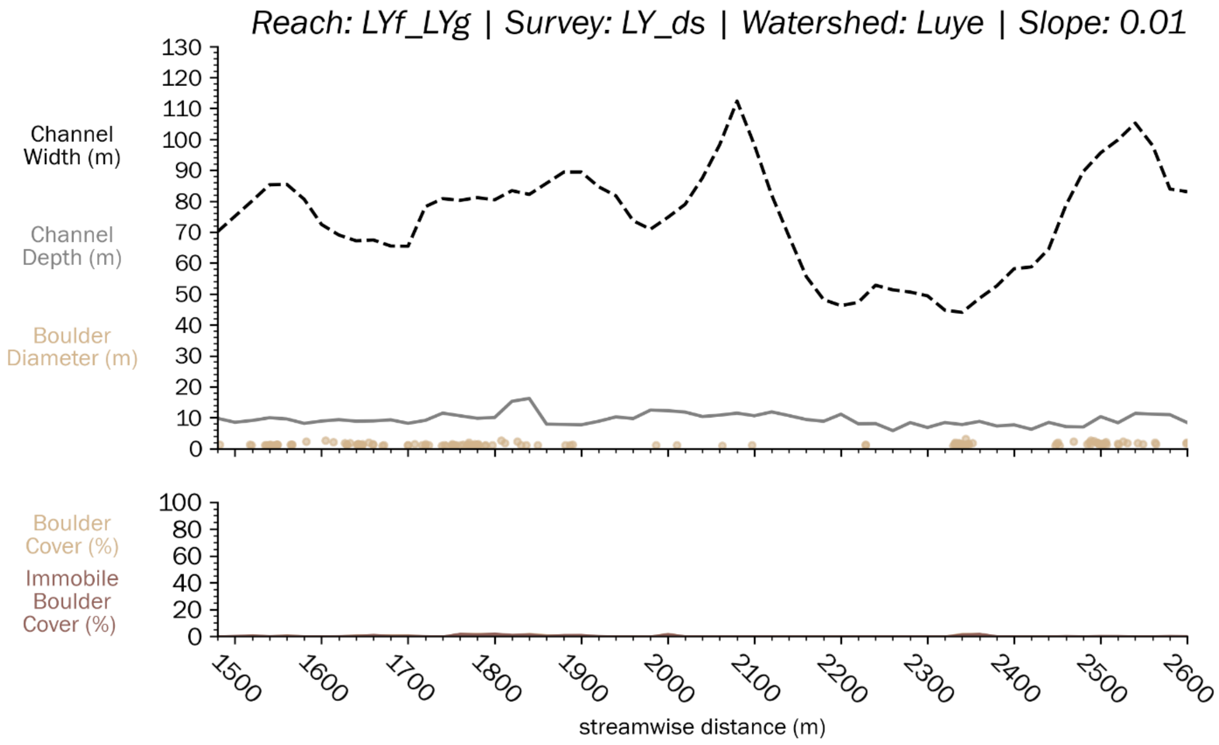
Reach: LYf_LYg | Survey: LY_ds | Watershed: Luye | Slope: 0.01
Channel Width (m)
Channel Depth (m)
Boulder Diameter (m)
Boulder Cover (%)
Immobile Boulder Cover (%)
streamwise distance (m)

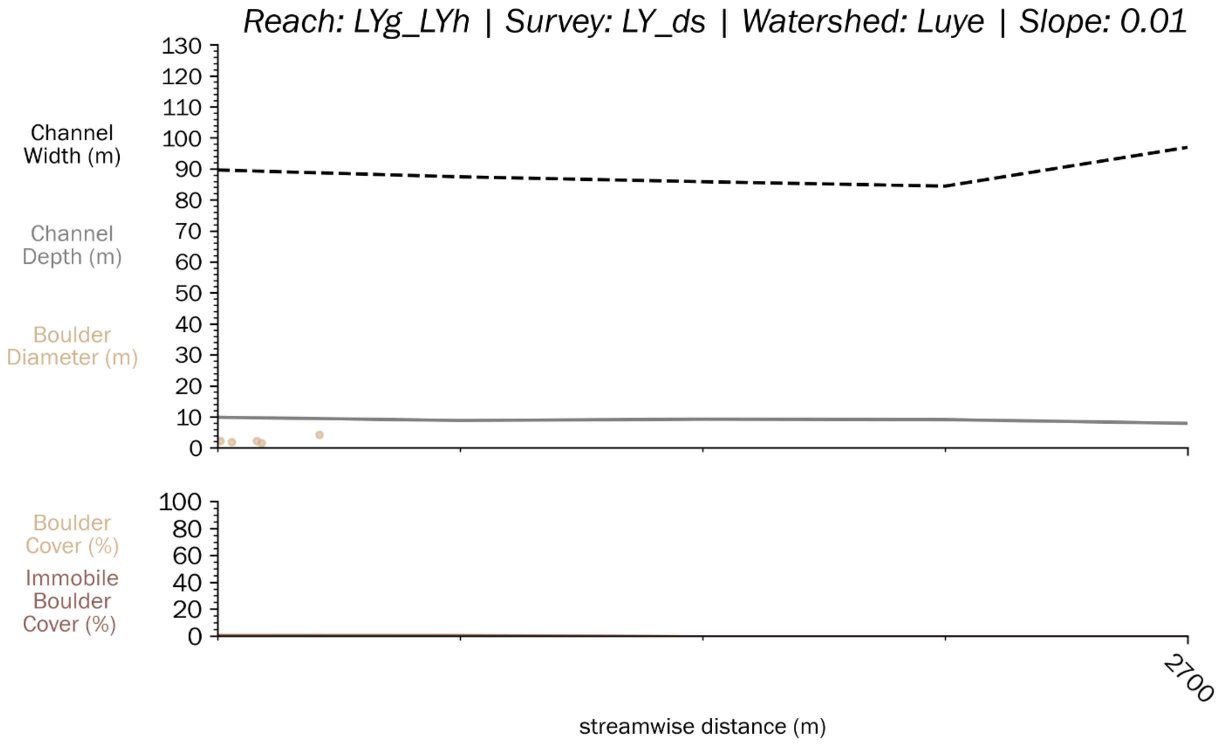
Reach: LYg_LYh | Survey: LY_ds | Watershed: Luye | Slope: 0.01
Channel Width (m)
Channel Depth (m)
Boulder Diameter (m)
Boulder Cover (%)
Immobile Boulder Cover (%)
streamwise distance (m)

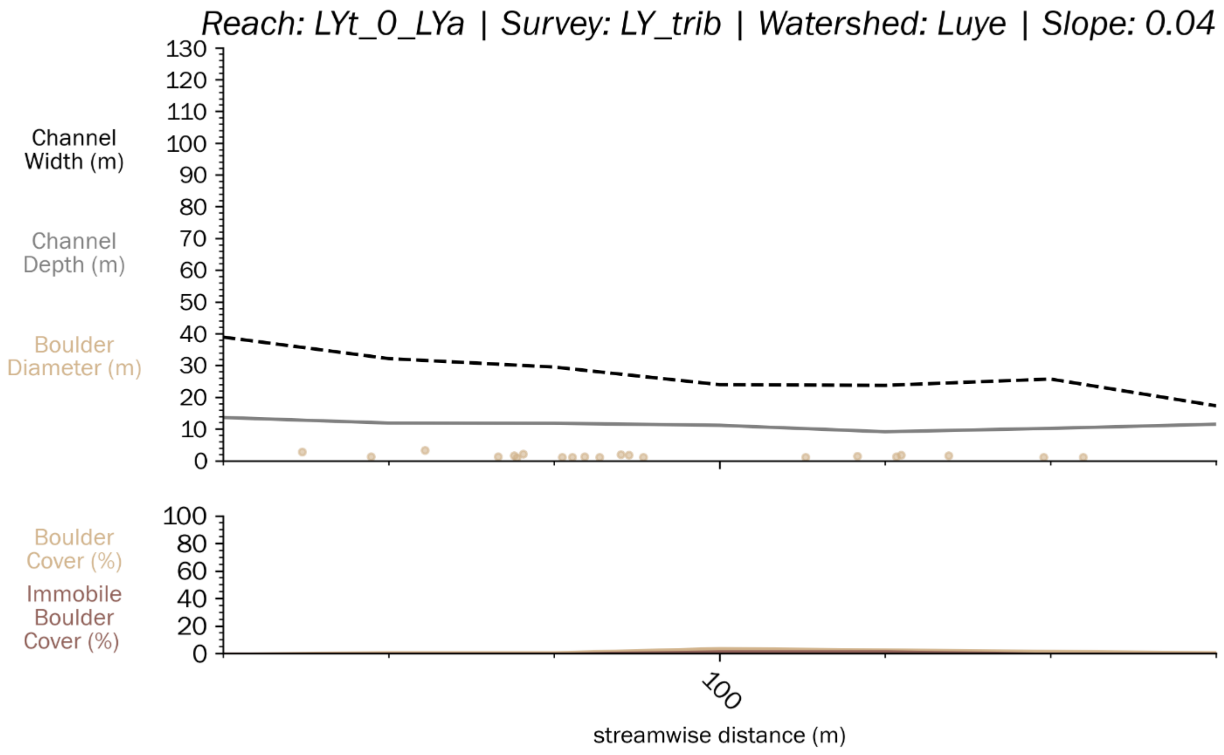
Reach: LYt_0_LYa | Survey: LY_trib | Watershed: Luye | Slope: 0.04
Channel Width (m)
Channel Depth (m)
Boulder Diameter (m)
130
120
110
100
90
80
70
60
50
40
30
20
10
0
Boulder Cover (%)
Immobile Boulder Cover (%)
100
80
60
40
20
0
100
streamwise distance (m)

Region: Central

Site: Cross Island Highway, Xinwulu basin

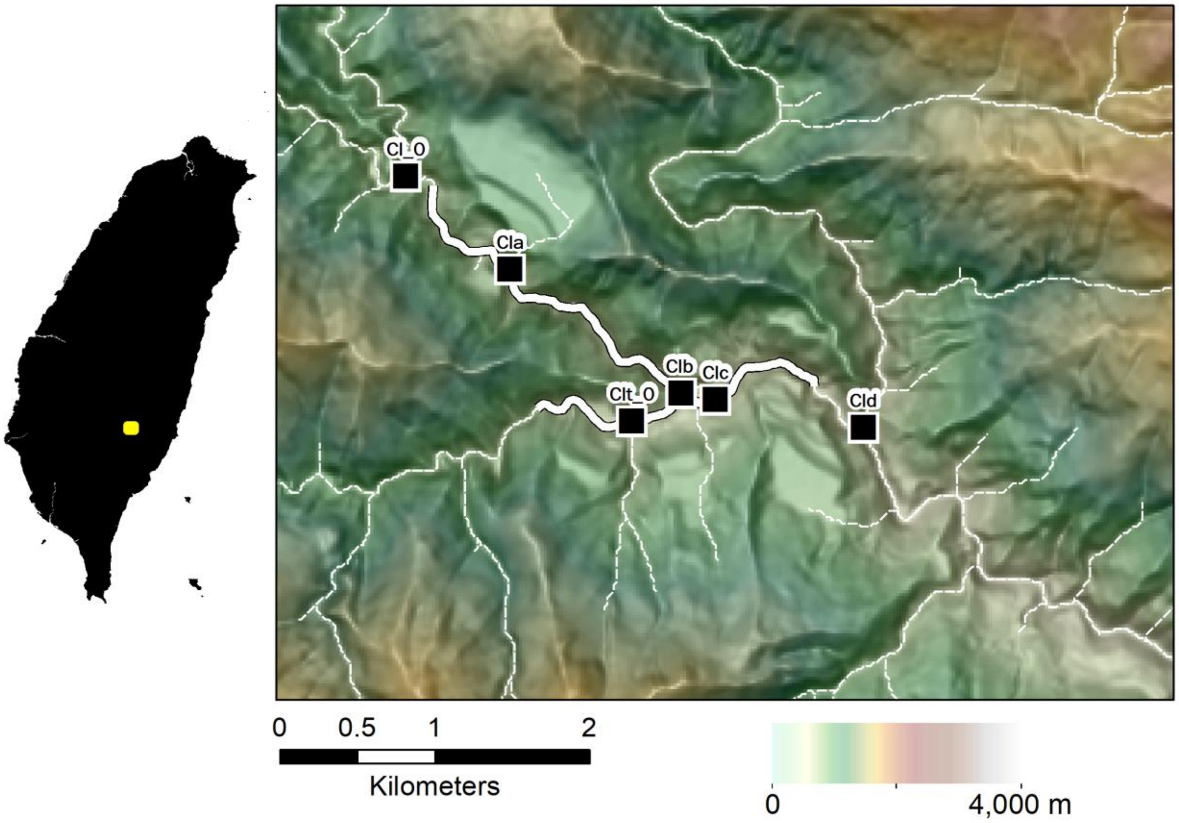

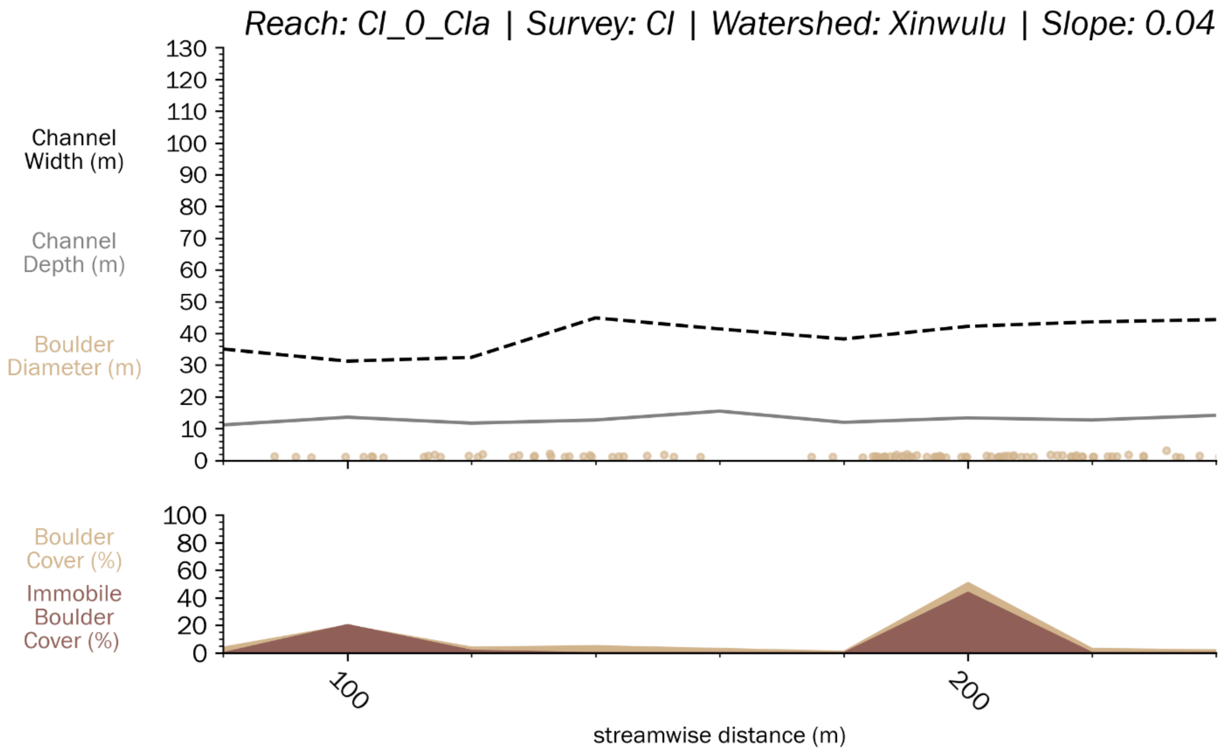

Reach: CI_0_CIa | Survey: CI | Watershed: Xinwulu | Slope: 0.04
Channel Width (m)
Channel Depth (m)
Boulder Diameter (m)
Boulder Cover (%)
Immobile Boulder Cover (%)
streamwise distance (m)

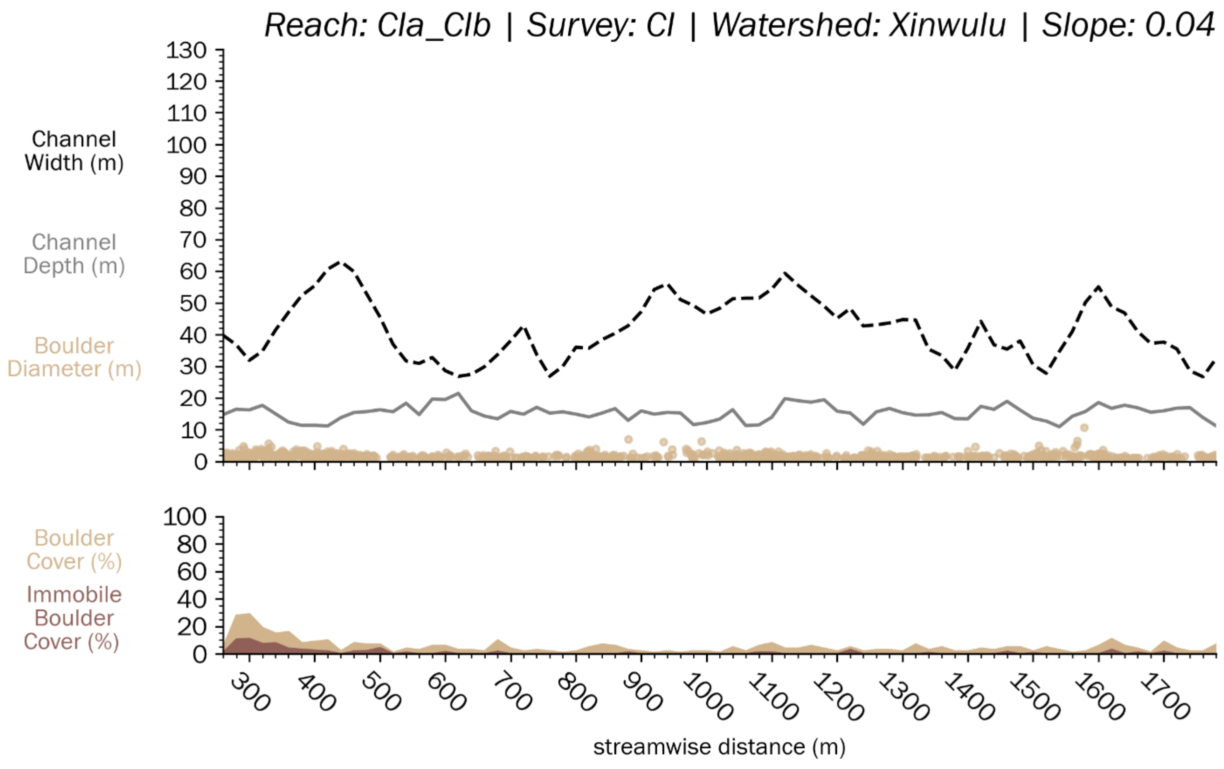

Reach: CIa_CIb | Survey: CI | Watershed: Xinwulu | Slope: 0.04
Channel Width (m)
Channel Depth (m)
Boulder Diameter (m)
Boulder Cover (%)
Immobile Boulder Cover (%)
streamwise distance (m)

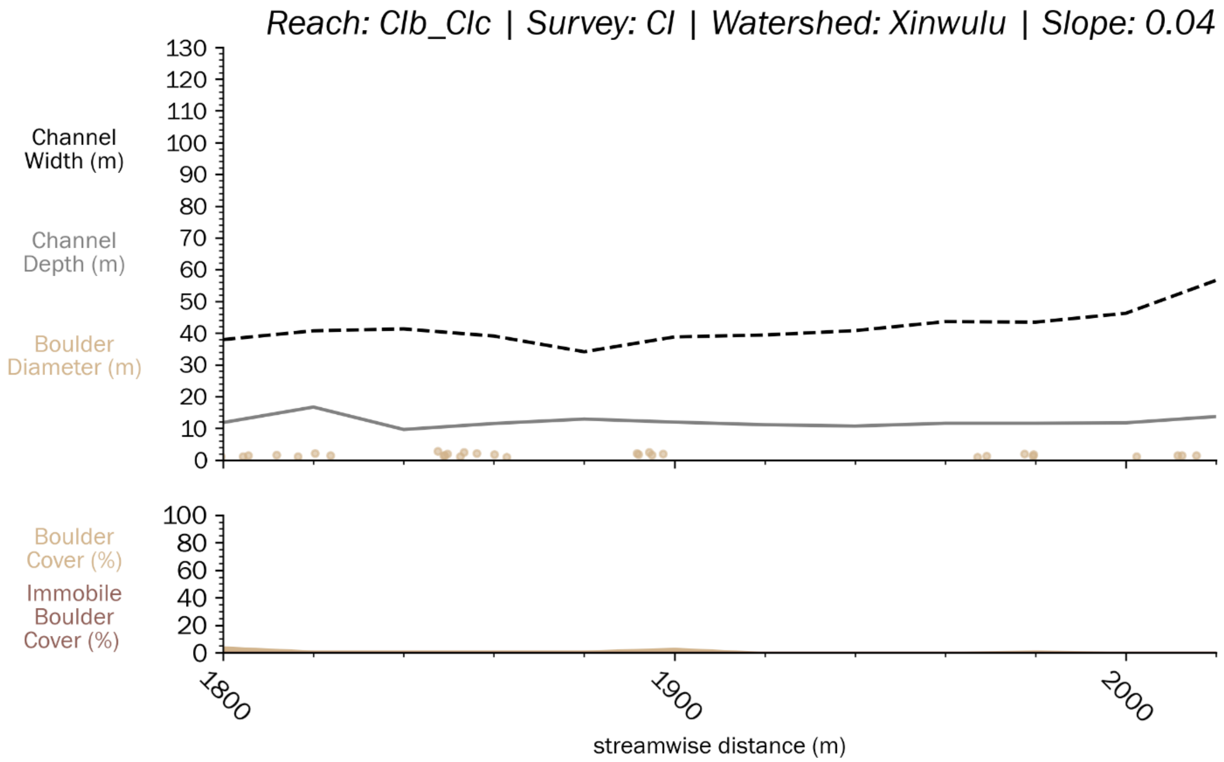

Reach: CIb_CIc | Survey: CI | Watershed: Xinwulu | Slope: 0.04
Channel Width (m)
Channel Depth (m)
Boulder Diameter (m)
Boulder Cover (%)
Immobile Boulder Cover (%)
1800
1900
2000
streamwise distance (m)

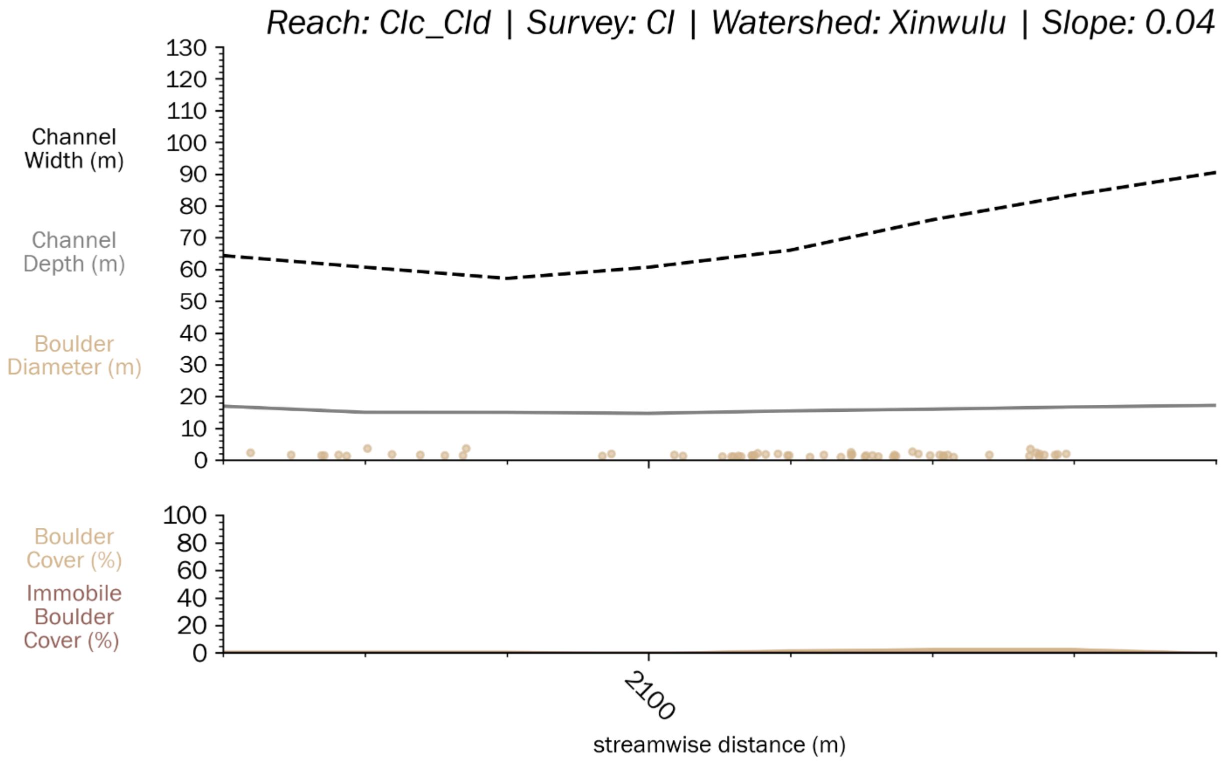

Reach: CIc_CId | Survey: CI | Watershed: Xinwulu | Slope: 0.04
Channel Width (m)
Channel Depth (m)
Boulder Diameter (m)
Boulder Cover (%)
Immobile Boulder Cover (%)
2100
streamwise distance (m)

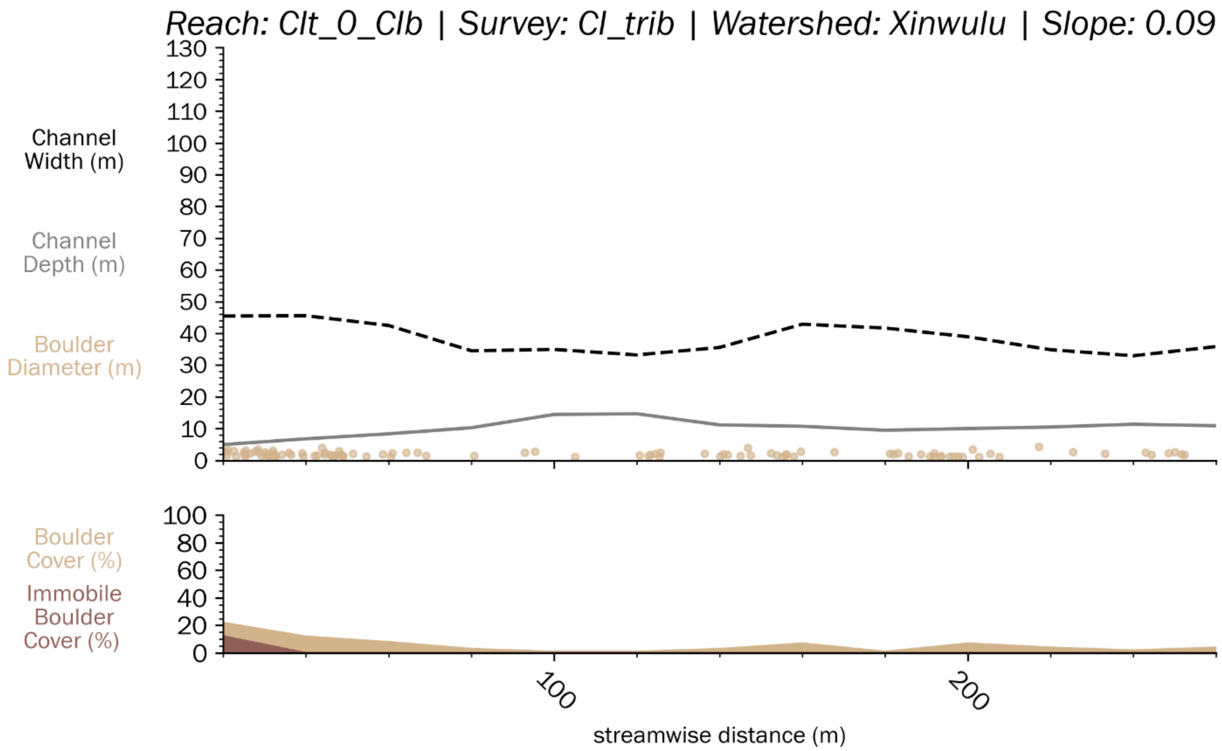
Reach: Clt_0_Clb | Survey: Cl_trib | Watershed: Xinwulu | Slope: 0.09
Channel Width (m)
Channel Depth (m)
Boulder Diameter (m)
Boulder Cover (%)
Immobile Boulder Cover (%)
100
200
streamwise distance (m)

Site: Xinwulu, Xinwulu basin

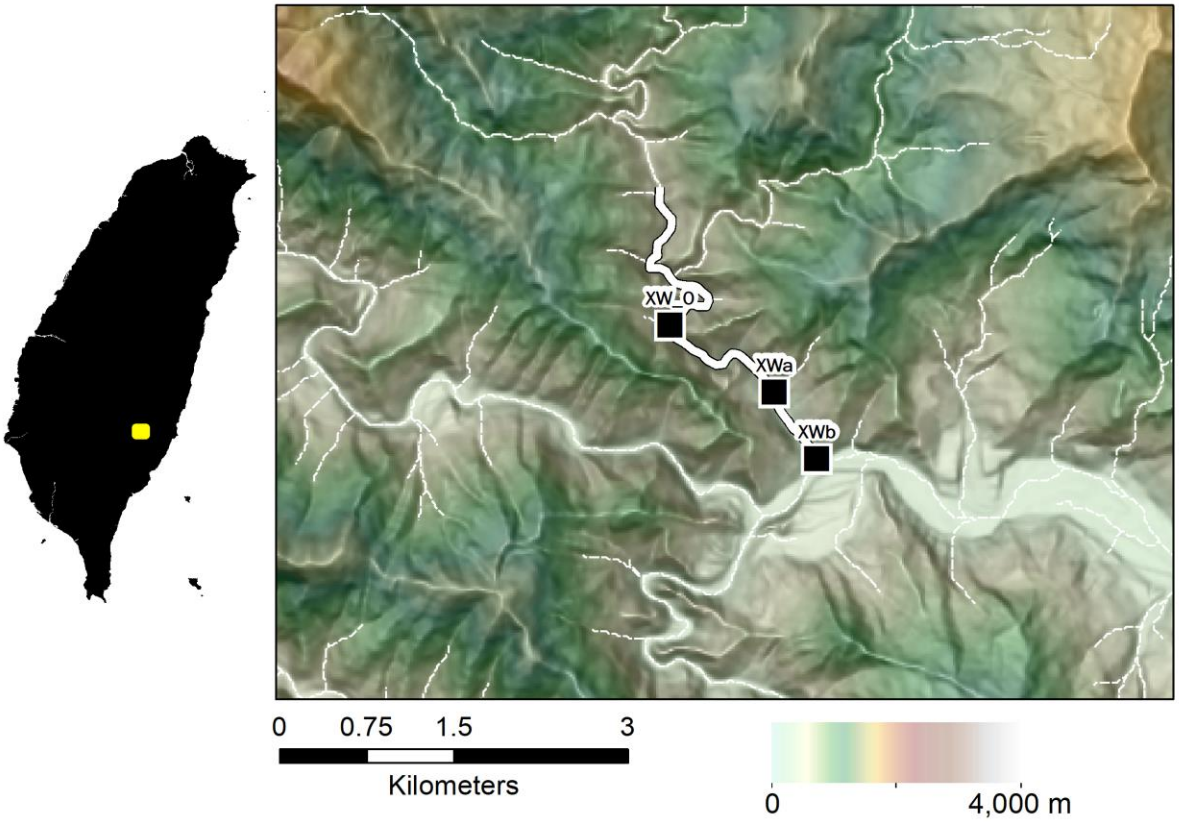

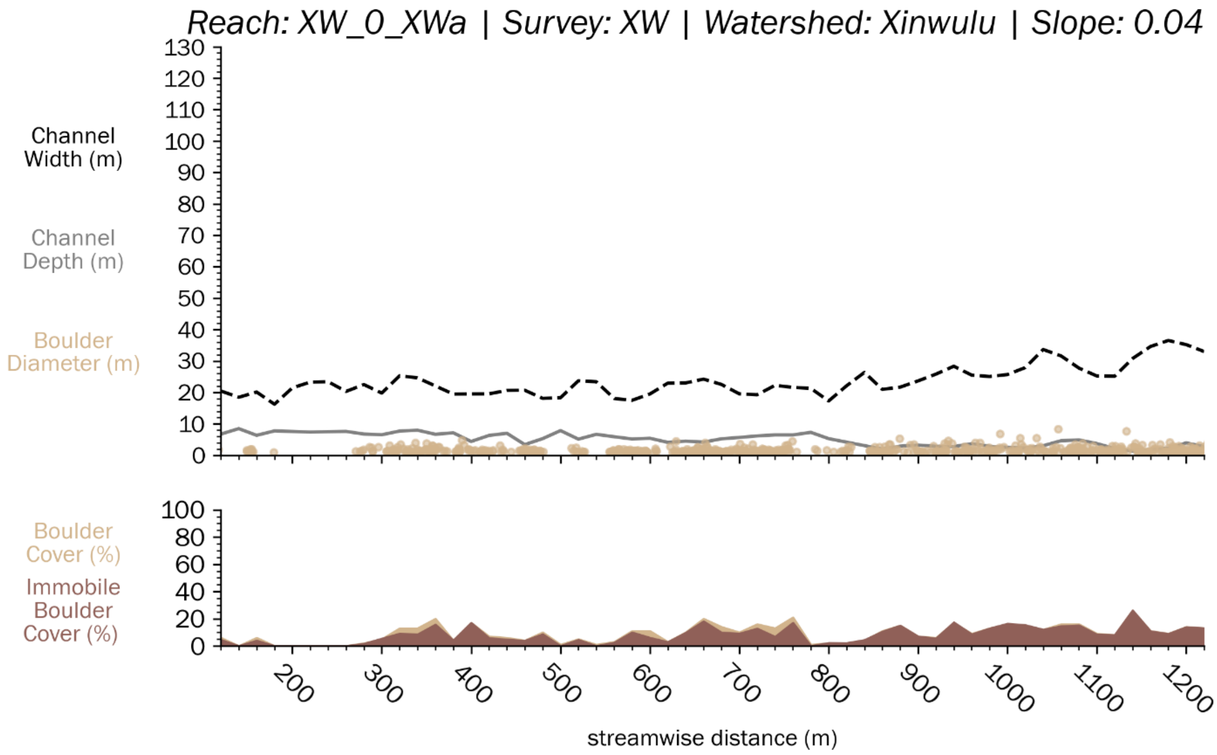

Reach: XW_0_XWa | Survey: XW | Watershed: Xinwulu | Slope: 0.04
Channel Width (m)
Channel Depth (m)
Boulder Diameter (m)
130
120
110
100
90
80
70
60
50
40
30
20
10
0
Boulder Cover (%)
Immobile Boulder Cover (%)
100
80
60
40
20
0
200
300
400
500
600
700
800
900
1000
1100
1200
streamwise distance (m)

Site: Lele

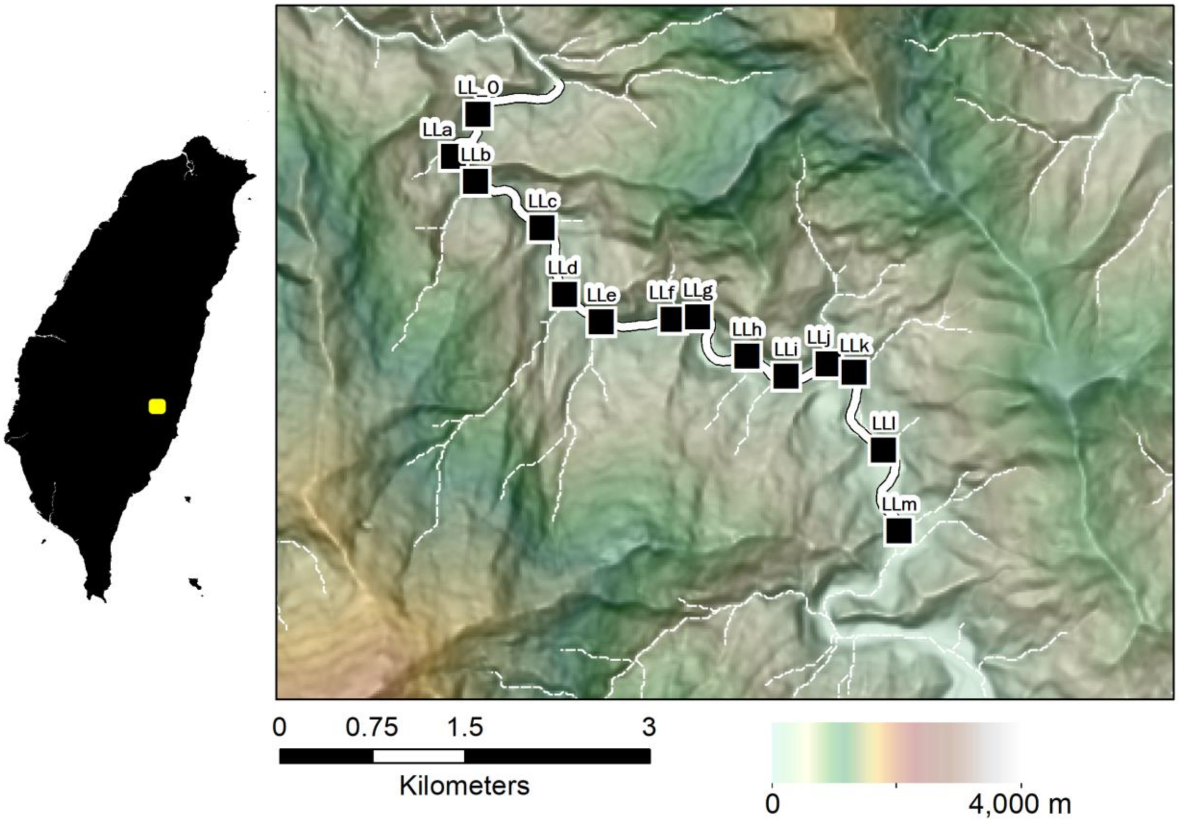
LL_0
LLa
LLb
LLc
LLd
LLe
LLf
LLg
LLh
LLi
LLj
LLk
LLl
LLm
0
0.75
1.5
3
Kilometers
0
4,000 m

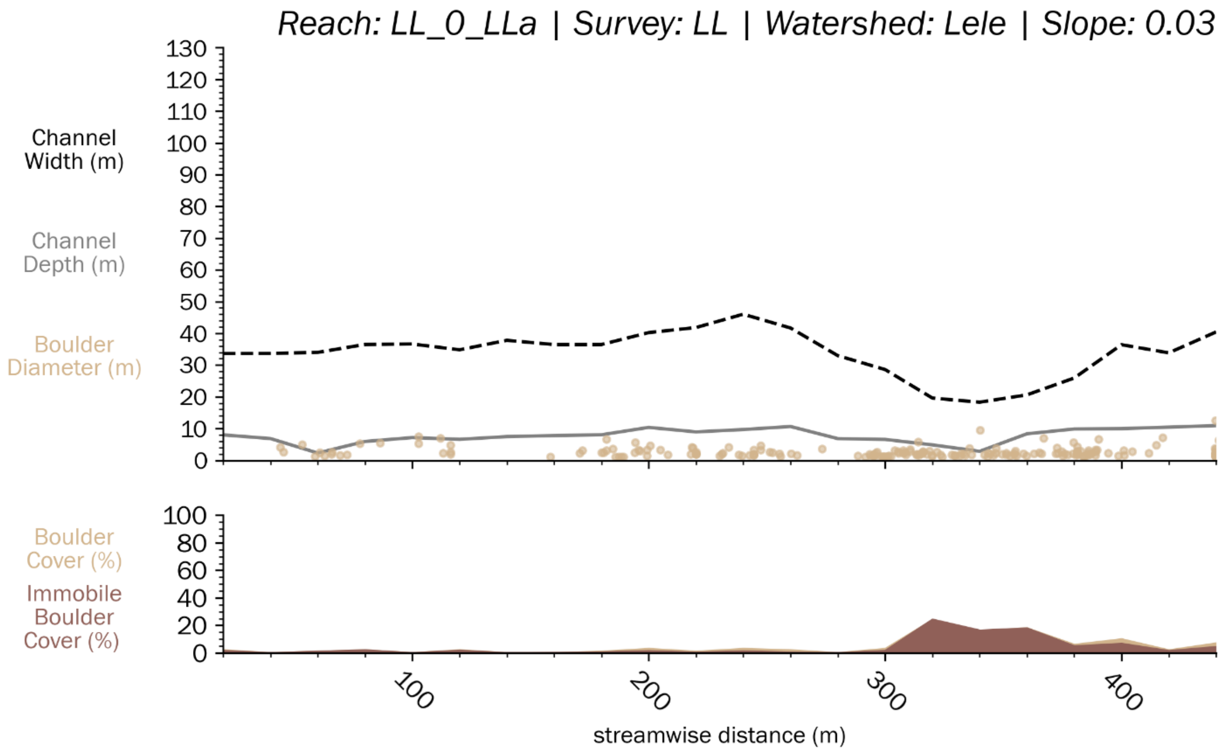
Reach: LL_0_LLa | Survey: LL | Watershed: Lele | Slope: 0.03
Channel Width (m)
Channel Depth (m)
Boulder Diameter (m)
Boulder Cover (%)
Immobile Boulder Cover (%)
streamwise distance (m)

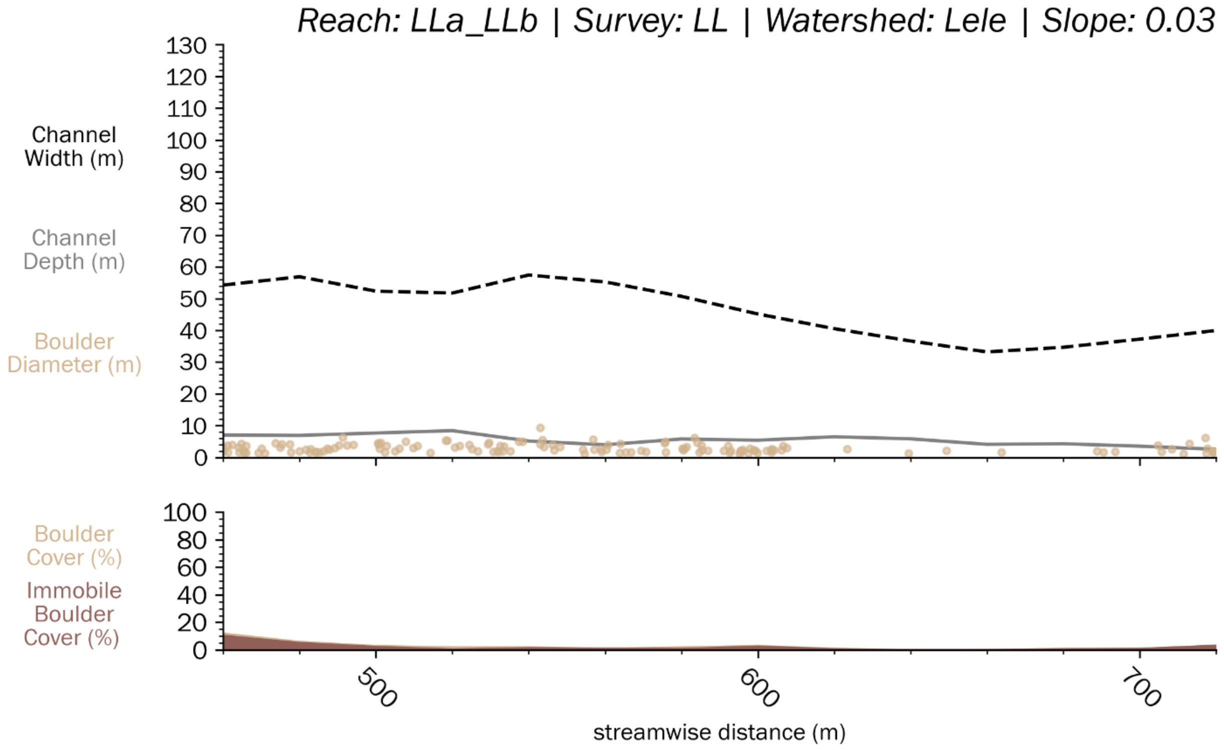
Reach: LLa_LLb | Survey: LL | Watershed: Lele | Slope: 0.03
Channel Width (m)
Channel Depth (m)
Boulder Diameter (m)
Boulder Cover (%)
Immobile Boulder Cover (%)
streamwise distance (m)

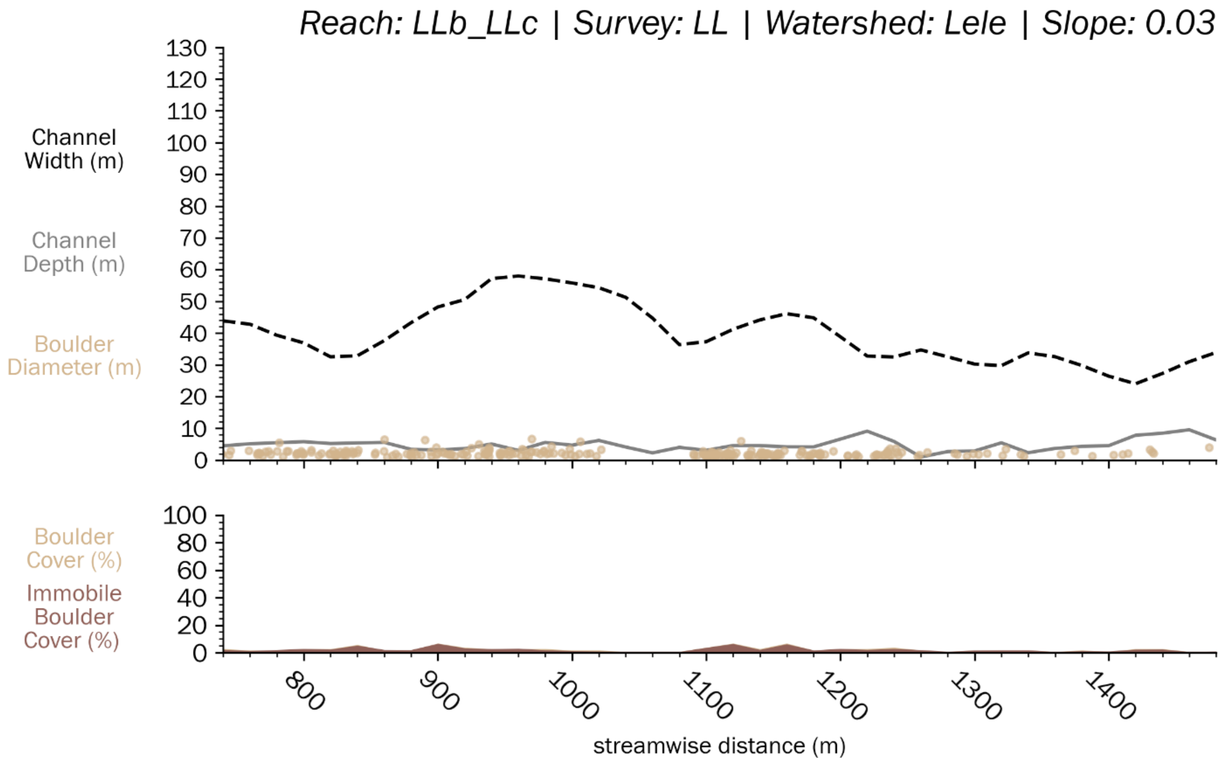
Reach: LLb_LLc | Survey: LL | Watershed: Lele | Slope: 0.03
Channel Width (m)
Channel Depth (m)
Boulder Diameter (m)
Boulder Cover (%)
Immobile Boulder Cover (%)
130
120
110
100
90
80
70
60
50
40
30
20
10
0
100
80
60
40
20
0
800
900
1000
1100
1200
1300
1400
streamwise distance (m)

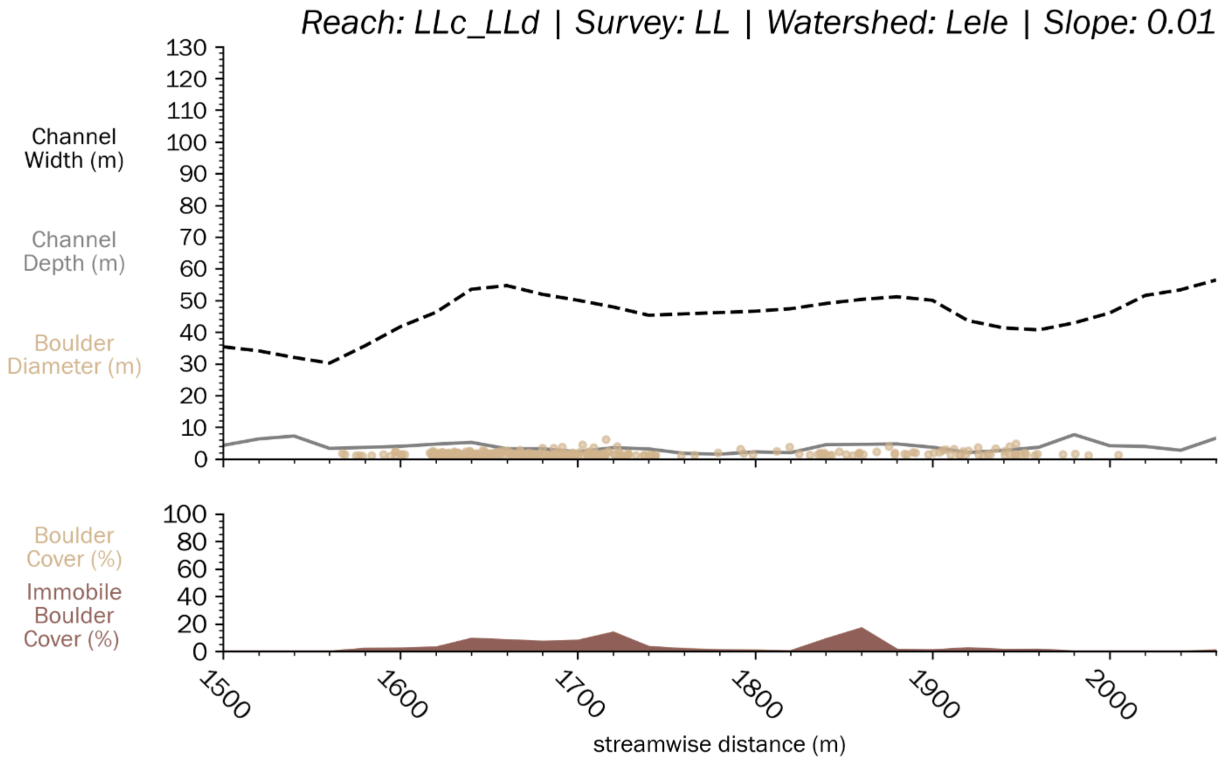
Reach: LLc_LLd | Survey: LL | Watershed: Lele | Slope: 0.01
Channel Width (m)
Channel Depth (m)
Boulder Diameter (m)
Boulder Cover (%)
Immobile Boulder Cover (%)
130
120
110
100
90
80
70
60
50
40
30
20
10
0
100
80
60
40
20
0
1500
1600
1700
1800
1900
2000
streamwise distance (m)

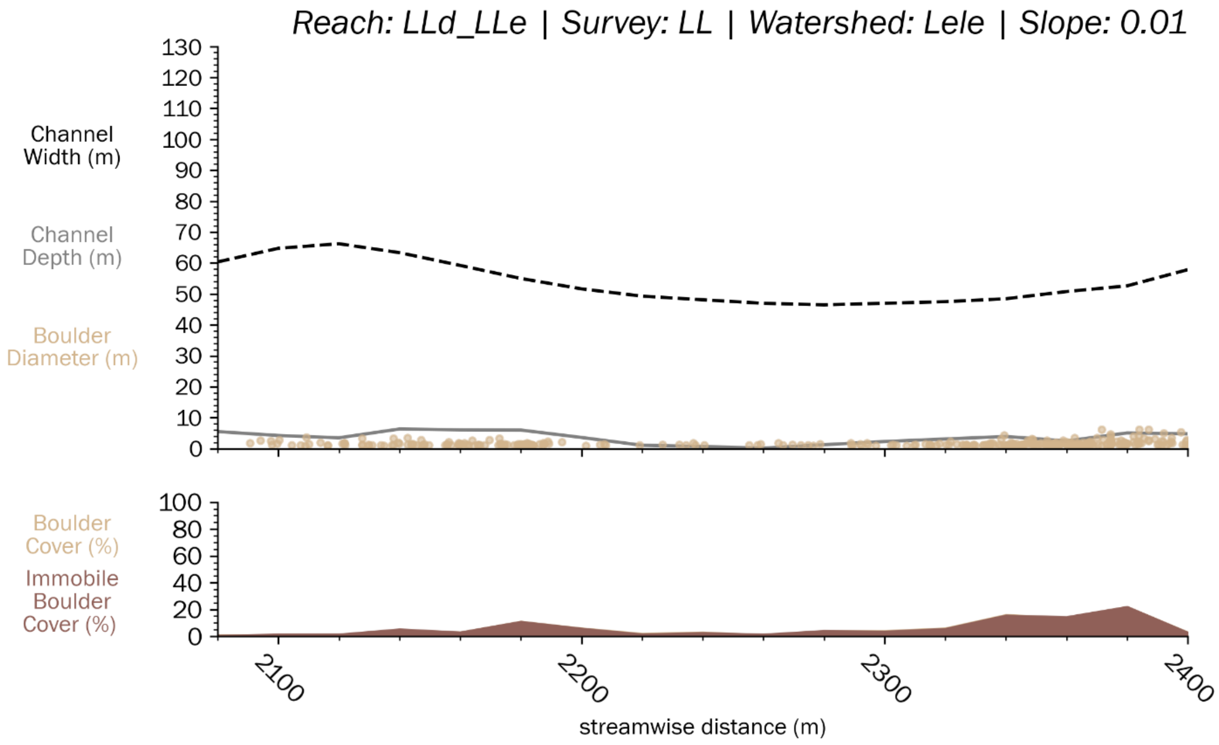

Reach: LLd_LLe | Survey: LL | Watershed: Lele | Slope: 0.01
Channel Width (m)
Channel Depth (m)
Boulder Diameter (m)
130
120
110
100
90
80
70
60
50
40
30
20
10
0
Boulder Cover (%)
Immobile Boulder Cover (%)
100
80
60
40
20
0
2100
2200
2300
2400
streamwise distance (m)

Region: North

Site: Tacijili (TC), Tianxiang (TX), Lushui (LS), Liwu basin

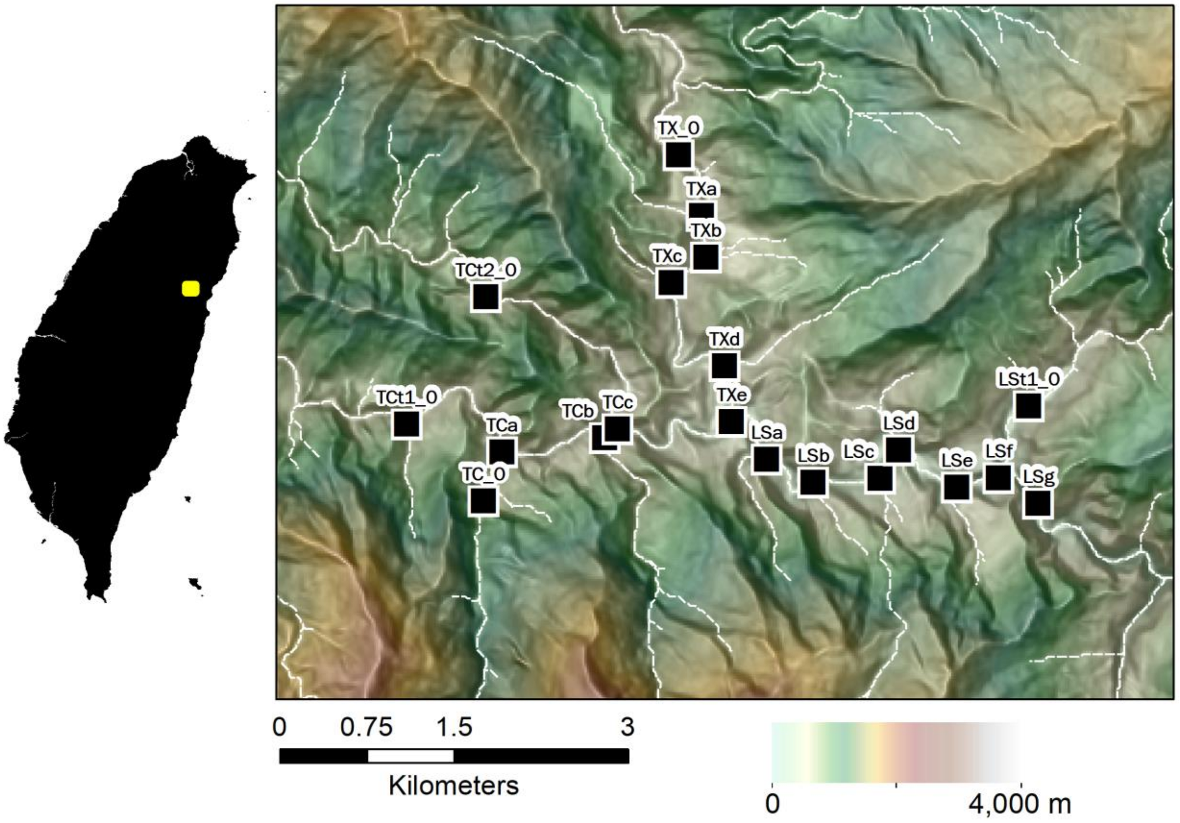

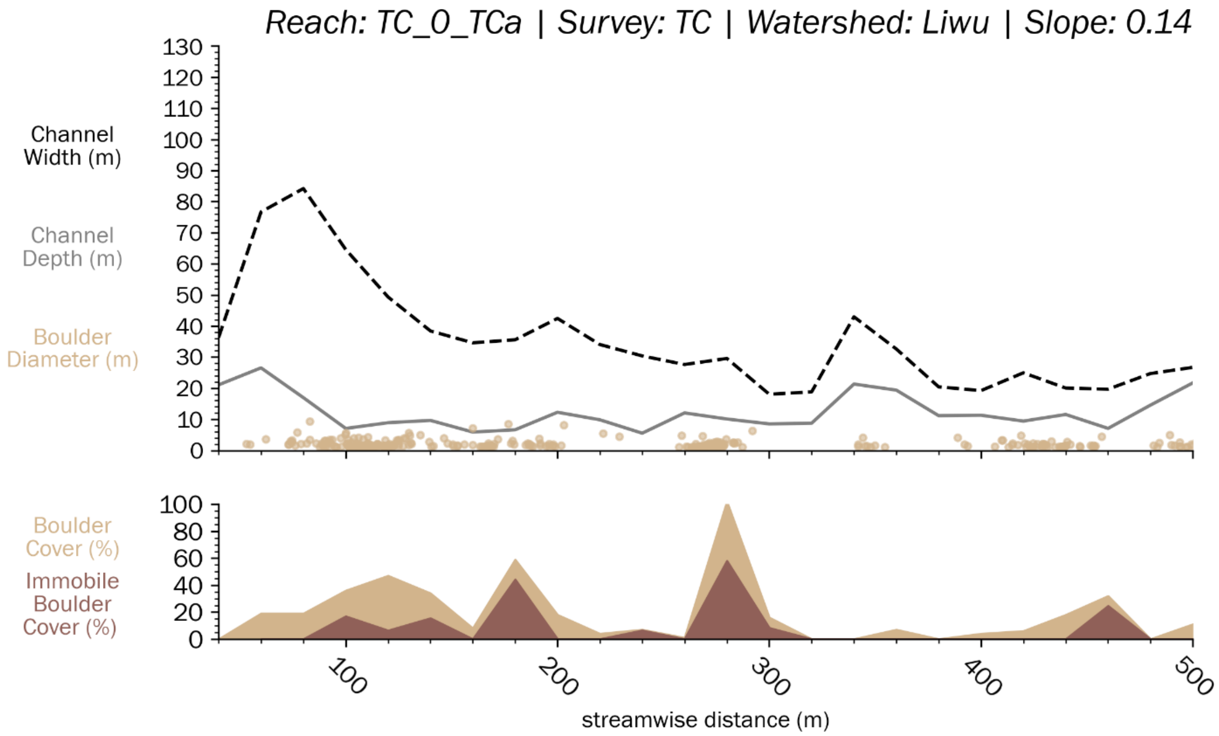

Reach: TC_0_TCa | Survey: TC | Watershed: Liwu | Slope: 0.14
Channel Width (m)
Channel Depth (m)
Boulder Diameter (m)
Boulder Cover (%)
Immobile Boulder Cover (%)
streamwise distance (m)

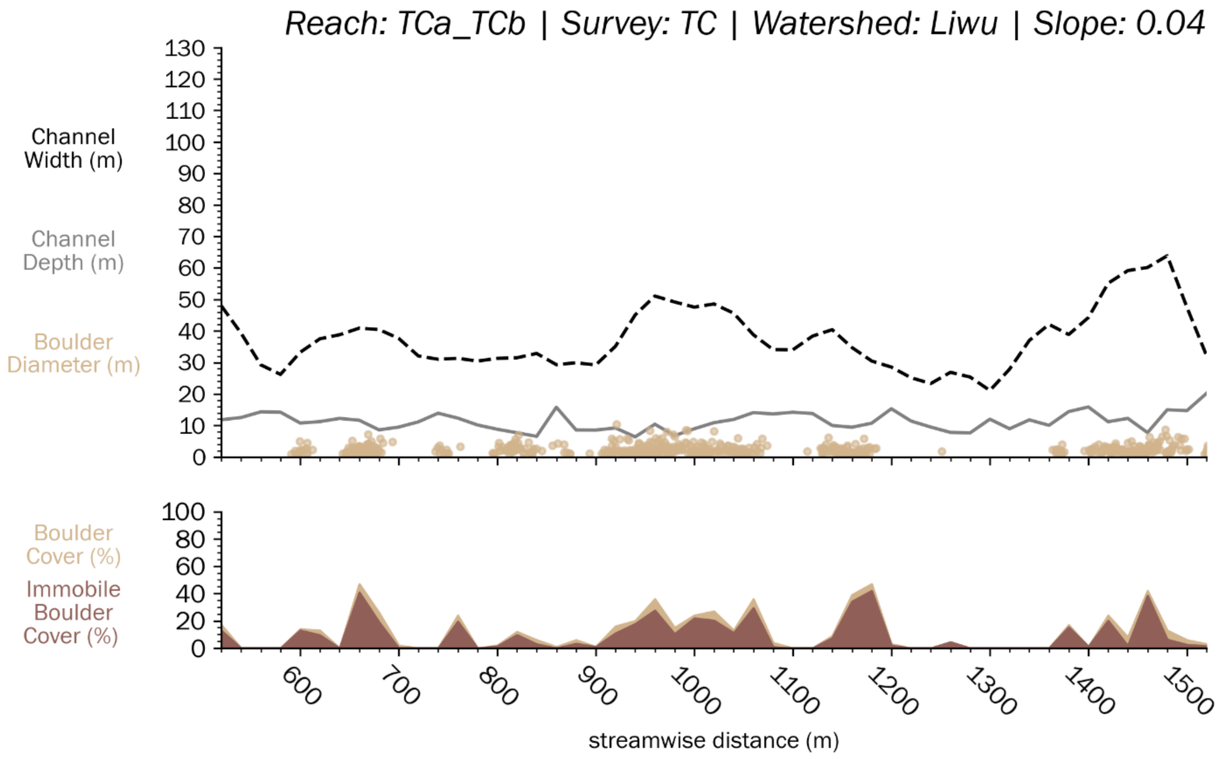

Reach: TCa_TCb | Survey: TC | Watershed: Liwu | Slope: 0.04
Channel Width (m)
Channel Depth (m)
Boulder Diameter (m)
Boulder Cover (%)
Immobile Boulder Cover (%)
streamwise distance (m)

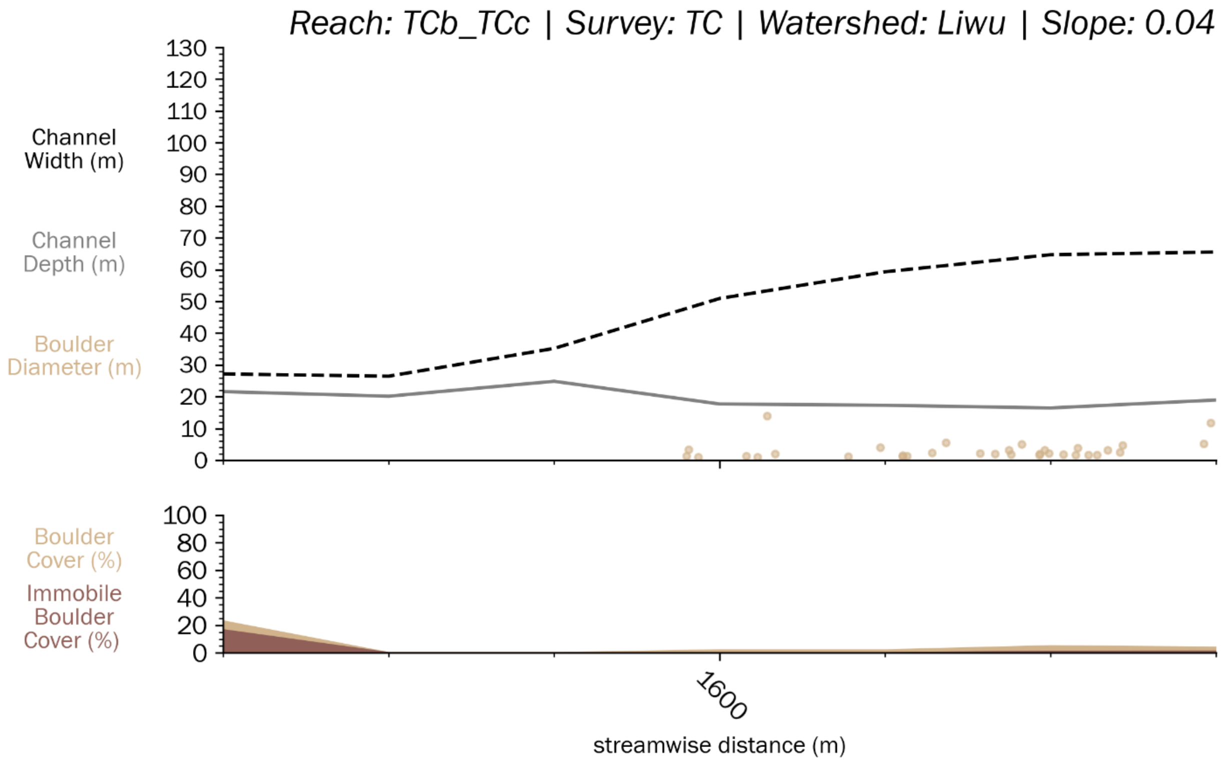
Reach: TCb_TCc | Survey: TC | Watershed: Liwu | Slope: 0.04
Channel Width (m)
Channel Depth (m)
Boulder Diameter (m)
130
120
110
100
90
80
70
60
50
40
30
20
10
0
Boulder Cover (%)
Immobile Boulder Cover (%)
100
80
60
40
20
0
1600
streamwise distance (m)

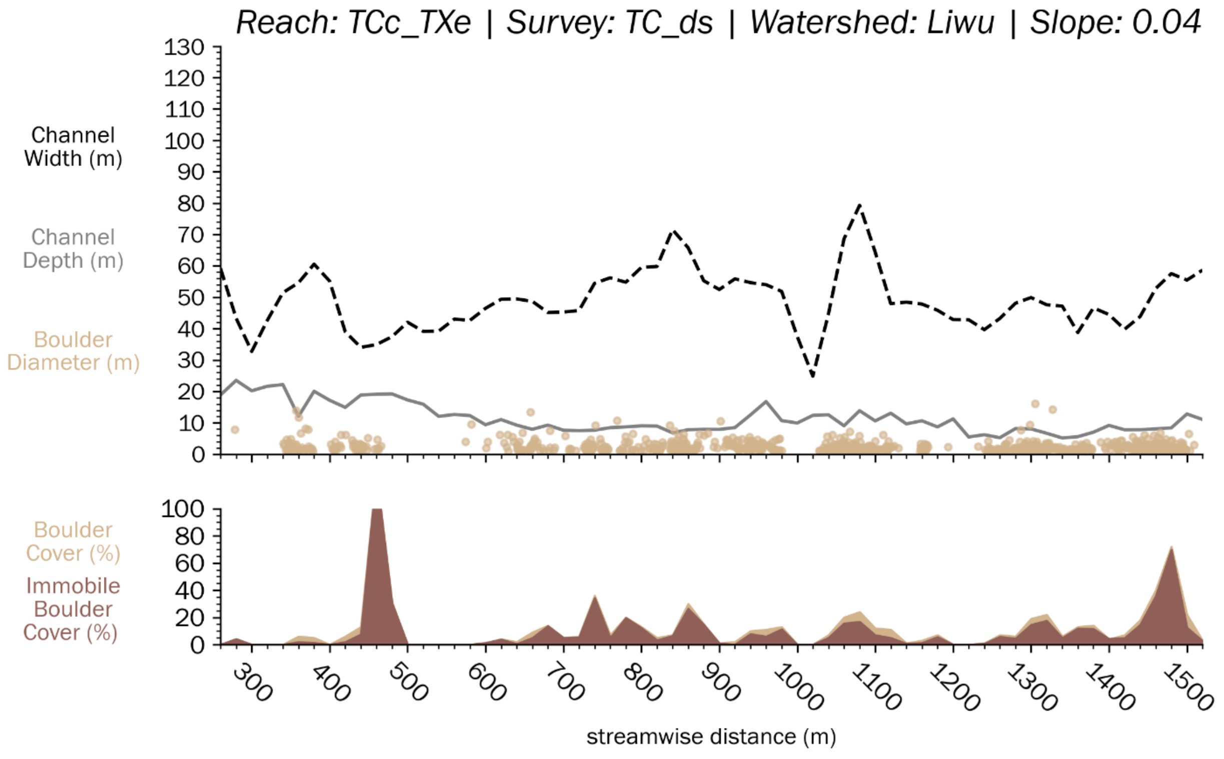
Reach: TCc_TXe | Survey: TC_ds | Watershed: Liwu | Slope: 0.04
Channel Width (m)
Channel Depth (m)
Boulder Diameter (m)
130
120
110
100
90
80
70
60
50
40
30
20
10
0
Boulder Cover (%)
Immobile Boulder Cover (%)
100
80
60
40
20
0
300
400
500
600
700
800
900
1000
1100
1200
1300
1400
1500
streamwise distance (m)

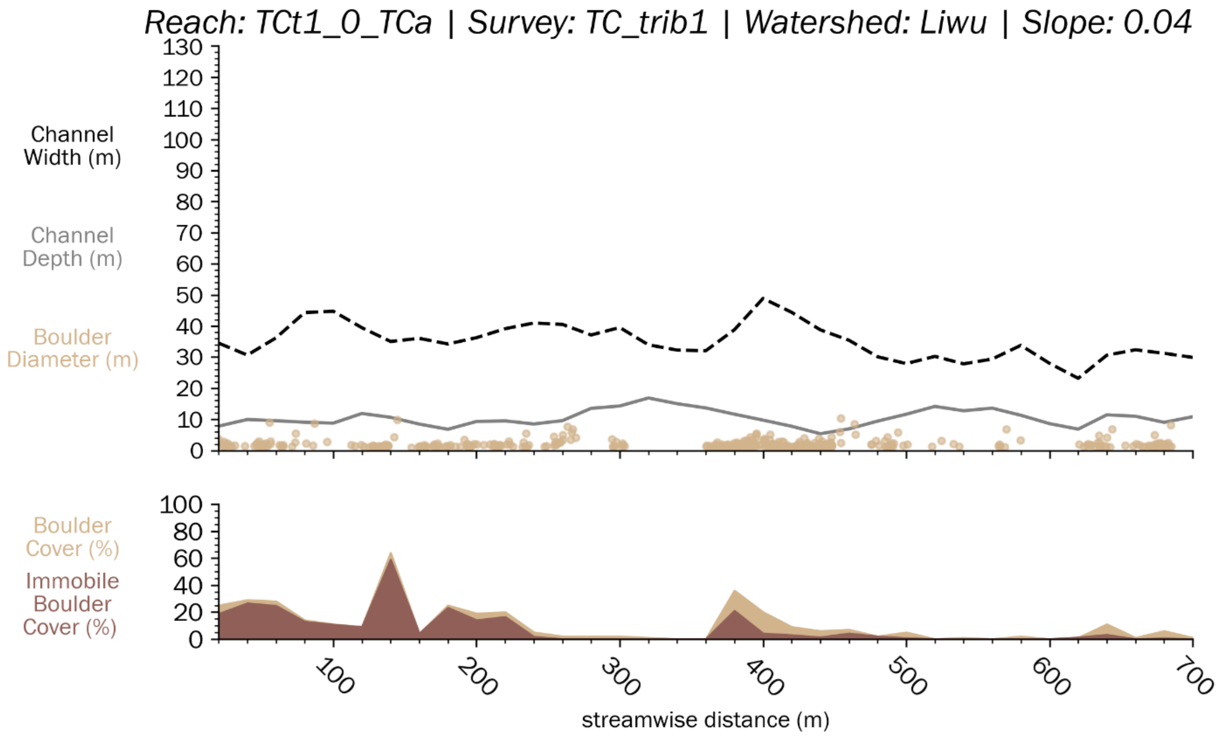
Reach: TCt1_0_TCa | Survey: TC_trib1 | Watershed: Liwu | Slope: 0.04
Channel Width (m)
Channel Depth (m)
Boulder Diameter (m)
130
120
110
100
90
80
70
60
50
40
30
20
10
0
Boulder Cover (%)
Immobile Boulder Cover (%)
100
80
60
40
20
0
100
200
300
400
500
600
700
streamwise distance (m)

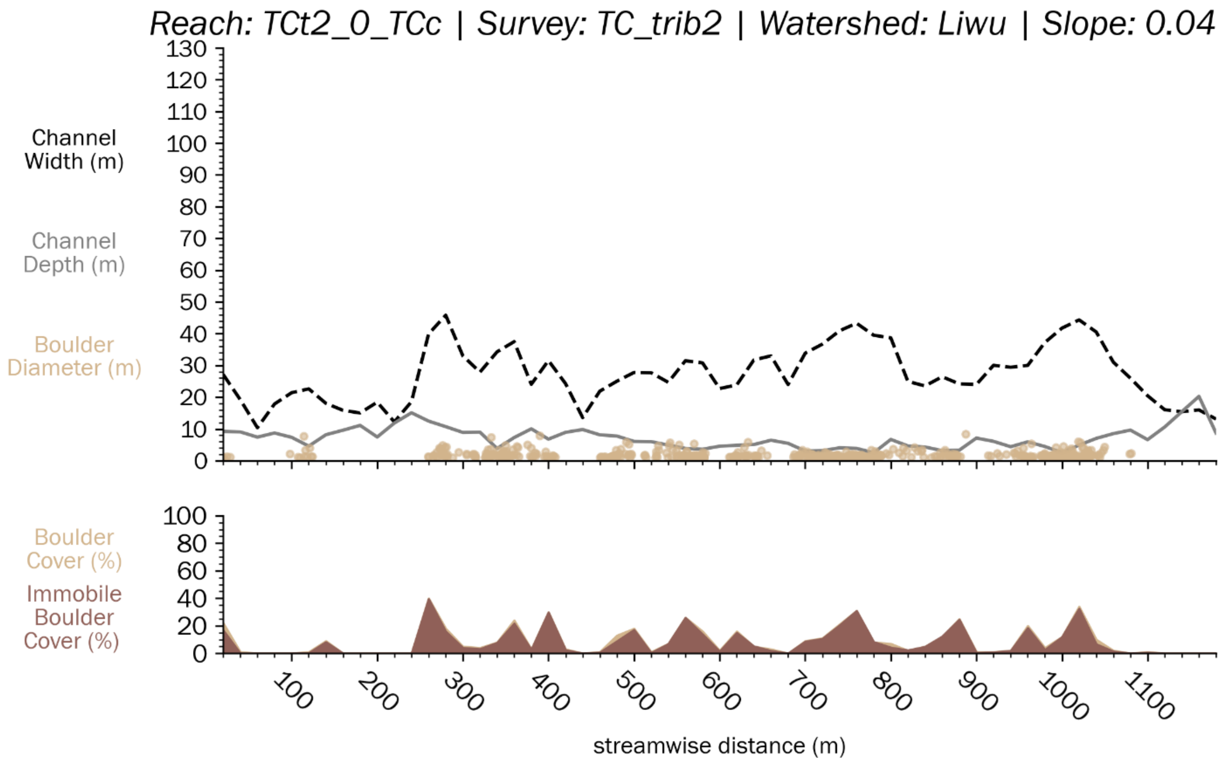
Reach: TCt2_0_TCc | Survey: TC_trib2 | Watershed: Liwu | Slope: 0.04
Channel Width (m)
Channel Depth (m)
Boulder Diameter (m)
130
120
110
100
90
80
70
60
50
40
30
20
10
0
Boulder Cover (%)
Immobile Boulder Cover (%)
100
80
60
40
20
0
100
200
300
400
500
600
700
800
900
1000
1100
streamwise distance (m)

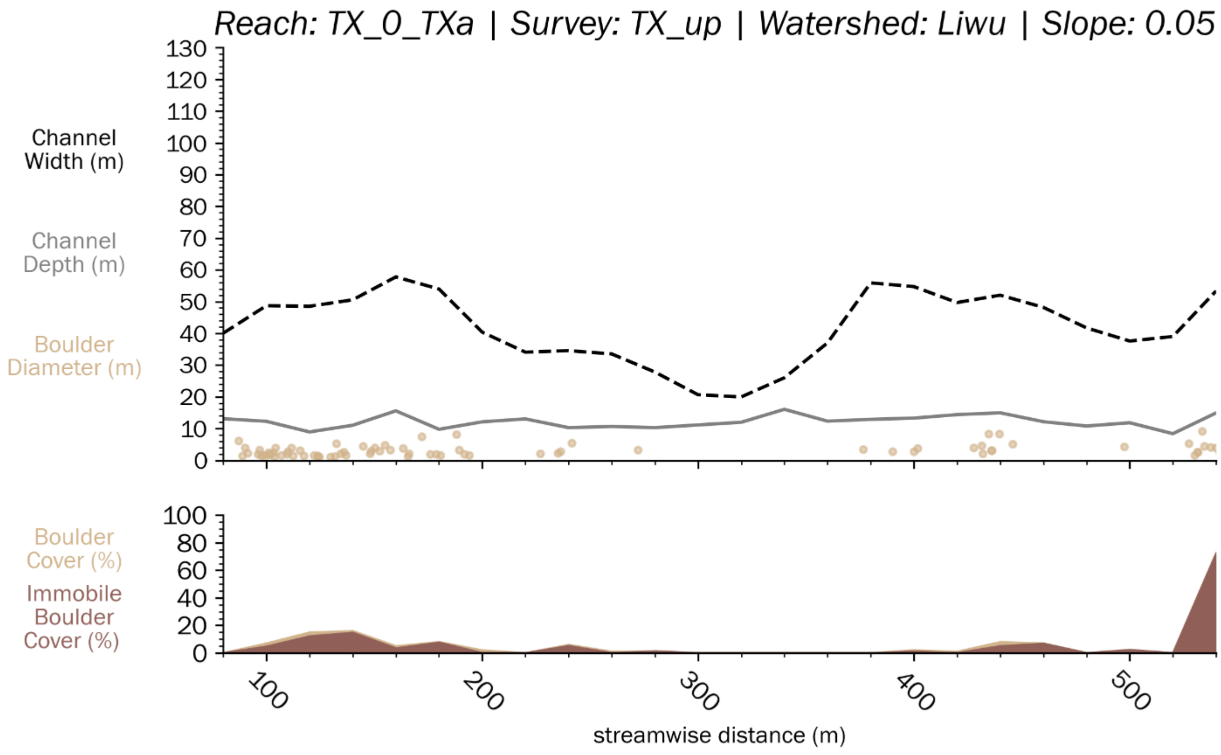
Reach: TX_0_TXa | Survey: TX_up | Watershed: Liwu | Slope: 0.05
Channel Width (m)
Channel Depth (m)
Boulder Diameter (m)
Boulder Cover (%)
Immobile Boulder Cover (%)
130
120
110
100
90
80
70
60
50
40
30
20
10
0
100
80
60
40
20
0
100
200
300
400
500
streamwise distance (m)

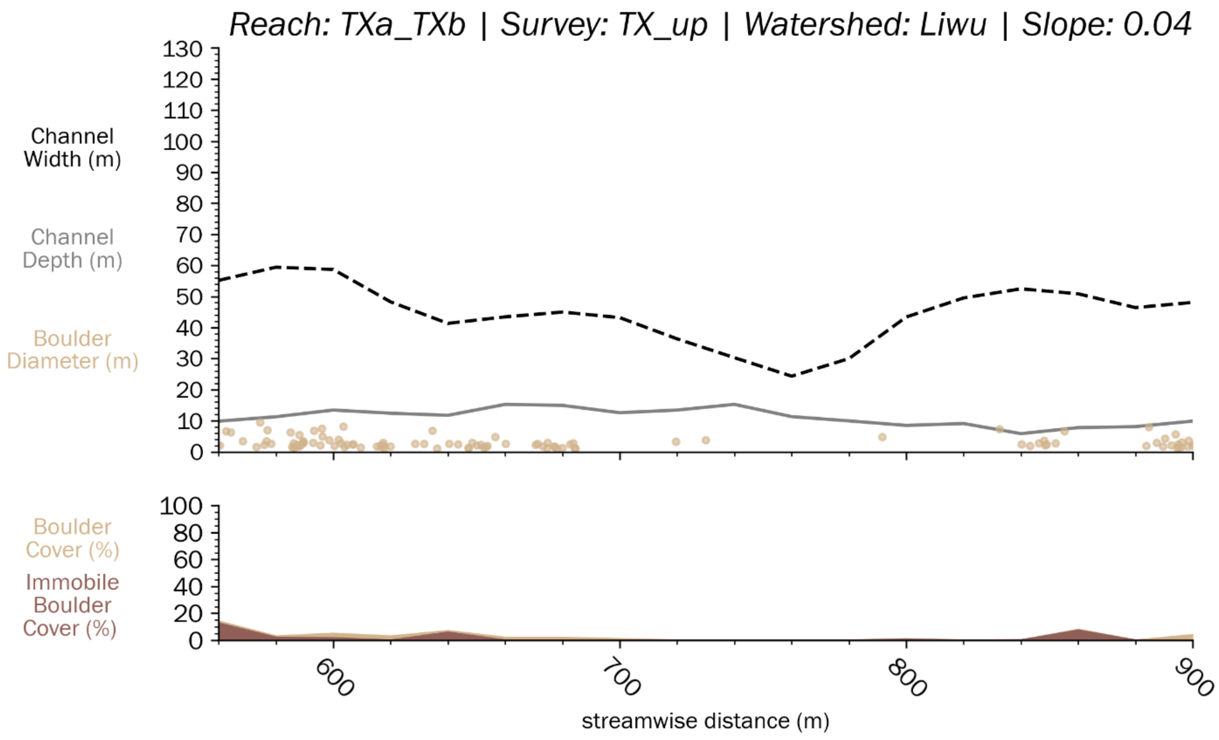
Reach: TXa_TXb | Survey: TX_up | Watershed: Liwu | Slope: 0.04
Channel Width (m)
Channel Depth (m)
Boulder Diameter (m)
Boulder Cover (%)
Immobile Boulder Cover (%)
130
120
110
100
90
80
70
60
50
40
30
20
10
0
100
80
60
40
20
0
600
700
800
900
streamwise distance (m)

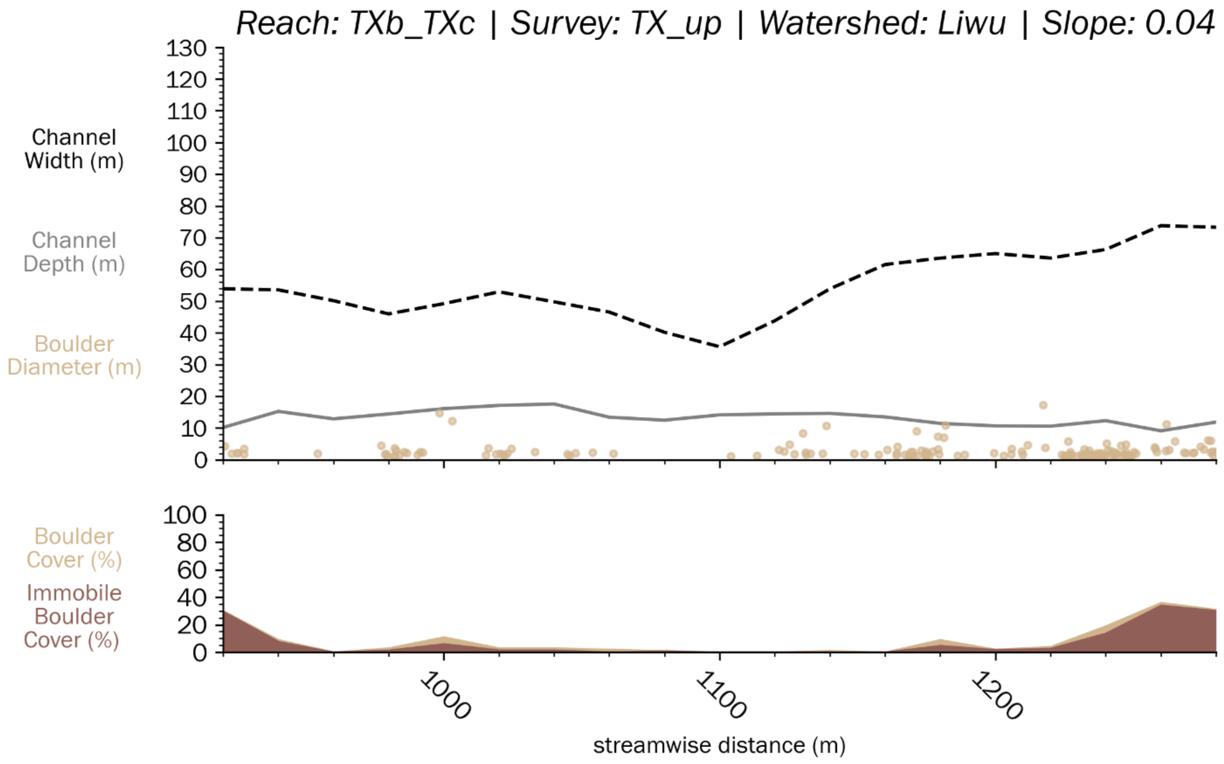

Reach: TXb_TXc | Survey: TX_up | Watershed: Liwu | Slope: 0.04
Channel Width (m)
Channel Depth (m)
Boulder Diameter (m)
Boulder Cover (%)
Immobile Boulder Cover (%)
1000
1100
1200
streamwise distance (m)

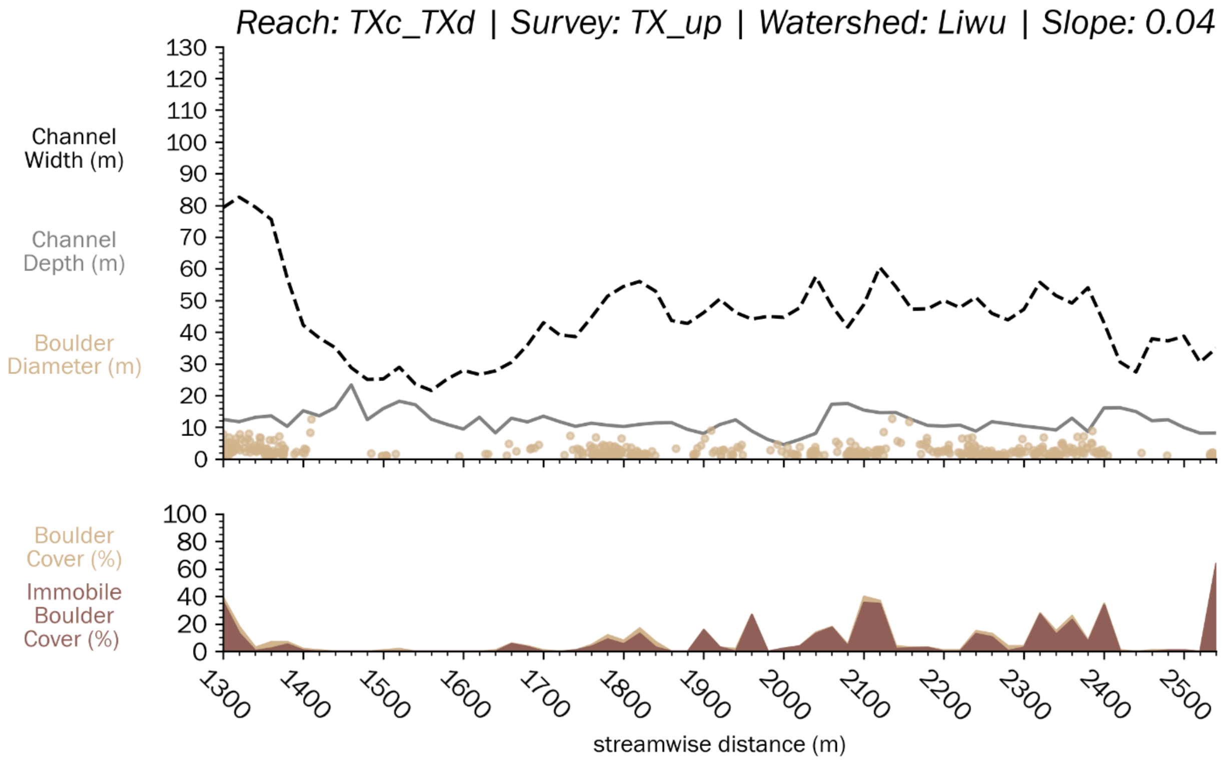

Reach: TXc_TXd | Survey: TX_up | Watershed: Liwu | Slope: 0.04
Channel Width (m)
Channel Depth (m)
Boulder Diameter (m)
Boulder Cover (%)
Immobile Boulder Cover (%)
1300
1400
1500
1600
1700
1800
1900
2000
2100
2200
2300
2400
2500
streamwise distance (m)

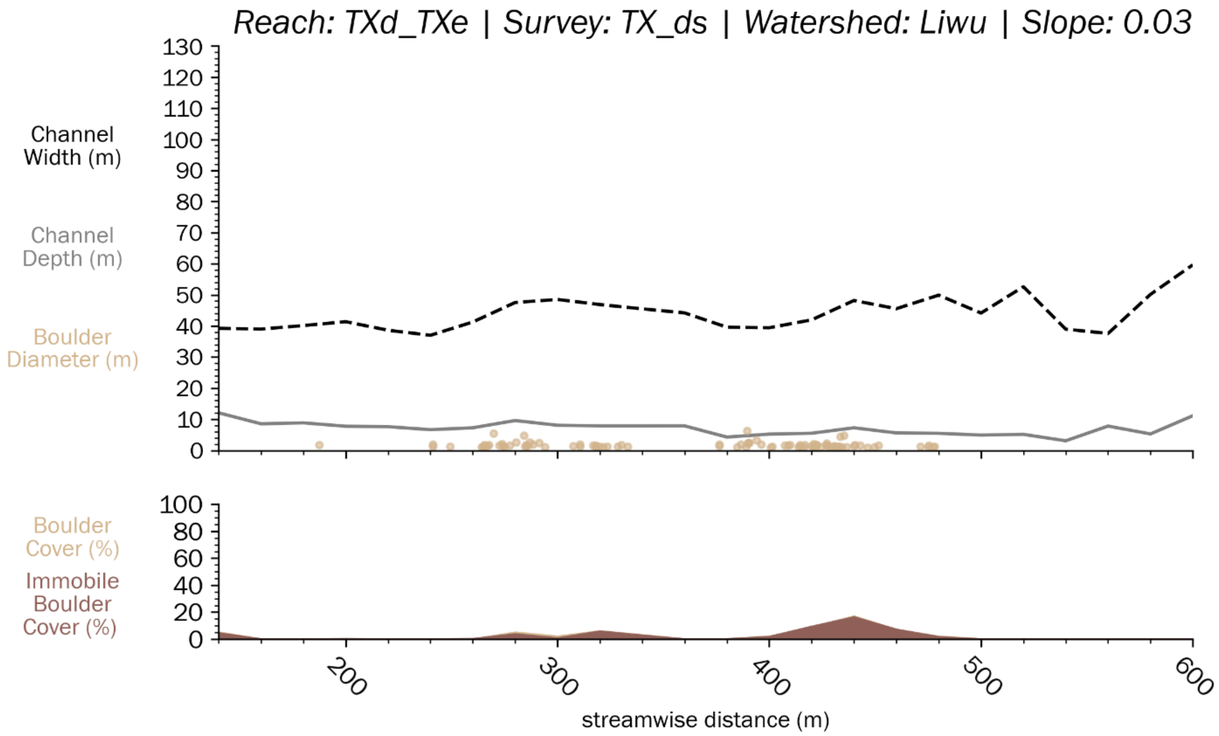

Reach: TXd_TXe | Survey: TX_ds | Watershed: Liwu | Slope: 0.03
Channel Width (m)
Channel Depth (m)
Boulder Diameter (m)
Boulder Cover (%)
Immobile Boulder Cover (%)
130
120
110
100
90
80
70
60
50
40
30
20
10
0
100
80
60
40
20
0
200
300
400
500
600
streamwise distance (m)

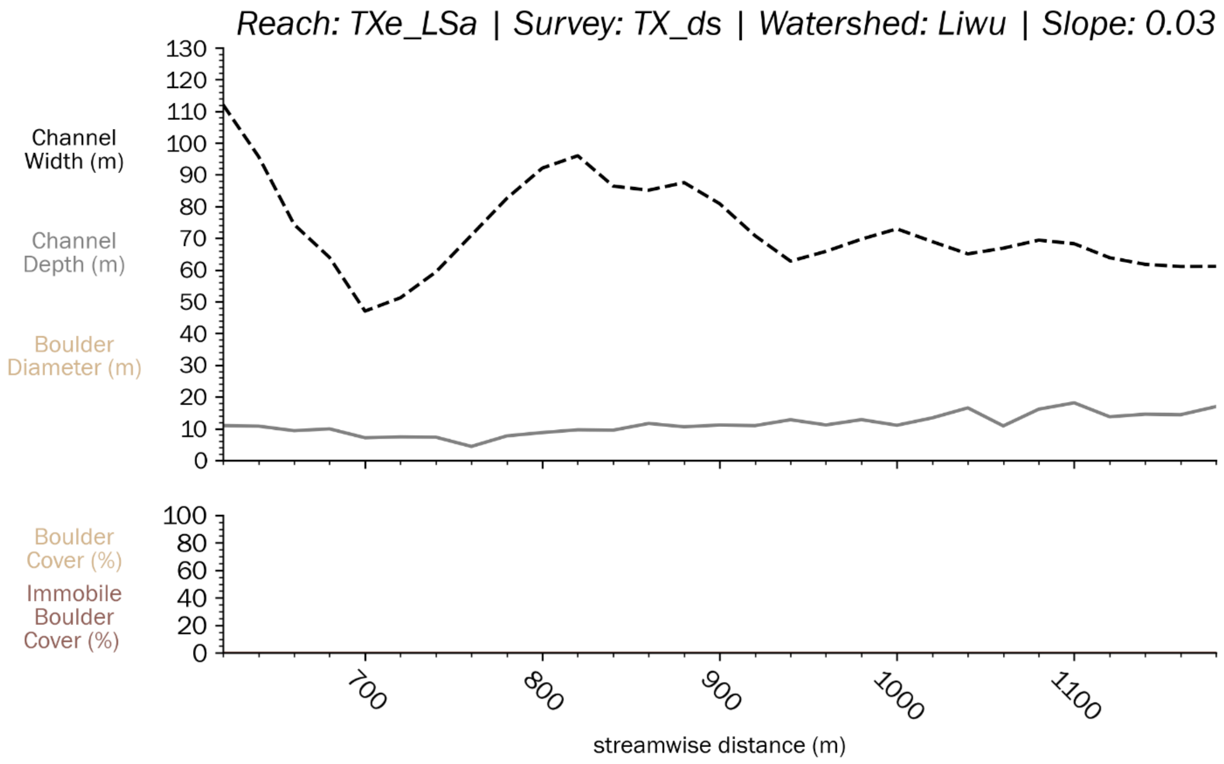

Reach: TXe_LSa | Survey: TX_ds | Watershed: Liwu | Slope: 0.03
Channel Width (m)
Channel Depth (m)
Boulder Diameter (m)
Boulder Cover (%)
Immobile Boulder Cover (%)
130
120
110
100
90
80
70
60
50
40
30
20
10
0
100
80
60
40
20
0
700
800
900
1000
1100
streamwise distance (m)

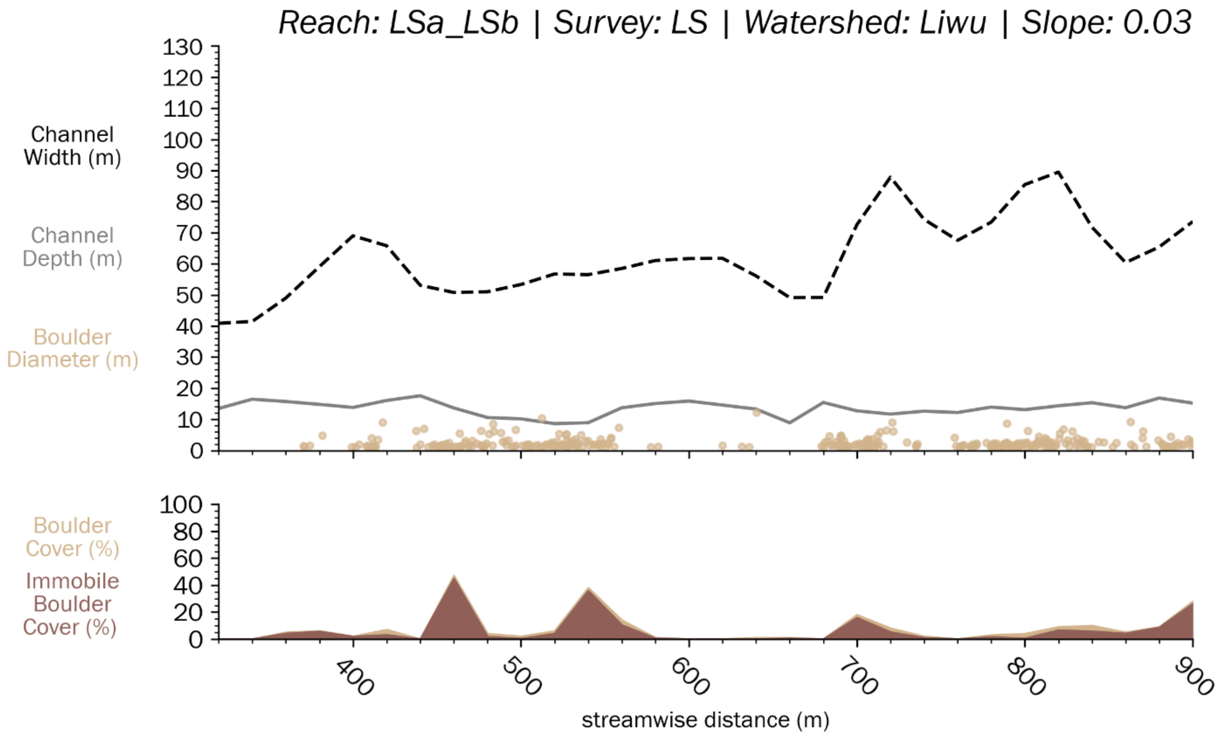
Reach: LSa_LSb | Survey: LS | Watershed: Liwu | Slope: 0.03
Channel Width (m)
Channel Depth (m)
Boulder Diameter (m)
Boulder Cover (%)
Immobile Boulder Cover (%)
streamwise distance (m)

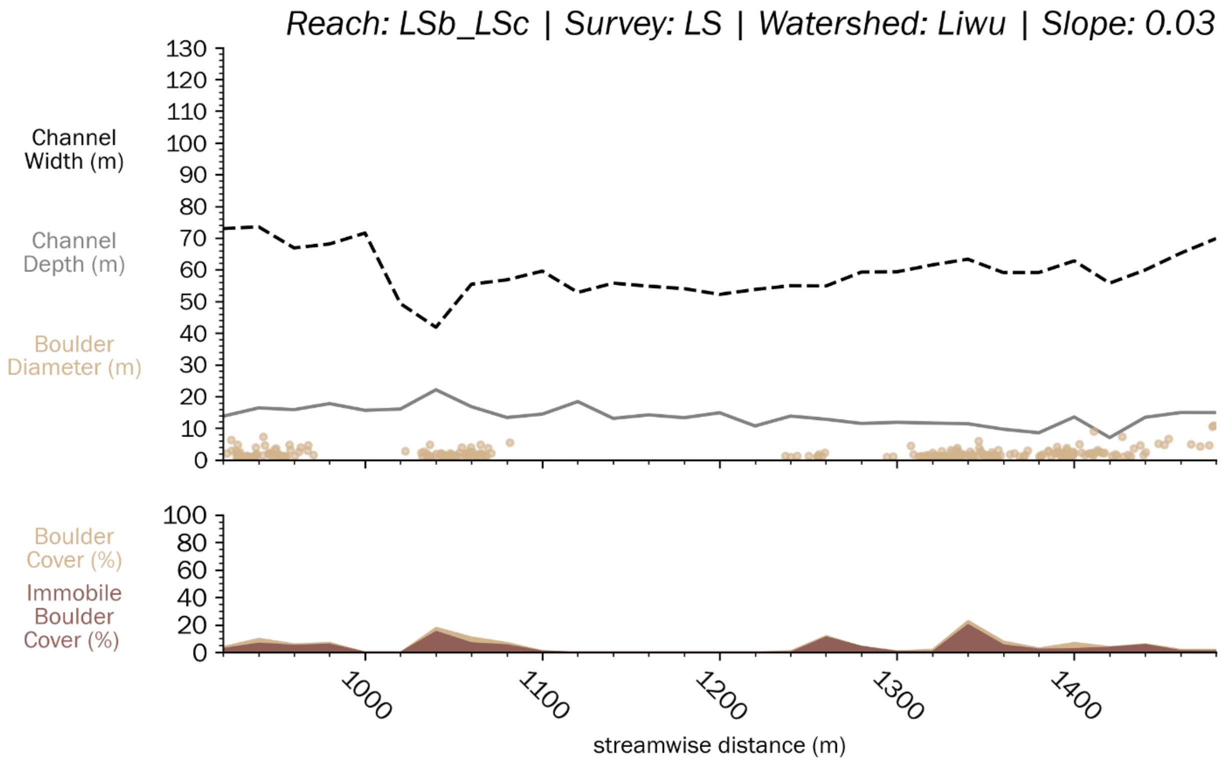
Reach: LSb_LSc | Survey: LS | Watershed: Liwu | Slope: 0.03
Channel Width (m)
Channel Depth (m)
Boulder Diameter (m)
Boulder Cover (%)
Immobile Boulder Cover (%)
streamwise distance (m)

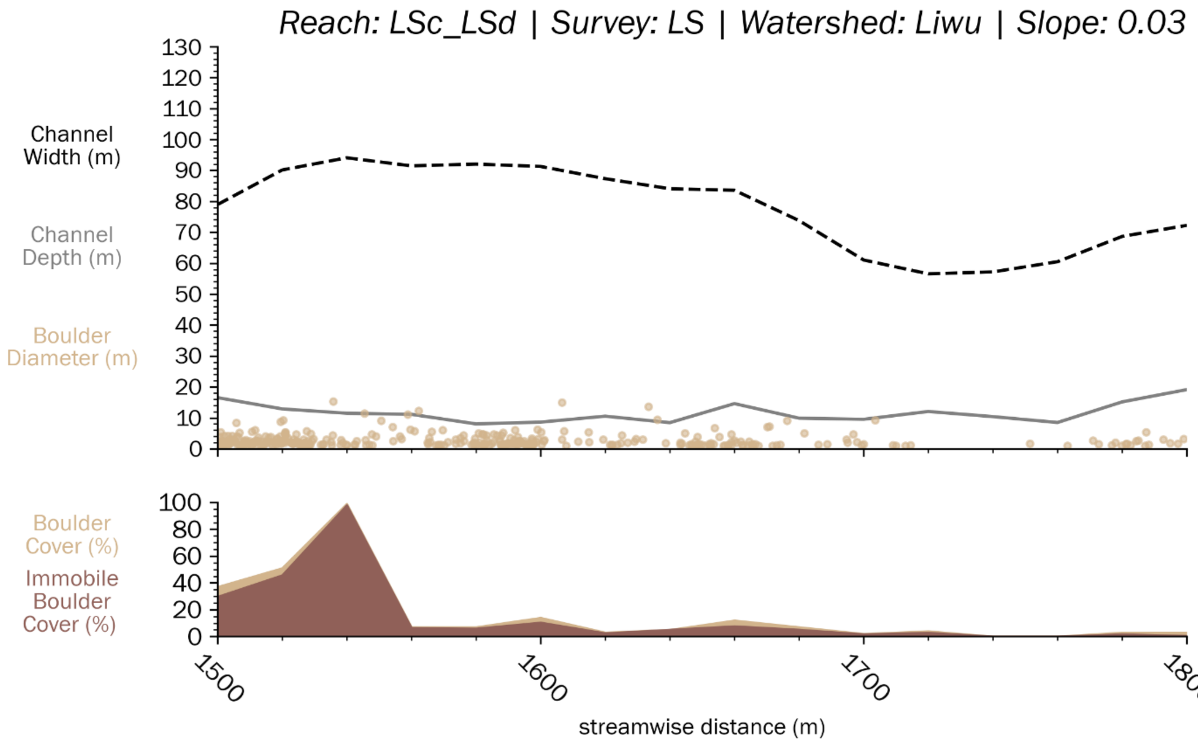

Reach: LSc_LSd | Survey: LS | Watershed: Liwu | Slope: 0.03
Channel Width (m)
Channel Depth (m)
Boulder Diameter (m)
Boulder Cover (%)
Immobile Boulder Cover (%)
streamwise distance (m)

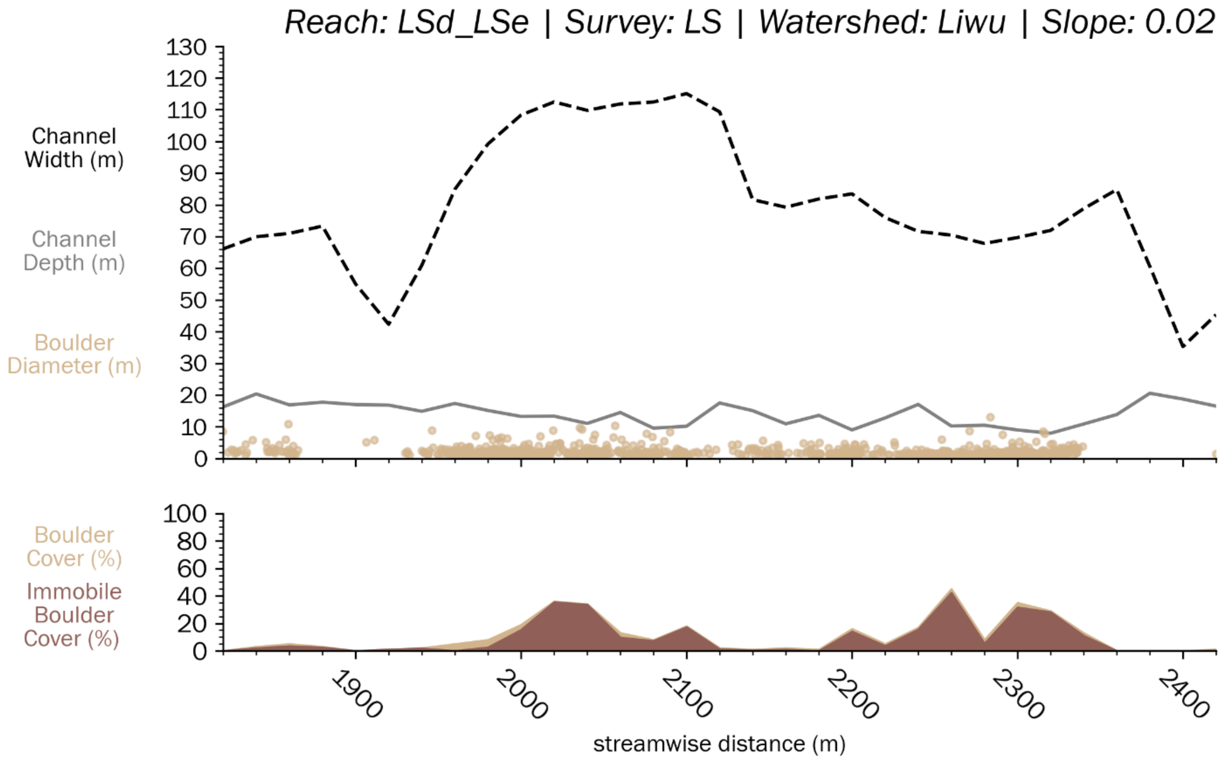

Reach: LSd_LSe | Survey: LS | Watershed: Liwu | Slope: 0.02
Channel Width (m)
Channel Depth (m)
Boulder Diameter (m)
Boulder Cover (%)
Immobile Boulder Cover (%)
streamwise distance (m)

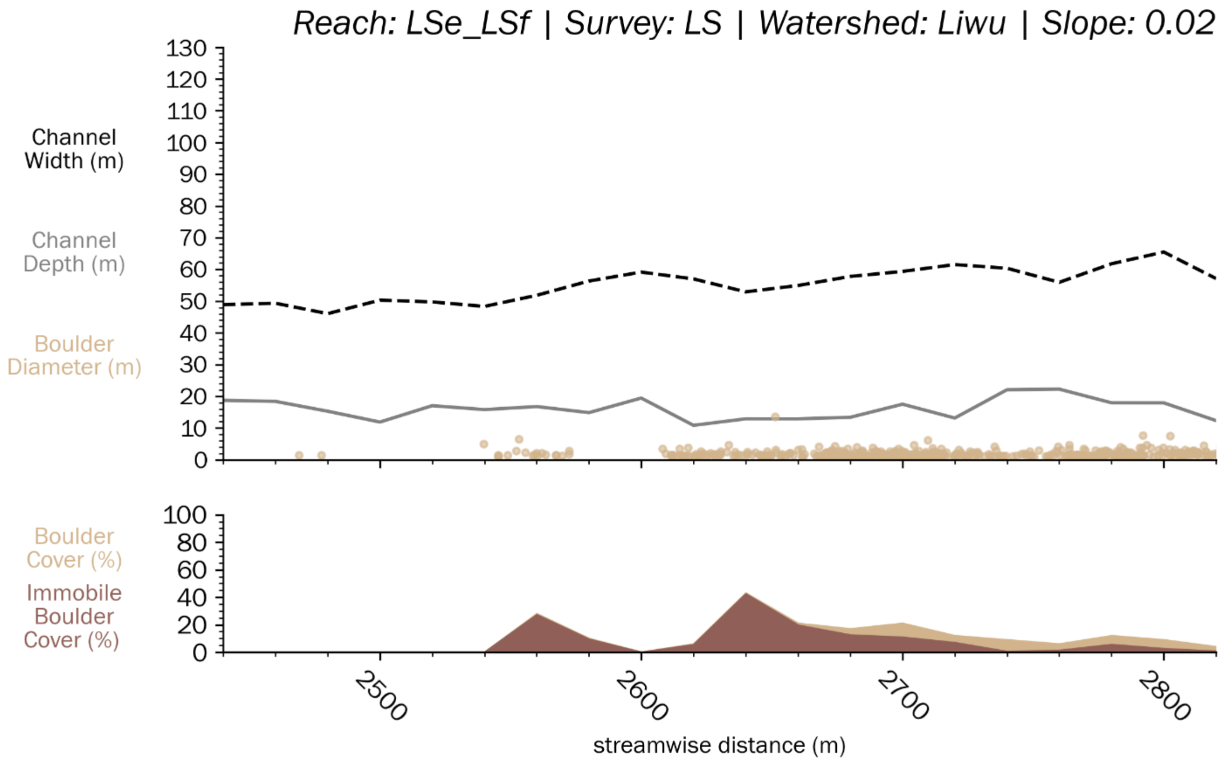

Reach: LSe_LSf | Survey: LS | Watershed: Liwu | Slope: 0.02
Channel Width (m)
Channel Depth (m)
Boulder Diameter (m)
Boulder Cover (%)
Immobile Boulder Cover (%)
130
120
110
100
90
80
70
60
50
40
30
20
10
0
100
80
60
40
20
0
2500
2600
2700
2800
streamwise distance (m)

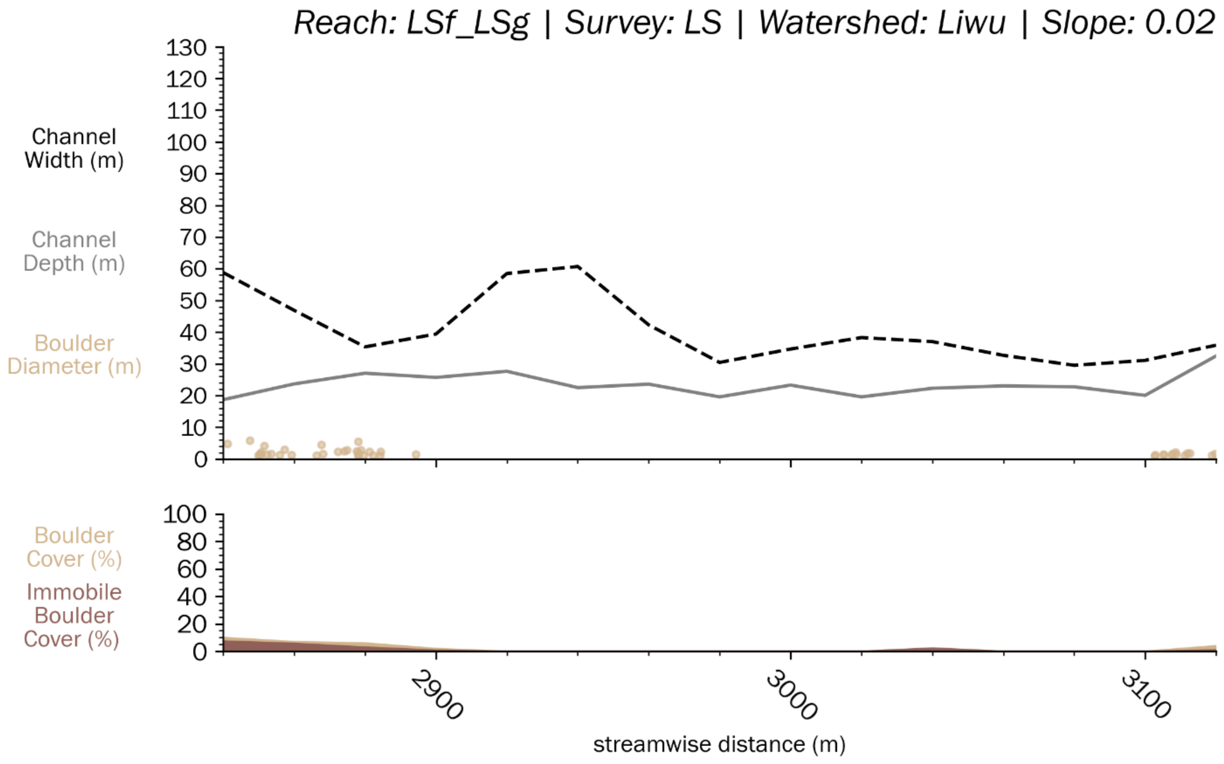

Reach: LSf_LSg | Survey: LS | Watershed: Liwu | Slope: 0.02
Channel Width (m)
Channel Depth (m)
Boulder Diameter (m)
Boulder Cover (%)
Immobile Boulder Cover (%)
130
120
110
100
90
80
70
60
50
40
30
20
10
0
100
80
60
40
20
0
2900
3000
3100
streamwise distance (m)

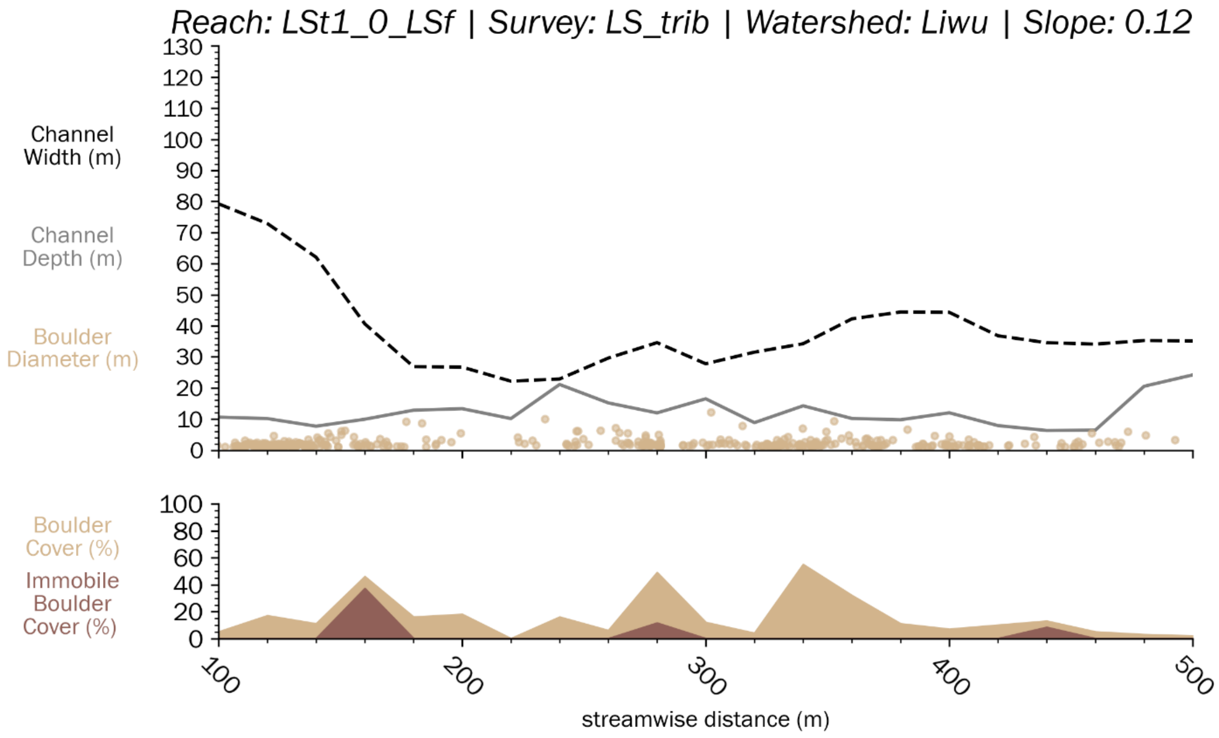

Reach: LSt1_0_LSf | Survey: LS_trib | Watershed: Liwu | Slope: 0.12
Channel Width (m)
Channel Depth (m)
Boulder Diameter (m)
130
120
110
100
90
80
70
60
50
40
30
20
10
0
Boulder Cover (%)
Immobile Boulder Cover (%)
100
80
60
40
20
0
100
200
300
400
500
streamwise distance (m)

Site: Xipan (XP), Liwu basin

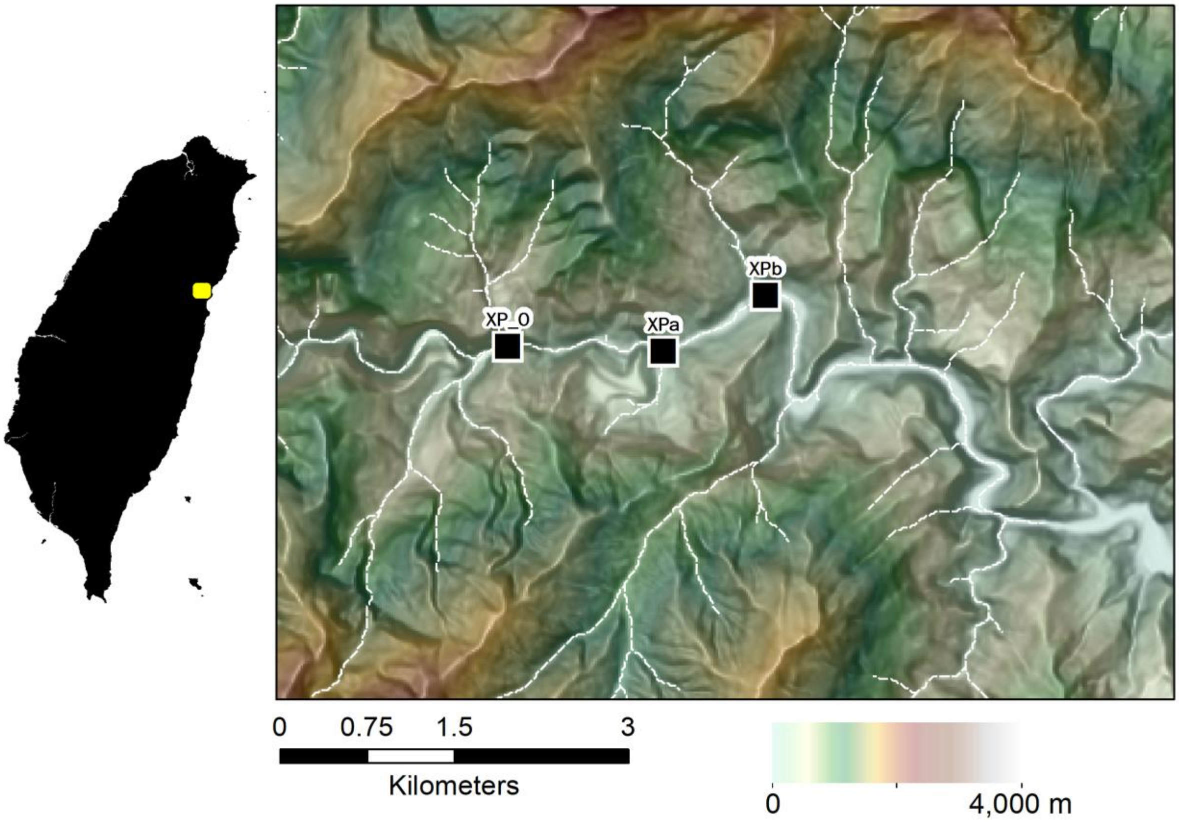

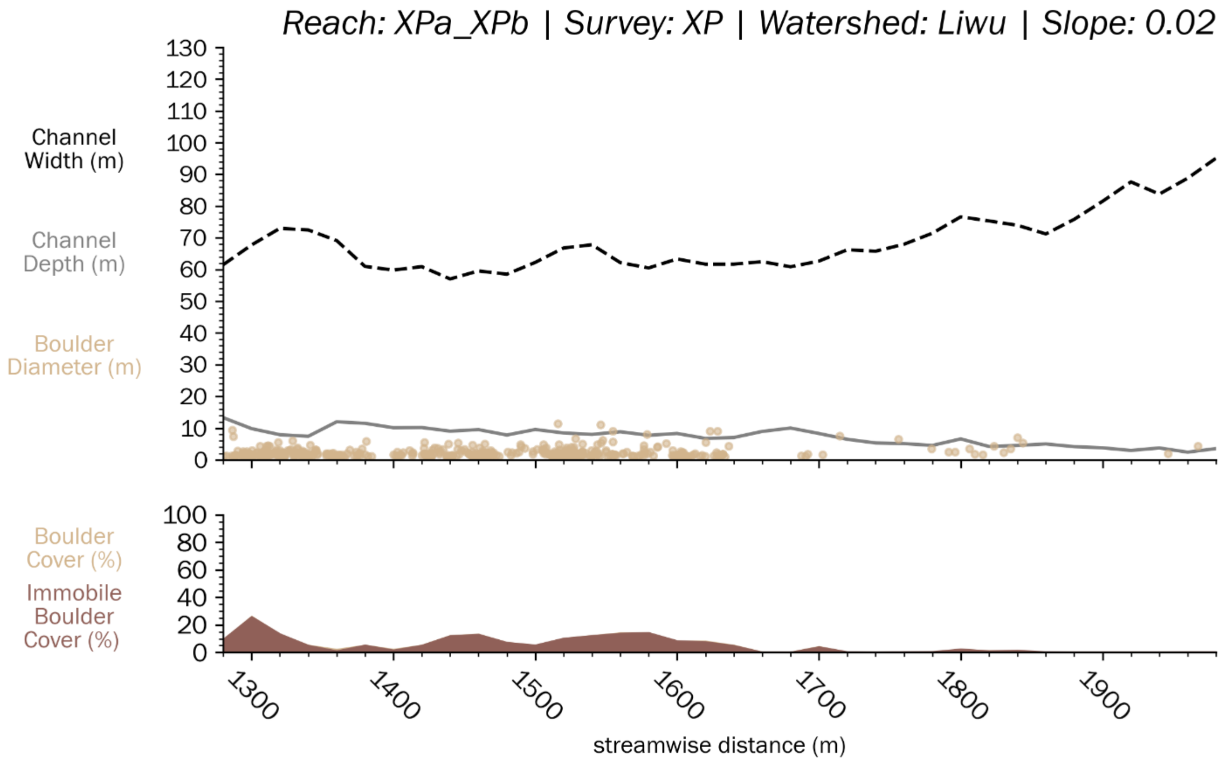
Reach: XPa_XPb | Survey: XP | Watershed: Liwu | Slope: 0.02
Channel Width (m)
Channel Depth (m)
Boulder Diameter (m)
Boulder Cover (%)
Immobile Boulder Cover (%)
streamwise distance (m)

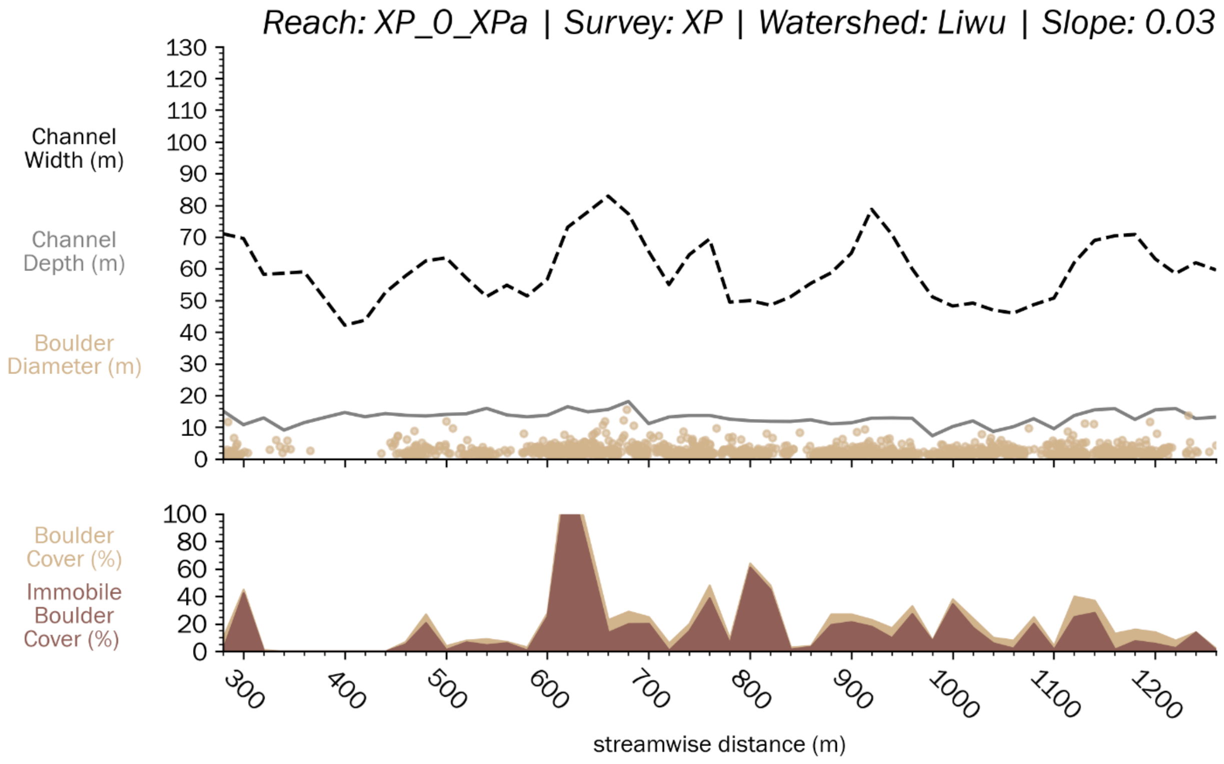
Reach: XP_0_XPa | Survey: XP | Watershed: Liwu | Slope: 0.03
Channel Width (m)
Channel Depth (m)
Boulder Diameter (m)
Boulder Cover (%)
Immobile Boulder Cover (%)
streamwise distance (m)
